# The Kreĭn Formula and its Weyl Families

**DOI:** 10.1007/s00020-026-02877-3

**Published:** 2026-11-13

**Authors:** Jussi Behrndt, Seppo Hassi, Henk de Snoo

**Affiliations:** 1 Institut für Angewandte Mathematik, Technische Universität Graz, Steyrergasse 30, 8010 Graz, Austria; 2 Department of Mathematics and Statistics, University of Vaasa, P.O. Box 700, FI-65200 Vaasa, Finland; 3 Bernoulli Institute for Mathematics, Computer Science and Artificial Intelligence, University of Groningen, P.O. Box 407, 9700 AK Groningen, Netherlands

**Keywords:** Symmetric operator, Self-adjoint extension, Boundary triplet, Boundary relation, Weyl function, Weyl family, Nevanlinna family, Reproducing kernel Hilbert space, Coupling method, 47A06, 47A20, 47A56, 47B25, 47B32, 47B44

## Abstract

Almost 50 years ago the well-known Kreĭn’s formula for the description of self-adjoint exit space extensions of a densely defined symmetric operator, originally established by M.G. Kreĭn in his famous 1946 paper, was presented by Langer and Textorius in the context of linear relations. The present paper offers a review of the parameters in the Kreĭn’s formula from the point of view of the general concept of boundary relations and their Weyl families. One of the main new results extends Kreĭn’s resolvent formula for an arbitrary boundary relation Γ of a symmetric operator (or relation) *S* whose Weyl family is a Nevanlinna family *Z*. Analogous to the case of the standard Kreĭn’s resolvent formula, which describes self-adjoint extensions of a symmetric operator *A* with equal defect numbers in a Hilbert space H, this Kreĭn type formula gives a one-to-one correspondence between (smooth) extensions $S_\Theta \subset {\text {dom }}\Gamma$ of $S=\ker \Gamma$ and parameters $\Theta =\Gamma (S _\Theta )$. When such a Kreĭn type formula is orthogonally coupled with the standard Kreĭn’s resolvent formula for self-adjoint extensions of *A* this yields an elementary proof of the Kreĭn-Naĭmark resolvent formula for the self-adjoint exit space extension of *A* generated by the Nevanlinna family *Z* by projecting the coupled formula to the space H.

## Introduction

Let *A* be a closed symmetric relation (multivalued operator) in a Hilbert space H and assume that *A* has equal defect numbers. Let $A_0$ be a fixed self-adjoint extension of *A* in H, then there exists a Hilbert space ${\mathcal {G}}$, a uniformly strict Nevanlinna function $M(\lambda ) \in {\textbf{B}}({\mathcal {G}})$, and a holomorphic function $\gamma ^\circ (\lambda ) \in {\textbf{B}}({\mathcal {G}}, \mathfrak H)$, such that the formula

1.1 $$\begin{aligned} (H-\lambda )^{-1} =(A_0-\lambda )^{-1}+\gamma ^\circ (\lambda ) \bigl (\Theta -M(\lambda )\bigr )^{-1} \gamma ^\circ (\bar{\lambda })^*, \quad \lambda \in {{\mathbb C}\setminus {\mathbb R}}, \end{aligned}$$

gives a one-to-one correspondence between the self-adjoint extensions *H* in H of *A* and the self-adjoint relations Θ in ${\mathcal {G}}$. Note that the parameter Θ is in general a multivalued operator, even if *A* is densely defined and its self-adjoint extensions are all operators. The identity in (1.1) is usually called Kreĭn’s formula. There is a more general version of Kreĭn’s formula of the form

1.2 $$\begin{aligned} P_{\mathfrak H}(\widetilde{H}-\lambda )^{-1}{\upharpoonright \,}{\mathfrak H}=(A_0-\lambda )^{-1}-\gamma ^\circ (\lambda ) \bigl (M(\lambda )+Z(\lambda )\bigr )^{-1} \gamma ^\circ (\bar{\lambda })^*, \quad \lambda \in {{\mathbb C}\setminus {\mathbb R}}. \end{aligned}$$

Now the left-hand side is the compression of the resolvent of a self-adjoint extension ${{\widetilde{H}} }$ of *A* in a larger Hilbert space ${{\widetilde{\mathfrak H}} }\supset {\mathfrak H}$, a so-called exit space extension, and *Z* stands for an, in general multivalued, Nevanlinna family in ${\mathcal {G}}$. A version of the Kreĭn formula in (1.2) was presented by Langer and Textorius in [31] in the context of linear relations *H* and ${{\widetilde{H}} }$. In their terminology the Nevanlinna function *M* was called a *Q*-function and it was not uniquely determined, see [30]. The present appearance of (1.1) and (1.2) is due to Derkach and Malamud [17, 18]; they have introduced the concept of Weyl function associated with the notion of a boundary triplet for $A^*$ for which the above functions *M* and $\gamma ^\circ$ are the (uniquely determined) Weyl function and the γ-field, respectively. A treatment of the Kreĭn formula in terms of an ordinary boundary triplet for $A^*$ can be found, for instance, in [5] and in [19]. Kreĭn’s resolvent formula has important consequences and applications, for instance, in the investigation of spectral theoretic properties of self-adjoint extensions in the original Hilbert space (called canonical extensions) as well as for exit spaces extensions. A brief sketch of its long history can be found in Sect. 8.

In this paper the aim is to explore the parameters *M* and *Z* that appear in Kreĭn’s formula (1.2). It is known that any uniformly strict ${\textbf{B}}({\mathcal {G}})$-valued Nevanlinna function can serve as a Weyl function of an ordinary boundary triplet for $A^*$, where *A* is some closed symmetric relation with equal defect numbers. On a more general level any Nevanlinna family can serve as a Weyl family of a boundary relation for $A^*$, but now the defect numbers of *A* are not necessarily equal. The notion of a boundary relation extends that of a boundary triplet; see [14–16]. This paper offers a brief, almost self-contained, survey of the notion of a boundary relation and its corresponding main transform which is a self-adjoint relation in an appropriate orthogonal sum of Hilbert spaces. The Weyl family is actually a Nevanlinna family: a multivalued operator family that generalizes the usual notion of a Nevanlinna function. In fact, by means of the theory of reproducing kernel Hilbert spaces it is shown that any Nevanlinna family is a Weyl family. As an illustration it is shown how the so-called generalized resolvents or, equivalently, the so-called Štraus families can be interpreted as Weyl families. The usual Kreĭn formula as in (1.1) remains valid in the wider context of boundary relations and their Weyl families: it is based on a careful interpretation of the identity itself.

The class of boundary relations has many interesting subclasses. In particular, the class of boundary relations whose Weyl family is in fact a bounded everywhere defined operator-valued Nevanlinna function will turn out to be very useful. It contains as a subclass the well-known ordinary boundary triplets that were mentioned above and which show up in the usual context of the Kreĭn formula. In order to treat the Kreĭn formula in the context of exit space extensions one needs a brief excursion into the orthogonal coupling of a pair of closed symmetric operators in different Hilbert spaces. A self-adjoint extension of an orthogonal sum of two symmetric operators may be determined via appropriate coupling conditions. Together with a pair of Kreĭn formulas of the form (1.1) in the Cartesian product Hilbert space and compressing this specific self-adjoint extension to one of the Hilbert spaces one may obtain the Kreĭn formula of the form (1.2). This method was used in [25–27] for some special cases with a *Q*-function approach; it was put in the framework of ordinary boundary triplets and boundary relations in [13–15].

In the present paper the orthogonal coupling technique of closed symmetric relations will be used in the new context of a pair of an ordinary boundary triplet and a general boundary relation. The key tool here is the generalization of the Kreĭn formula (1.1) to the general setting of arbitrary boundary relations: this is established in Theorem 7.1, by extending the approach used in [13]. In this framework of boundary triplets and boundary relations it is possible to define an appropriate self-adjoint extension of the orthogonal sum of two symmetric relations via simple coupling conditions, whose associated Weyl family is a bounded everywhere defined operator-valued function. This Weyl family is obtained with the corresponding Shmul’yan transform (obeying the same coupling conditions) to the orthogonal sum of the Weyl function *M* of the ordinary boundary triplet and the Weyl family *Z* of the boundary relation. After compressing the resolvent of this coupled self-adjoint extension to the initial Hilbert space one then obtains the required resolvent formula (1.2) in the general case of arbitrary Weyl families *Z*; see Theorem 8.2.

The contents of the paper will be briefly explained. In Sect. 2 the formal definitions of a Nevanlinna family and a Nevanlinna pair are given. Likewise, the definition of a boundary relation and its Weyl family can be found in Sect. 3; also the so-called main transform that associates a unique self-adjoint relation to the boundary relation is treated there. The main result that each Nevanlinna family is the Weyl family of a boundary relation can be found in Sect. 4. This result is based on the use of the reproducing kernel Hilbert space which is defined in terms of the corresponding Nevanlinna pair (instead of the use of the ε-method, a construction appearing in the work of Kreĭn and Langer [30], involving abstract elements). Štraus relations induced by self-adjoint exit space extensions of a symmetric relation are discussed in Sect. 5 from the point of view of boundary relations. The special class of boundary relations of bounded type are considered in Sect. 6; in particular, attention is paid to ordinary boundary triplets. The Kreĭn type formula appears in Sect. 7: it is now presented in the context of boundary relations, see  Theorem 7.1. Finally, in Sect. 8 the orthogonal coupling of an ordinary boundary triplet and a boundary relation is given. The orthogonal coupling technique in the framework of *Q*-functions (as in [30]) is briefly reviewed in App. A.

The following notations are used in this paper. For any Hilbert space H the inner product will be denoted by $(\cdot , \cdot )_{\mathfrak H}$. When there is a pair of Hilbert spaces H and K the Cartesian product is written as ${\mathfrak H}\times {\mathfrak K}$; and ${\mathfrak H}\times {\mathfrak H}$ is abbreviated to ${\mathfrak H}^2$. The inner product on the Cartesian product is given by the sum of the individual inner products. Sometimes the notation ${\mathfrak H}\oplus {\mathfrak K}$ will also be used. A linear relation *T* from a Hilbert space H to a Hilbert space K is by definition a linear subspace in ${\mathfrak H}\times {\mathfrak K}$; when ${\mathfrak K}={\mathfrak H}$ one also speaks of a linear relation in H. Throughout this paper an element in *T* will be denoted by $\{f,f'\}$ with $f \in {\mathfrak H}$ and $f' \in {\mathfrak K}$; also the abbreviation ${{\widehat{f}} }=\{f,f^\prime \}$ will be used. The domain, range, kernel, and multivalued part of a linear relation *T* are denoted by ${\text {dom }}T$, ${\text {ran }}T$, kerT, and $\mathrm{mul\,}T$, respectively. Sometimes for extra clarity self-explanatory vector notation will be used. For more details, see [5].

## Nevanlinna Families and Nevanlinna Pairs

The notion of a Nevanlinna family appearing in [31] is the one of the main objects of study in this survey paper; see also [5, Definition 1.12.1]. Here is a concise exposition.

### Definition 2.1

A family $Z=\{Z(\lambda ):\,\lambda \in {{\mathbb C}\setminus {\mathbb R}}\}$ of linear relations in a Hilbert space ${\mathcal {G}}$ is said to be a *Nevanlinna family* in ${\mathcal {G}}$ if

1. Z(λ) is maximal dissipative (maximal accumulative) for $\lambda \in {\mathbb C}^+$ ($\lambda \in {\mathbb C}^-$);
2. $$Z(\bar{\lambda })=Z(\lambda )^*$$ for all $$\lambda \in {\mathbb C}\setminus {\mathbb R}$$;
3. there exists $\mu \in {\mathbb C}^+$ such that $\lambda \mapsto (Z(\lambda )+\mu )^{-1}$ is holomorphic on ${\mathbb C}^+$ and $\lambda \mapsto (Z(\lambda )+\bar{\mu })^{-1}$ is holomorphic on ${\mathbb C}^-$.

For a Nevanlinna family *Z* the multivalued part $\mathrm{mul\,}Z(\lambda )$ and, by symmetry, the kernel $\ker Z(\lambda )$ do not depend on $\lambda \in {{\mathbb C}\setminus {\mathbb R}}$; see also Lemma 3.4 and [40, p. 188-189]. In particular, *Z* is an operator-valued function if and only if ${\text {dom }}Z(\lambda )$ is dense for some, and hence for all, $\lambda \in {{\mathbb C}\setminus {\mathbb R}}$.

Closely connected to a Nevanlinna family is the notion of a Nevanlinna pair; cf. [5, Definition 1.12.3].

### Definition 2.2

A pair $\{{\mathcal {A}}, {\mathcal {B}}\}$ of ${\textbf{B}}({\mathcal {G}})$-valued functions on ${{\mathbb C}\setminus {\mathbb R}}$ is said to be a *Nevanlinna pair* in ${\mathcal {G}}$ if

1. the mappings $\lambda \mapsto {\mathcal {A}}(\lambda )$ and $\lambda \mapsto {\mathcal {B}}(\lambda )$ are holomorphic on ${{\mathbb C}\setminus {\mathbb R}}$;
2. $$(\mathrm{Im\,}\lambda ) \mathrm{Im\,}({\mathcal {A}}(\lambda )^* {\mathcal {B}}(\lambda )) \ge 0$$, $$\lambda \in {{\mathbb C}\setminus {\mathbb R}}$$;
3. $${\mathcal {A}}(\bar{\lambda })^* {\mathcal {B}}(\lambda )={\mathcal {B}}(\bar{\lambda })^* {\mathcal {A}}(\lambda )$$, $$\lambda \in {{\mathbb C}\setminus {\mathbb R}}$$;
4. there exist $\mu \in {\mathbb C}^+$ such that $({\mathcal {B}}(\lambda )+\mu {\mathcal {A}}(\lambda ))^{-1} \in \textbf{B}({\mathcal {G}})$ for $\lambda \in {\mathbb C}^+$ and $({\mathcal {B}}(\lambda )+{\bar{\mu }} {\mathcal {A}}(\lambda ))^{-1} \in \textbf{B}({\mathcal {G}})$ for $\lambda \in {\mathbb C}^-$.

Due to $\mathrm {(P4)}$ a Nevanlinna pair $\{{\mathcal {A}},{\mathcal {B}}\}$ automatically satisfies

2.1 $$\begin{aligned} \ker {\mathcal {A}}(\lambda ) \cap \ker {\mathcal {B}}(\lambda )=\{0\}, \quad \lambda \in {{\mathbb C}\setminus {\mathbb R}}. \end{aligned}$$

This obvious result plays an important role in the following proposition about the connection between Nevanlinna families and Nevanlinna pairs; cf. [5, Proposition 1.12.2, Proposition 1.12.4].

### Proposition 2.3

Let *Z* be a Nevanlinna family in ${\mathcal {G}}$. Then there exists a Nevanlinna pair $\{{\mathcal {A}},{\mathcal {B}}\}$ in ${\mathcal {G}}$ such that

2.2 $$\begin{aligned} Z(\lambda )=\bigl \{ \{{\mathcal {A}}(\lambda )h, {\mathcal {B}}(\lambda )h\}:\, h \in {\mathcal {G}}\bigr \}, \quad \lambda \in {{\mathbb C}\setminus {\mathbb R}}. \end{aligned}$$

Conversely, let $\{{\mathcal {A}},{\mathcal {B}}\}$ be a Nevanlinna pair in ${\mathcal {G}}$, then there exists a Nevanlinna family *Z* in ${\mathcal {G}}$ such that (2.2) holds.

Moreover, a Nevanlinna pair $\{{\mathcal {A}}, {\mathcal {B}}\}$ in ${\mathcal {G}}$ such that (2.2) holds is unique up to right multiplication by a bounded and boundedly invertible holomorphic operator family ${\mathcal {X}}(\lambda )$, $\lambda \in {{\mathbb C}\setminus {\mathbb R}}$, in ${\mathcal {G}}$.

Let the Nevanlinna family *Z* and the Nevanlinna pair $\{{\mathcal {A}},{\mathcal {B}}\}$ be connected by (2.2). Then one also has the representation

2.3 $$\begin{aligned} Z(\lambda )=\big \{ \{\varphi , \varphi '\} \in {\mathcal {G}}^2:\, {\mathcal {B}}(\bar{\lambda })^* \varphi = {\mathcal {A}}(\bar{\lambda })^* \varphi ' \big \}. \end{aligned}$$

To see this, use the property $Z(\lambda )=Z(\bar{\lambda })^*$ from (F2) and calculate the right-hand side by taking the adjoint in (2.2) with λ replaced by $\bar{\lambda }$.

It is not difficult to show that the condition (F3) may be replaced by the following condition

(F3’) $\lambda \mapsto (Z(\lambda )+\lambda )^{-1} \in {\textbf{B}}({\mathcal {G}})$ is holomorphic on ${{\mathbb C}\setminus {\mathbb R}}$,

and that the condition (P4) may be replaced by

(P4’) $({\mathcal {B}}(\lambda )+\lambda {\mathcal {A}}(\lambda ))^{-1} \in {\textbf{B}}({\mathcal {G}})$ and $\lambda \mapsto ({\mathcal {B}}(\lambda )+\lambda {\mathcal {A}}(\lambda ))^{-1}$ are holomorphic on ${{\mathbb C}\setminus {\mathbb R}}$,

see [5, Proposition 1.12.6]. Moreover, if the Nevanlinna family *Z* and the Nevanlinna pair $\{{\mathcal {A}}, {\mathcal {B}}\}$ are connected by (2.2), then

2.4 $$\begin{aligned} (Z(\lambda )+\lambda )^{-1}={\mathcal {A}}(\lambda ) ({\mathcal {B}}(\lambda )+\lambda {\mathcal {A}}(\lambda ))^{-1}, \quad \lambda \in {{\mathbb C}\setminus {\mathbb R}}. \end{aligned}$$

Conversely, if *Z* is a Nevanlinna family, then a corresponding Nevanlinna pair is provided by

2.5 $$\begin{aligned} {\mathcal {A}}(\lambda )=(Z(\lambda )+\lambda )^{-1}, \quad {\mathcal {B}}(\lambda )=I - \lambda (Z(\lambda )+\lambda )^{-1}, \end{aligned}$$

which agrees with (2.4). These observations are also useful in a slightly more general setting in which the sum $Z(\lambda )+\lambda$ is replaced by the sum $Z(\lambda )+M(\lambda )$, where *M* is an operator-valued Nevanlinna function from the following class.

### Definition 2.4

A Nevanlinna function $M:\, {{\mathbb C}\setminus {\mathbb R}}\rightarrow {\textbf{B}}({\mathcal {G}})$ is said to be *uniformly strict* if $0\in \rho (\mathrm{Im\,}M(\lambda ))$ for some, and hence for all, $\lambda \in {{\mathbb C}\setminus {\mathbb R}}$.

Now let the Nevanlinna family *Z* correspond to the Nevanlinna pair $\{{\mathcal {A}},{\mathcal {B}}\}$. Then for any uniformly strict operator-valued Nevanlinna function *M* it follows that

2.6 $$\begin{aligned} -(Z(\lambda ) +M(\lambda ))^{-1}=-{\mathcal {A}}(\lambda )({\mathcal {B}}(\lambda )+M(\lambda ){\mathcal {A}}(\lambda ))^{-1} \in {\textbf{B}}({\mathcal {G}}), \quad \lambda \in {{\mathbb C}\setminus {\mathbb R}}, \end{aligned}$$

defines an operator-valued Nevanlinna function; see [5, Proposition 1.12.6]. Expressions as in (2.6) play a role in the Kreĭn formula in Sect. 7.

Likewise, in the case of coupling of boundary relations and boundary triplets as in Sect. 8, one needs the following construction, see, in particular, Lemma 8.1. For the Nevanlinna family *Z* and the uniformly strict operator-valued Nevanlinna function *M* one may define the block relation in ${\mathcal {G}}\times {\mathcal {G}}$ by

2.7 $$\begin{aligned} \Omega (\lambda )  &   =\begin{pmatrix} M(\lambda ) &  -I \\ -I &  -Z(\lambda )^{-1} \end{pmatrix} \\  &   \hspace{0cm} =\left\{ \left\{ \left( {\begin{array}{c}\varphi \\ \psi ^\prime \end{array}}\right) , \left( {\begin{array}{c}M(\lambda )\varphi - \psi ^\prime \\ -\varphi -\psi \end{array}}\right) \right\} :\, \varphi \in {\mathcal {G}},\, \{\psi ,\psi ^\prime \}\in Z(\lambda ) \right\} , \quad \lambda \in {{\mathbb C}\setminus {\mathbb R}}, \nonumber \end{aligned}$$

in the sense of [28]. It is clear from the definition that $\Omega (\lambda )$, $\lambda \in {{\mathbb C}\setminus {\mathbb R}}$, is a Nevanlinna family in ${\mathcal {G}}\times {\mathcal {G}}$.

### Lemma 2.5

Let *Z* be a Nevanlinna family in ${\mathcal {G}}$, let *M* be a uniformly strict ${\textbf{B}}({\mathcal {G}})$-valued Nevanlinna function, and let the block relation Ω be defined by (2.7). Then $-\Omega (\lambda )^{-1}$, $\lambda \in {{\mathbb C}\setminus {\mathbb R}}$, given by

2.8 $$\begin{aligned} \hspace{0.5cm} \begin{pmatrix} -(Z(\lambda )+M(\lambda ))^{-1} &  I-(Z(\lambda )+M(\lambda ))^{-1}M(\lambda ) \\ I- M(\lambda )(Z(\lambda )+M(\lambda ))^{-1} &  M(\lambda )- M(\lambda )(Z(\lambda )+M(\lambda ))^{-1}M(\lambda ) \end{pmatrix},\nonumber \\ \end{aligned}$$

is a ${\textbf{B}}({\mathcal {G}}\times {\mathcal {G}})$-valued Nevanlinna function.

### Proof

Denote the block operator in (2.8) by K(λ), thus $K(\lambda )\in {\textbf{B}}({\mathcal {G}}\times {\mathcal {G}})$. Note that by (2.7) one may write $-\Omega (\lambda )^{-1}$ as follows

2.9 $$\begin{aligned} -\Omega (\lambda )^{-1}=\left\{ \left\{ \left( {\begin{array}{c}-M(\lambda )\varphi +\psi ^\prime \\ \varphi +\psi \end{array}}\right) , \left( {\begin{array}{c}\varphi \\ \psi ^\prime \end{array}}\right) \right\} :\, \varphi \in {\mathcal {G}},\, \{\psi ,\psi ^\prime \}\in Z(\lambda ) \right\} . \end{aligned}$$

First it will be shown that $-\Omega (\lambda )^{-1} \subset K(\lambda )$. Setting $h:=-M(\lambda )\varphi +\psi ^\prime$ and $k:=\varphi +\psi$ one has $\varphi =k-\psi$, so that

$$ \{\psi , \psi '\}=\{\psi , h+M(\lambda )(k-\psi )\} \in Z(\lambda ) \quad \text{ or } \quad \{\psi , h+M(\lambda )k\} \in Z(\lambda )+M(\lambda ). $$

Therefore $\{h+M(\lambda )k,\psi \}\in (Z(\lambda )+M(\lambda ))^{-1}$, and $\varphi =k-\psi$ leads to

2.10 $$\begin{aligned} \varphi =-(Z(\lambda )+M(\lambda ))^{-1}h + (I- (Z(\lambda )+M(\lambda ))^{-1}M(\lambda ))k. \end{aligned}$$

Furthermore, $\psi ^\prime =h+M(\lambda )\varphi$ leads to

2.11 $$\begin{aligned} \psi ^\prime =(I-M(\lambda )(Z(\lambda )+M(\lambda ))^{-1})h + M(\lambda )(I-(Z(\lambda )+M(\lambda ))^{-1}M(\lambda ))k. \end{aligned}$$

Then (2.10) and (2.11) show the inclusion $-\Omega (\lambda )^{-1}\subseteq K(\lambda )$.

To prove the equality, recall that $K(\lambda ) \in {\textbf{B}}({\mathcal {G}}\times {\mathcal {G}})$ and that $-\Omega (\lambda )^{-1}\subset K(\lambda )$. Thus it suffices to show that

$$ {\text {dom }}(-\Omega (\lambda )^{-1})={\mathcal {G}}\times {\mathcal {G}}. $$

For this purpose, let $h,k\in {\mathcal {G}}$ and define φ and $\psi ^\prime$ via (2.10) and (2.11). Then $\psi ^\prime =h+M(\lambda )\varphi$ and define ψ by

$$ \psi :=k-\varphi =(Z(\lambda )+M(\lambda ))^{-1}(h+M(\lambda )k). $$

Hence, $\{\psi ,h+M(\lambda )k\}\in Z(\lambda )+M(\lambda )$ and since

$$ h+M(\lambda )(k-\psi )=h+M(\lambda )\varphi =\psi ', $$

it follows that $\{\psi , \psi '\} \in Z(\lambda )$. Now observe that $h=-M(\lambda )\varphi +\psi '$ and $k=\varphi +\psi$, so that $\{h,k\} \in {\text {dom }}(-\Omega (\lambda )^{-1})$. Thus ${\text {dom }}(-\Omega (\lambda )^{-1})={\mathcal {G}}\times {\mathcal {G}}$ and $-\Omega (\lambda )^{-1}=K(\lambda )$.

Since clearly $\Omega (\lambda )$, $\lambda \in {{\mathbb C}\setminus {\mathbb R}}$, is a Nevanlinna family in ${\mathcal {G}}\times {\mathcal {G}}$, it follows that $K(\lambda )=-\Omega (\lambda )^{-1}$, $\lambda \in {{\mathbb C}\setminus {\mathbb R}}$, is a ${\textbf{B}}({\mathcal {G}}\times {\mathcal {G}})$-valued Nevanlinna function. □

Implementing the expression from (2.6) in the block formula (2.8) allows to rewrite the Nevanlinna function $-\Omega (\lambda )^{-1}$ in terms of the operators M(λ), ${\mathcal {A}}(\lambda )$, and ${\mathcal {B}}(\lambda )$; cf. [15, Theorem 5.12].

## Boundary Relations and Weyl Families

The notions of a boundary relation and its corresponding Weyl family go back to [14, 15]; see also [11, 12]. The goal of this section and the next section is to review relevant definitions and some elementary properties of boundary relations. It will be shown that the class of Weyl families consists of Nevanlinna families and, moreover, each Nevanlinna family is a Weyl family. The connection of boundary relations with the usual ordinary boundary triplets will be discussed in Sect. 6.

### Some Definitions and Basic Properties

The definition of a boundary relation is associated with a closed linear symmetric relation *S* in a Hilbert space H. It should be stressed that there is no condition on the defect numbers of *S*; in general they need not be equal.

#### Definition 3.1

Let *S* be a closed symmetric relation in a Hilbert space H and let ${\mathcal {G}}$ be a Hilbert space. A linear relation Γ from ${\mathfrak H}^2$ to ${\mathcal {G}}^2$ is called a *boundary relation* for $S^*$ if:

1. ${\text {dom }}\Gamma$ is dense in $S^*$;
2. the abstract Green’s identity
  3.1 $$\begin{aligned} (f^\prime ,g)_{\mathfrak H}-(f,g^\prime )_{\mathfrak H}=(\varphi ^\prime , \psi )_{\mathcal {G}}-(\varphi , \psi ^\prime )_{\mathcal {G}}\end{aligned}$$
  holds for all $\{{{\widehat{f}} },{{\widehat{\varphi }} }\}$, $\{{{\widehat{g}} },{{\widehat{\psi }} }\}\in \Gamma$;
3. if $\{{{\widehat{g}} },{{\widehat{\psi }} }\}\in {\mathfrak H}^2\times {\mathcal {G}}^2$ satisfies the abstract Green’s identity (3.1) for every $\{{{\widehat{f}} },{{\widehat{\varphi }} }\}\in \Gamma$, then $\{{{\widehat{g}} },{{\widehat{\psi }} }\}\in \Gamma$.

It is clear from (D2) and (D3) that the boundary relation Γ is closed, i.e., its graph as a subspace of ${\mathfrak H}^2 \oplus {\mathcal {G}}^2$ is closed. By definition the adjoint $\Gamma ^*$ of Γ is the set of all $\{ {{\widehat{\psi }} }, {{\widehat{g}} }\}$ in ${\mathcal {G}}^2 \times {\mathfrak H}^2$ for which $({{\widehat{g}} }, {{\widehat{f}} }\,)_{{\mathfrak H}^2}=({{\widehat{\psi }} }, {{\widehat{\varphi }} }\,)_{{\mathcal {G}}^2}$ for all $\{{{\widehat{f}} },{{\widehat{\varphi }} }\}\in \Gamma$. Hence it follows from (D3) that

3.2 $$\begin{aligned} \Gamma ^*=\big \{ \{{{\widehat{\psi }} }, {{\widehat{g}} }\} \in {\mathcal {G}}^2 \times {\mathfrak H}^2:\, \big \{ \{g', -g\}, \{\psi ', -\psi \} \big \} \in \Gamma \big \}, \end{aligned}$$

and as a consequence of (3.2) one sees that

3.3 $$\begin{aligned} \begin{aligned} \{\psi , \psi '\} \in {\text {dom }}\Gamma ^*&\quad \Leftrightarrow \quad \{\psi ', -\psi \} \in {\text {ran }}\Gamma , \\ \{g, g'\} \in {\text {ran }}\Gamma ^*&\quad \Leftrightarrow \quad \{g', -g\} \in {\text {dom }}\Gamma . \end{aligned} \end{aligned}$$

Since Γ is closed, one has that ${\text {dom }}\Gamma ^*$ or ${\text {ran }}\Gamma ^*$ is closed if and only if ${\text {dom }}\Gamma$ or ${\text {ran }}\Gamma$ is closed, respectively; cf. [5, Theorem 1.3.5]. Therefore it follows from (3.3) that

3.4 $$\begin{aligned} {\text {dom }}\Gamma \, \text{ is } \text{ closed } \quad \Leftrightarrow \quad {\text {ran }}\Gamma \, \text{ is } \text{ closed }, \end{aligned}$$

see [34], in which case ${\text {dom }}\Gamma =S^*$. By direct verification one can show that

3.5 $$\begin{aligned} ({\text {dom }}\Gamma )^*=\ker \Gamma \quad \text{ and } \quad ({\text {ran }}\Gamma )^*=\mathrm{mul\,}\Gamma . \end{aligned}$$

For instance, if ${{\widehat{g}} } \in ({\text {dom }}\Gamma )^*$, then $\{{{\widehat{g}} }, \{0,0\}\}$ satisfies (3.1) for all $\{{{\widehat{f}} }, {{\widehat{\varphi }} }\} \in \Gamma$, so that by (D3) one has $\{{{\widehat{g}} }, \{0,0\}\} \in \Gamma$, and for the converse a similar argument can be used. In particular, if ${\text {ran }}\Gamma ={\mathcal {G}}^2$, then ${\text {dom }}\Gamma =S^*$ and $\mathrm{mul\,}\Gamma =\{0,0\}$.

Note that the condition (D1) means that $$S^{*}=({\text {dom }}\Gamma )^{**}$$ or, since *S* is assumed to be closed, $$S=({\text {dom }}\Gamma )^*$$. Due to (3.5), the last identity is equivalent to the basic identity

3.6 $$\begin{aligned} S=\ker \Gamma . \end{aligned}$$

Next introduce the linear relation ${\mathcal {T}}$ in H by

3.7 $$\begin{aligned} {\mathcal {T}}={\text {dom }}\Gamma . \end{aligned}$$

In general ${\mathcal {T}}$ is not equal to $S^*$ and ${\mathcal {T}}$ can be considered as a *smooth version* of $S^*$, since Γ carries information only from ${\mathcal {T}}$ to the (boundary) space ${\mathcal {G}}^2$. Clearly, by (3.6) and (3.7) one has

3.8 $$\begin{aligned} S \subset {\mathcal {T}}\subset ({\mathcal {T}})^{**}=S^*. \end{aligned}$$

In order to deal with boundary relations the following suggestive notation is useful. Let $S \subset H \subset {\mathcal {T}}={\text {dom }}\Gamma \subset {\mathfrak H}^2$ be a linear subspace in ${\mathfrak H}^2$, then define

3.9 $$\begin{aligned} \Gamma (H)=\big \{ {{\widehat{\varphi }} }\in {\mathcal {G}}^2:\, \{{{\widehat{f}} }, {{\widehat{\varphi }} }\} \in \Gamma , \,\, {{\widehat{f}} } \in H \big \}, \end{aligned}$$

so that automatically

3.10 $$\begin{aligned} \mathrm{mul\,}\Gamma \subset \Gamma (H) \subset {\text {ran }}\Gamma ; \end{aligned}$$

when no confusion appears, the shorter notation ΓH is used instead of Γ(H). Likewise, let $\mathrm{mul\,}\Gamma \subset \Theta \subset {\text {ran }}\Gamma \subset {\mathcal {G}}^2$ be a linear subspace in ${\mathcal {G}}^2$, then define

3.11 $$\begin{aligned} \Gamma ^{-1}(\Theta )=\big \{ {{\widehat{f}} } \in {\mathfrak H}^2:\, \{{{\widehat{f}} }, {{\widehat{\varphi }} }\} \in \Gamma , \,\, {{\widehat{\varphi }} }\in \Theta \big \}, \end{aligned}$$

so that automatically

3.12 $$\begin{aligned} \ker \Gamma \subset \Gamma ^{-1}(\Theta ) \subset {\text {dom }}\Gamma . \end{aligned}$$

With these notations established, one has as a consequence of (3.9) and (3.11) that

3.13 $$\begin{aligned} \Gamma ^{-1}( \Gamma (H))=H \quad \text{ and } \quad \Gamma (\Gamma ^{-1}(\Theta ))=\Theta . \end{aligned}$$

A relation *H* that satisfies $S \subset H \subset {\mathcal {T}}$ will be called a *smooth extension* of *S*. The next result is now clear.

#### Corollary 3.2

Let *S* be a closed symmetric relation in H and let Γ be a boundary relation from ${\mathfrak H}^2$ to ${\mathcal {G}}^2$ for $S^*$. Then Γ provides a one-to-one correspondence between the linear subspaces *H* in ${\mathfrak H}^2$ with $S \subset H \subset {\mathcal {T}}={\text {dom }}\Gamma$ and the linear subspaces Θ in ${\mathcal {G}}^2$ with $\mathrm{mul\,}\Gamma \subset \Theta \subset {\text {ran }}\Gamma$.

Therefore, if $\mathrm{mul\,}\Gamma$ is trivial, then Γ provides a one-to-one correspondence between all linear subspaces *H* with $S \subset H \subset {\mathcal {T}}$ and all linear subspaces in ${\mathcal {G}}^2$. In particular, if ${\text {dom }}\Gamma =S^*$ or, equivalently, ${\text {ran }}\Gamma ={\mathcal {G}}^2$, then Γ provides a one-to-one correspondence between all linear subspaces *H* with $S \subset H \subset S^*$, called *intermediate extensions* *H* of *S*, and all linear subspaces in ${\mathcal {G}}^2$.

For $\lambda \in {{\mathbb C}\setminus {\mathbb R}}$ the smooth eigenspace $\ker ({\mathcal {T}}-\lambda ) \subset {\mathfrak H}$ of ${\mathcal {T}}$ will be denoted by ${\mathfrak N}_\lambda ({\mathcal {T}})$. Associated with it is the eigenspace (in the graph sense)

3.14 $$\begin{aligned} {{\widehat{\mathfrak N}} }_{\lambda }({\mathcal {T}}) =\bigl \{ {{\widehat{f}} }_\lambda :\, f_\lambda \in {\mathfrak N}_\lambda ({\mathcal {T}})\bigr \}, \quad \text{ where } \quad {{\widehat{f}} }_\lambda =\{f_\lambda , \lambda f_\lambda \}, \end{aligned}$$

so that ${{\widehat{\mathfrak N}} }_{\lambda }({\mathcal {T}}) \subset {\mathfrak H}^2$. Moreover, ${\mathfrak N}_{\lambda }({\mathcal {T}})$ is dense in ${\mathfrak N}_\lambda (S^*)$ for all $\lambda \in {{\mathbb C}\setminus {\mathbb R}}$; cf. [14, Lemma 2.14]. Observe that

3.15 $$\begin{aligned} \Gamma {\upharpoonright \,}{{\widehat{\mathfrak N}} }_\lambda ({\mathcal {T}}) =\big \{ \{{{\widehat{f}} }_{\lambda },{{\widehat{\varphi }} }\} \in \Gamma :\, {{\widehat{f}} }_{\lambda } \in {{\widehat{\mathfrak N}} }_{\lambda }({\mathcal {T}}) \big \}. \end{aligned}$$

For all elements $\{{{\widehat{f}} }_{\lambda },{{\widehat{\varphi }} }\}, \{{{\widehat{g}} }_{\mu },{{\widehat{\psi }} }\} \in \Gamma$ it follows from (3.1) that

3.16 $$\begin{aligned} (\lambda -\bar{\mu })(f_{\lambda },g_{\mu })_{\mathfrak H}=(\varphi ^\prime , \psi )_{\mathcal {G}}-(\varphi , \psi ^\prime )_{\mathcal {G}}. \end{aligned}$$

#### Definition 3.3

Let *S* be a closed symmetric relation in H and let Γ be a boundary relation from ${\mathfrak H}^2$ to ${\mathcal {G}}^2$ for $S^*$. The *Weyl family* of *S* corresponding to Γ is a family $Z=\{Z(\lambda ):\lambda \in {{\mathbb C}\setminus {\mathbb R}}\}$ of linear relations in ${\mathcal {G}}$, given by $Z(\lambda )=\Gamma ({{\widehat{\mathfrak N}} }_\lambda ({\mathcal {T}}))$, i.e.,

3.17 $$\begin{aligned} Z(\lambda ) := \bigl \{{{\widehat{\varphi }} }\,:\,\{{{\widehat{f}} }_{\lambda },{{\widehat{\varphi }} }\}\in \Gamma ,\, {{\widehat{f}} }_{\lambda } \in {{\widehat{\mathfrak N}} }_{\lambda }({\mathcal {T}})\bigr \}, \quad \lambda \in {\mathbb C}\setminus {\mathbb R}. \end{aligned}$$

Associated with a boundary relation Γ from ${\mathfrak H}^2$ to ${\mathcal {G}}^2$ are the linear relations $\Gamma _0$ and $\Gamma _1$ from ${\mathfrak H}^2$ to ${\mathcal {G}}$ via the first and second component of Γ by

3.18 $$\begin{aligned} \Gamma _0=\big \{ \{{{\widehat{f}} }, \varphi \} \in {\mathfrak H}^2 \times {\mathcal {G}}:\, \{{{\widehat{f}} }, {{\widehat{\varphi }} }\} \in \Gamma \big \} \end{aligned}$$

and

3.19 $$\begin{aligned} \Gamma _1=\big \{ \{{{\widehat{f}} }, \varphi ' \} \in {\mathfrak H}^2 \times {\mathcal {G}}:\, \{{{\widehat{f}} }, {{\widehat{\varphi }} }\} \in \Gamma \big \}. \end{aligned}$$

The corresponding relations $S_0$ and $S_1$ in H are defined by

3.20 $$\begin{aligned} S_0=\ker \Gamma _0=\big \{ {{\widehat{f}} } :\, \{{{\widehat{f}} }, \{0, \varphi '\}\} \in \Gamma \,\, \text{ for } \text{ some } \,\, \varphi ' \in {\mathcal {G}}\big \}, \end{aligned}$$

and

3.21 $$\begin{aligned} S_1=\ker \Gamma _1=\big \{ {{\widehat{f}} } :\, \{{{\widehat{f}} }, \{\varphi , 0\} \} \in \Gamma \,\, \text{ for } \text{ some } \,\, \varphi \in {\mathcal {G}}\big \}. \end{aligned}$$

Then $S_0$ and $S_1$ are symmetric extensions of the closed symmetric relation $S=\ker \Gamma$ in (3.6). Note that $S_0$ and $S_1$ are not necessarily closed; see [14], where a slightly different notation has been used.

The following results connect the multivalued part of the boundary relation Γ to certain invariance properties of the corresponding Weyl family *Z*; the simple lemma goes back to [14, Lemma 4.1].

#### Lemma 3.4

Let Γ be a boundary relation from ${\mathfrak H}^2$ to ${\mathcal {G}}^2$ with the Weyl family *Z*. Then the following equalities hold for all $\lambda \in {{\mathbb C}\setminus {\mathbb R}}$:

- $$Z(\lambda )\cap Z(\lambda )^*=\mathrm{mul\,}\Gamma $$;
- $$\ker Z(\lambda )\times \{0\}=\mathrm{mul\,}\Gamma \cap ({\mathcal {G}}\times \{0\})$$;
- $$\{0\}\times \mathrm{mul\,}Z(\lambda )=\mathrm{mul\,}\Gamma \cap (\{0\}\times {\mathcal {G}})$$;
- $$\ker (Z(\lambda )-Z(\lambda )^*)=\mathrm{mul\,}\Gamma _0$$;
- $$\ker (Z(\lambda )^{-1}-Z(\lambda )^{-*})=\mathrm{mul\,}\Gamma _1$$.

Lemma 3.4 shows explicitly that the multivalued part of Γ appears as the constant part of the corresponding Weyl family Z(λ) by property (a) and, in particular, that the kernel and the multivalued part of Z(λ) are constant by (b) and (c), in addition, also the imaginary part of Z(λ) and the imaginary part of the inverse $Z(\lambda )^{-1}$ are constant by (d) and (e), respectively. Conversely, if the Weyl family (or its inverse) has a nonzero kernel or its imaginary part has a nonzero kernel at one point $\lambda \in {{\mathbb C}\setminus {\mathbb R}}$, then the corresponding boundary relation is necessarily a multivalued mapping. The same holds in particular for operator-valued Weyl functions.

An important special case arises when the boundary relation Γ in Definition 3.1 is single-valued, in which case also the component mappings $\Gamma _0$ and $\Gamma _1$ in (3.18) and (3.19) are single-valued. This leads to the following definition.

#### Definition 3.5

A boundary relation Γ from ${\mathfrak H}^2$ to ${\mathcal {G}}^2$ is said to be a *boundary triplet* if $\mathrm{mul\,}\Gamma =\{0,0\}$ or, equivalently, ${\text {ran }}\Gamma$ is dense in ${\mathcal {G}}^2$. The boundary triplet is denoted by $\{{\mathcal {G}},\Gamma _0,\Gamma _1\}$.

In the case of Definition 3.5 the Weyl family in Definition 3.3 becomes an operator-valued function which is then called the *Weyl function* of *S* corresponding to the boundary triplet $\{{\mathcal {G}},\Gamma _0,\Gamma _1\}$. Moreover, in this case the Weyl function can be also defined by the equality

$$ \Gamma _1 {{\widehat{f}} }_{\lambda } = Z(\lambda ) \Gamma _0 {{\widehat{f}} }_{\lambda }, \quad {{\widehat{f}} }_{\lambda } \in {{\widehat{\mathfrak N}} }_{\lambda }({\mathcal {T}}),\quad \lambda \in {{\mathbb C}\setminus {\mathbb R}}. $$

The usual ordinary boundary triplet, when the Weyl function is uniformly strict, is briefly introduced in Sect. 6.

With the linear relation $\Gamma _0$ it is clear for $\lambda \in {{\mathbb C}\setminus {\mathbb R}}$ that

3.22 $$\begin{aligned} \Gamma _0 {\upharpoonright \,}{{\widehat{\mathfrak N}} }_\lambda ({\mathcal {T}}) =\big \{ \{{{\widehat{f}} }_{\lambda }, \varphi \} \in {\mathfrak H}^2 \times {\mathcal {G}}:\, \{{{\widehat{f}} }_\lambda , {{\widehat{\varphi }} }\} \in \Gamma , \,\, {{\widehat{f}} }_{\lambda } \in {{\widehat{\mathfrak N}} }_{\lambda }({\mathcal {T}}) \big \}. \end{aligned}$$

Next define ${{\widehat{\gamma }} }(\lambda )$ as the inverse of (3.22) by

3.23 $$\begin{aligned} \begin{aligned} {{\widehat{\gamma }} }(\lambda )&=\big (\Gamma _0 {\upharpoonright \,}{{\widehat{\mathfrak N}} }_\lambda ({\mathcal {T}})\big )^{-1}\\&=\big \{ \{\varphi , {{\widehat{f}} }_{\lambda } \} \in {\mathcal {G}}\times {\mathfrak H}^2 :\, \{{{\widehat{f}} }_\lambda , {{\widehat{\varphi }} }\} \in \Gamma , \,\, {{\widehat{f}} }_{\lambda } \in {{\widehat{\mathfrak N}} }_{\lambda }({\mathcal {T}}) \big \}, \end{aligned} \end{aligned}$$

and note that

3.24 $$\begin{aligned} {\text {dom }}{{\widehat{\gamma }} }(\lambda )={\text {dom }}Z(\lambda ) \quad \text{ and } \quad {\text {ran }}{{\widehat{\gamma }} }(\lambda )={{\widehat{\mathfrak N}} }_\lambda ({\mathcal {T}}), \quad \lambda \in {{\mathbb C}\setminus {\mathbb R}}, \end{aligned}$$

see (3.17). From (3.16) it is clear that ${{\widehat{\gamma }} }(\lambda )$ is a single-valued mapping for all $\lambda \in {{\mathbb C}\setminus {\mathbb R}}$. Moreover, it follows from (3.18) and (3.22) that

3.25 $$\begin{aligned} \ker {{\widehat{\gamma }} }(\lambda )=\mathrm{mul\,}\Gamma _0=\ker \mathrm{Im\,}Z(\lambda ), \quad \lambda \in {{\mathbb C}\setminus {\mathbb R}}, \end{aligned}$$

where the second identity holds by Lemma 3.4 (d). Hence ${{\widehat{\gamma }} }(\lambda )$ is an operator from ${\text {dom }}Z(\lambda )$ onto ${{\widehat{\mathfrak N}} }_\lambda ({\mathcal {T}})$. Therefore, one can express the action of Γ on the subspace $\widehat{\mathfrak N}_\lambda ({\mathcal {T}})$, $\lambda \in {{\mathbb C}\setminus {\mathbb R}}$, as follows

3.26 $$\begin{aligned} \Gamma \upharpoonright \widehat{\mathfrak N}_\lambda ({\mathcal {T}}) =\bigl \{ \{\widehat{\gamma }(\lambda )\varphi ,\{\varphi ,\varphi '\}\}: \{\varphi , \varphi '\}\in Z(\lambda ) \bigr \}, \end{aligned}$$

in particular, $\Gamma \widehat{\gamma }(\lambda )=Z(\lambda )$, $\lambda \in {{\mathbb C}\setminus {\mathbb R}}$. The following identity

3.27 $$\begin{aligned} \Gamma _0({{\widehat{\mathfrak N}} }_\lambda ({\mathcal {T}}))={\text {dom }}Z(\lambda ), \quad \lambda \in {{\mathbb C}\setminus {\mathbb R}}, \end{aligned}$$

will turn out to be useful.

#### Definition 3.6

Let *S* be a closed symmetric relation in H and let Γ be a boundary relation from ${\mathfrak H}^2$ to ${\mathcal {G}}^2$ for $S^*$. The γ-field γ corresponding to Γ is given by the family

3.28 $$\begin{aligned} \gamma (\lambda ) := \big \{ \{\varphi , f_\lambda \} \in {\mathcal {G}}\times {\mathfrak H}:\, \{{{\widehat{f}} }_\lambda , {{\widehat{\varphi }} }\} \in \Gamma , \,\, {{\widehat{f}} }_\lambda \in {{\widehat{\mathfrak N}} }_\lambda ({\mathcal {T}}) \big \}, \quad \lambda \in {{\mathbb C}\setminus {\mathbb R}}. \end{aligned}$$

In other words, the γ-field is the first component of the single-valued linear mapping $\widehat{\gamma }(\lambda )=(\Gamma _0\upharpoonright \widehat{\mathfrak N}_\lambda ({\mathcal {T}}))^{-1}$ from ${\text {dom }}Z(\lambda )$ onto $\widehat{\mathfrak N}_\lambda ({\mathcal {T}})$, $\lambda \in {{\mathbb C}\setminus {\mathbb R}}$; see (3.23). Observe that ${\text {dom }}\gamma (\lambda )={\text {dom }}Z(\lambda )$ (see (3.24)) and

3.29 $$\begin{aligned} \ker \gamma (\lambda ) =\mathrm{mul\,}\Gamma _0 \quad \text{ and } \quad \mathrm{mul\,}\gamma ({\lambda })^* =\mathrm{mul\,}Z(\bar{\lambda }), \quad \lambda \in {{\mathbb C}\setminus {\mathbb R}}. \end{aligned}$$

For the first identity see (3.25); furthermore, this space does not need to be closed; see [11, Propositions 5.2, 5.3]. For the proof of the second identity use $\mathrm{mul\,}\gamma ({\lambda })^* =({\text {dom }}\gamma ({\lambda }) )^\perp$ and Definitions 3.1, 3.3, and 3.6. Also $\mathrm{mul\,}\gamma ({\lambda })^*$ does not depend on $\lambda \in {{\mathbb C}\setminus {\mathbb R}}$; see Lemma 3.4 (c).

Let *S* be a closed symmetric relation in H and let Γ be a boundary relation for $S^*$. A frequently used notion is the so-called *transposed boundary relation*

3.30 $$\begin{aligned} \Gamma ^\top := \big \{ \{{{\widehat{f}} }, \{\varphi ', -\varphi \} \} :\, \{{{\widehat{f}} }, {{\widehat{\varphi }} }\} \in \Gamma \big \}, \end{aligned}$$

which is indeed a boundary relation. The corresponding γ-field will be denoted by $\gamma ^\top$, so that

3.31 $$\begin{aligned} \gamma ^\top (\lambda )= \big \{ \{\varphi ', \gamma (\lambda ) \varphi \} :\, \{\varphi , \varphi ' \} \in Z(\lambda ) \big \}, \quad \lambda \in {{\mathbb C}\setminus {\mathbb R}}, \end{aligned}$$

and the corresponding Weyl family will be denoted by $Z^\top$, so that

3.32 $$\begin{aligned} Z^\top (\lambda )=\big \{ \{\varphi ', -\varphi \} :\, \{{{\widehat{f}} }, {{\widehat{\varphi }} }\} \in \Gamma , \,\, {{\widehat{f}} }_{\lambda } \in {{\widehat{\mathfrak N}} }_{\lambda }({\mathcal {T}}) \big \} =-Z(\lambda )^{-1}, \quad \lambda \in {{\mathbb C}\setminus {\mathbb R}}. \end{aligned}$$

Remark 3.7 below describes a general way to transform boundary relations.

#### Remark 3.7

It will be useful to interpret the Green’s identity (3.1) in terms of indefinite inner product spaces. Note that the Hilbert space ${\mathfrak H}^2$ and ${\mathcal {G}}^2$ can be seen as Kreĭn spaces $({\mathfrak H}^2,J_{\mathfrak H})$ and $({\mathcal {G}}^2,J_{\mathcal {G}})$, whose inner products $[\cdot . \cdot ]_{{\mathfrak H}^2}$ and $[\cdot . \cdot ]_{{\mathcal {G}}^2}$ are given by the fundamental symmetries

$$ J_{\mathfrak H}=\begin{pmatrix} 0 &  -iI_{\mathfrak H}\\ i I_{\mathfrak H}&  0 \end{pmatrix} \quad \text{ and } \quad J_{\mathcal {G}}=\begin{pmatrix} 0 &  -iI_{\mathcal {G}}\\ i I_{\mathcal {G}}&  0 \end{pmatrix}, $$

respectively, so that

$$ [{{\widehat{f}} }, {{\widehat{g}} }\,]_{{\mathfrak H}^2}=(J_{\mathfrak H}{{\widehat{f}} }, {{\widehat{g}} }\,)_{{\mathfrak H}^2}, \,\, {{\widehat{f}} }, {{\widehat{g}} } \in {\mathfrak H}^2 \quad \text{ and } \quad [{{\widehat{\varphi }} }, {{\widehat{\psi }} }\,]_{{\mathcal {G}}^2}=(J_{\mathcal {G}}{{\widehat{\varphi }} }, {{\widehat{\psi }} }\,)_{{\mathcal {G}}^2}, \,\, {{\widehat{\varphi }} }, {{\widehat{\psi }} }\in {\mathcal {G}}^2. $$

Any relation Γ from $({\mathfrak H}^2,J_{\mathfrak H})$ to $({\mathcal {G}}^2,J_{\mathcal {G}})$ satisfying (D2) is *isometric* in the sense of Kreĭn spaces, i.e., $\Gamma ^{-1} \subset \Gamma ^{[*]}$, where $\Gamma ^{[*]}=J_{\mathfrak H}\Gamma ^*J_{\mathcal {G}}$; cf. (3.2). Moreover, the condition (D3) means that $\Gamma ^{[*]} \subset \Gamma ^{-1}$, and thus the conditions (D2) and (D3) together mean that Γ is *unitary* in the sense of Kreĭn spaces, i.e., $\Gamma ^{-1}=\Gamma ^{[*]}$; see [14, Proposition 3.2]. In particular, if Γ is unitary, then Γ is automatically closed, in which case (3.4) and (3.5) hold. Recall, that if Γ is isometric and satisfies $\mathrm{\overline{dom}\,}\Gamma =S^*$ and ${\text {ran }}\Gamma ={\mathcal {G}}^2$, then Γ is unitary. In fact, these conditions imply that Γ is necessarily an ordinary boundary triplet, see Definition 6.5 below; cf. [14, Proposition 2.5].

At one stage one needs the *Shmul’yan transform*, first introduced in [35], in the following setting. Let *W* be a *standard unitary operator* in the Kreĭn space $({\mathcal {G}}^2, J_{\mathcal {G}})$, which means that $W^{[*]}=W^{-1}$ and $W\in {\textbf{B}}({\mathcal {G}}^2)$. Here $W^{[*]}=J_{\mathcal {G}}W^*J_{\mathcal {G}}$ and hence ${\text {ran }}W={\text {dom }}W^{[*]}={\mathcal {G}}^2$ and $\ker W=\mathrm{mul\,}W^{[*]}=\{0\}$, since ${\text {dom }}W^*={\mathcal {G}}^2$ and $\mathrm{mul\,}W^*=({\text {dom }}W)^\perp =\{0\}$. In particular, $W^{-1} \in {\textbf{B}}({\mathcal {G}}^2)$ is also a standard unitary operator in $({\mathcal {G}}^2, J_{\mathcal {G}})$. For any linear relation Θ in ${\mathcal {G}}$ one defines the Shmul’yan transform W[Θ] as the linear relation in ${\mathcal {G}}^2$ by

$$ W[\Theta ]=\big \{ W{{\widehat{\varphi }} }:\,\, {{\widehat{\varphi }} }\in \Theta \big \} =\big \{ \{ W_{00} \varphi +W_{01} \varphi ', W_{10} \varphi +W_{11} \varphi ' \}:\,\, {{\widehat{\varphi }} }\in \Theta \big \}, $$

where $W=(W_{ij})$, i,j=0,1, is decomposed according to ${\mathcal {G}}^2={\mathcal {G}}\oplus {\mathcal {G}}$; see [35]. For any relation Θ in ${\mathcal {G}}$ one sees that $W[\Theta ]^*=W[\Theta ^*]$; moreover, this transform preserves notions like (maximal) symmetry, (maximal) dissipativity, (maximal) accumulativity and self-adjointness; see [15, Proposition 3.10]. Likewise, if Θ is a unitary relation in ${\mathcal {G}}$, then W[Θ] is a unitary relation in ${\mathcal {G}}$; cf. [15, Theorem 2.10].

Let *S* be a closed symmetric relation in H and let Γ be a boundary relation from ${\mathfrak H}^2$ to ${\mathcal {G}}^2$ for $S^*$. Let γ and *Z* be the corresponding γ-field and Weyl family. Assume that *W* is a standard unitary operator in the Kreĭn space $({\mathcal {G}}^2, J_{\mathcal {G}})$. Then

3.33 $$\begin{aligned} W\circ \Gamma =\big \{\{{{\widehat{f}} }, \{W_{00} \varphi + W_{01} \varphi ', W_{10} \varphi + W_{11}\varphi ' \}\}:\, \{{{\widehat{f}} }, {{\widehat{\varphi }} }\} \in \Gamma \big \} \end{aligned}$$

is also a boundary relation for $S^*$ (with the same domain and kernel as Γ), whose γ-field is given by

3.34 $$\begin{aligned} \gamma _W (\lambda ) = \big \{ \{ W_{00} \varphi + W_{01} \varphi ', \gamma (\lambda ) \varphi \} :\, \{\varphi , \varphi ' \} \in Z(\lambda ) \big \}, \,\, \lambda \in {{\mathbb C}\setminus {\mathbb R}}, \end{aligned}$$

and whose Weyl family is given by

3.35 $$\begin{aligned} Z_W (\lambda ) = \big \{ \{W_{00} \varphi + W_{01} \varphi ', W_{10} \varphi + W_{11}\varphi ' \} :\, \{\varphi , \varphi ' \} \in Z(\lambda ) \big \}, \,\, \lambda \in {{\mathbb C}\setminus {\mathbb R}},\nonumber \\ \end{aligned}$$

see [15, Proposition 3.11]. As a particular case one may take W=J, where

$$ J= \begin{pmatrix} 0 &  I_{\mathcal {G}}\\ -I_{\mathcal {G}}&  0 \end{pmatrix}, $$

then one sees that $J \circ \Gamma =\Gamma ^\top$ turns (3.33), (3.34), and (3.35) into the corresponding facts for the transposed boundary relation (3.30), (3.31), and (3.32); see [14, Section 3.1], [15, Remark 3.12], and Sect. 8.

### Boundary Relations and their Main Transform

Before stating the fundamental result for boundary relations, some facts concerning closed symmetric relations will be recalled. Let *S* be a closed symmetric relation in a Hilbert space H and define the symmetric relations $S_{\textrm{sim}}$ and $S_{\textrm{sa}}$ by

$$ S_{\textrm{sim}}=S \cap {\mathfrak H}(S_{\textrm{sim}})^2 \quad \text{ and } \quad S_{\textrm{sa}}=S \cap {\mathfrak H}(S_{\textrm{sa}})^2, $$

where the closed linear subspaces ${\mathfrak H}(S_{\textrm{sim}})$ and ${\mathfrak H}(S_{\textrm{sa}})$ are given by

$$ {\mathfrak H}(S_{\textrm{sim}}) = \overline{\textrm{span}} \,\big \{ {\mathfrak N}_\lambda (S^*):\, \lambda \in {{\mathbb C}\setminus {\mathbb R}}\big \} \quad \text{ and } \quad {\mathfrak H}(S_{\textrm{sa}}) = \bigcap _{\lambda \in {{\mathbb C}\setminus {\mathbb R}}} {\text {ran }}(S-\lambda ), $$

respectively. Then there is an orthogonal decomposition of the Hilbert space H and a corresponding orthogonal decomposition of the closed symmetric relation *S*, given by

3.36 $$\begin{aligned} {\mathfrak H}={\mathfrak H}(S_{\textrm{sim}}) \oplus {\mathfrak H}(S_{\textrm{sa}}) \quad \text{ and } \quad S=S_{\textrm{sim}} {{\,\mathrm{\, \widehat{\oplus }\,}\,}}S_{\textrm{sa}}. \end{aligned}$$

Here $S_{\textrm{sim}}$ is a simple (completely nonself-adjoint) closed symmetric operator in ${\mathfrak H}(S_{\textrm{sim}})$ and $S_{\textrm{sa}}$ is a self-adjoint relation in ${\mathfrak H}(S_{\textrm{sa}})$; see [5, Theorem 3.4.4].

The formulation of the following theorem is based on a unique self-adjoint relation ${{\widetilde{H}} }$ that is connected with the boundary relation Γ. The relevant mapping $\Gamma \mapsto {{\widetilde{H}} }$ will be called the *main transform*, going back to [14]. In the terminology of Remark 3.7 the main transform takes the boundary relation Γ, which is unitary in the sense of Kreĭn spaces, into a relation ${{\widetilde{H}} }$, which is self-adjoint in the sense of Hilbert spaces. The vector notation in the theorem is self-explanatory. Note that in (3.40) the operator $P_{\mathfrak H}\bigl (\widetilde{H}-\lambda \bigr )^{-1}$ is applied to the set $\mathcal {G}$; cf. (3.9).

#### Theorem 3.8

Let *S* be a closed symmetric relation in H and let Γ be a boundary relation from ${\mathfrak H}^2$ to ${\mathcal {G}}^2$ for $S^*$ as in Definition 3.1. Then the relation ${{\widetilde{H}} }$ in ${\mathfrak H}\oplus {\mathcal {G}}$ defined by

3.37 $$\begin{aligned} \widetilde{H}:=\left\{ \left\{ \begin{pmatrix} f \\ \varphi \end{pmatrix}, \begin{pmatrix}f ^\prime \\ varphi^\prime \end{pmatrix}\right\} :\, \left\{ \begin{pmatrix}f \\ f^\prime \end{pmatrix}, \begin{pmatrix} \varphi \\ \varphi ^\prime \end{pmatrix} \right\} \in \Gamma \right\} \subset \begin{pmatrix} {\mathfrak H}\\ {\mathcal {G}}\end{pmatrix} \times \begin{pmatrix} {\mathfrak H}\\ {\mathcal {G}}\end{pmatrix} \end{aligned}$$

is a self-adjoint extension of $S={{\widetilde{H}} } \cap {\mathfrak H}^2$, whose resolvent is given by

3.38 $$\begin{aligned} (\widetilde{H} -\lambda )^{-1} =\left\{ \left\{ \begin{pmatrix}f ^\prime -\lambda f \\ varphi^\prime -\lambda \varphi \end{pmatrix}, \begin{pmatrix} f \\ \varphi \end{pmatrix} \right\} :\, \left\{ \begin{pmatrix}f \\ f^\prime \end{pmatrix}, \begin{pmatrix} \varphi \\ \varphi ^\prime \end{pmatrix} \right\} \in \Gamma \right\} . \end{aligned}$$

The compression of the resolvent $({{\widetilde{H}} }-\lambda )^{-1}$ to ${\mathcal {G}}$ satisfies

3.39 $$\begin{aligned} P_{\mathcal {G}}\bigl (\widetilde{H}-\lambda \bigr )^{-1}\upharpoonright {\mathcal {G}}=-\bigl (Z(\lambda )+\lambda \bigr )^{-1},\quad \lambda \in {\mathbb C}\setminus {\mathbb R}, \end{aligned}$$

where $P_{\mathcal {G}}$ is the orthogonal projection from ${\mathfrak H}\oplus {\mathcal {G}}$ onto ${\mathcal {G}}$, and the Weyl family Z(λ), $\lambda \in {{\mathbb C}\setminus {\mathbb R}}$, is a Nevanlinna family. Moreover,

3.40 $$\begin{aligned} {\text {ran }}({\mathcal {T}}-\lambda )={\mathfrak H}\quad \text{ and } \quad P_{\mathfrak H}\bigl (\widetilde{H}-\lambda \bigr )^{-1}{\mathcal {G}}={\mathfrak N}_\lambda ({\mathcal {T}}), \quad \lambda \in {{\mathbb C}\setminus {\mathbb R}}, \end{aligned}$$

where $P_{\mathfrak H}$ the orthogonal projection from ${\mathfrak H}\oplus {\mathcal {G}}$ onto H. Finally, there is the orthogonal decomposition

3.41 $$\begin{aligned} {\mathfrak H}\oplus {\mathcal {G}}=\overline{\textrm{span}} \, \bigl \{{\mathcal {G}},\, ({{\widetilde{H}} }-\lambda )^{-1}{\mathcal {G}}:\, \lambda \in {{\mathbb C}\setminus {\mathbb R}}\bigr \} \oplus {\mathfrak H}(S_{\textrm{sa}}). \end{aligned}$$

Consequently, the self-adjoint relation ${{\widetilde{H}} }$ in (3.37) satisfies

3.42 $$\begin{aligned} {\mathfrak H}\oplus {\mathcal {G}}=\overline{\textrm{span}} \, \bigl \{{\mathcal {G}},\, ({{\widetilde{H}} }-\lambda )^{-1}{\mathcal {G}}:\, \lambda \in {{\mathbb C}\setminus {\mathbb R}}\bigr \} \end{aligned}$$

if and only if *S* is a simple symmetric operator in H.

#### Proof

Let *S* be a closed symmetric relation in H and let Γ be a boundary relation for $S^*$. Then it follows from (3.6) and (3.37) that

$$ S=\ker \Gamma ={{\widetilde{H}} } \cap {\mathfrak H}^2, $$

so that the relation ${{\widetilde{H}} }$ is an extension of *S*. Moreover, the relation ${{\widetilde{H}} }$ is symmetric due to Green’s identity in (D2), and it is self-adjoint due to the maximality condition in (D3). It is a straightforward consequence of (3.37) that the resolvent operator $({{\widetilde{H}} } -\lambda )^{-1}$, $\lambda \in \rho ({{\widetilde{H}} })$, is given by (3.38). At this point recall that

3.43 $$\begin{aligned} {\mathcal {T}}={\text {dom }}\Gamma =\left\{ \{f,f'\} :\, \left\{ \begin{pmatrix}f \\ \varphi \end{pmatrix}, \begin{pmatrix} f' \\ -\varphi ^\prime \end{pmatrix} \right\} \in {{\widetilde{H}} } \right\} . \end{aligned}$$

Hence, by restricting to elements $f^{\prime }=\lambda f$ in (3.38), one obtains

3.44 $$\begin{aligned} (\widetilde{H} -\lambda )^{-1} {\upharpoonright \,}{\mathcal {G}}=\left\{ \left\{ \begin{pmatrix}0\\ varphi^\prime -\lambda \varphi \end{pmatrix}, \begin{pmatrix} f \\ \varphi \end{pmatrix} \right\} :\, \left\{ \begin{pmatrix}f \\ \lambda f \end{pmatrix}, \begin{pmatrix} \varphi \\ \varphi ^\prime \end{pmatrix} \right\} \in \Gamma \right\} , \end{aligned}$$

which then leads to (3.39):

3.45 $$\begin{aligned} P_{\mathcal {G}}(\widetilde{H} -\lambda )^{-1} {\upharpoonright \,}{\mathcal {G}}=\big \{ \{-\varphi ' -\lambda \varphi , \varphi \} : \{\varphi , \varphi '\} \in Z(\lambda ) \big \}, \end{aligned}$$

since the right-hand side equals $-(Z(\lambda )+\lambda )^{-1}$.

To see that Z(λ), $\lambda \in {{\mathbb C}\setminus {\mathbb R}}$, is a Nevanlinna family, let $\widehat{\varphi }\in Z(\lambda )$. Then by (3.23) there exists ${{\widehat{f}} }_{\lambda } \in {{\widehat{\mathfrak N}} }_{\lambda }({\mathcal {T}})$, such that $\{{{\widehat{f}} }_{\lambda },{{\widehat{\varphi }} }\}\in \Gamma$, which by (3.16) leads to

$$ \mathrm{Im\,}(\varphi ', \varphi )_{\mathcal {G}}=(\mathrm{Im\,}\lambda ) \, (f_\lambda , f_\lambda )_{\mathfrak H}. $$

Thus Z(λ) is dissipative (accumulative) for $\lambda \in {\mathbb C}^+$ (${\mathbb C}^-$). Moreover, (3.39) now shows that the family *Z* satisfies (F1), (F2), and ($\text {F3}^\prime$). Thus Z(λ), $\lambda \in {{\mathbb C}\setminus {\mathbb R}}$, is a Nevanlinna family.

Now the remaining statements will be proved. The first assertion in (3.40) is clear from (3.38) since ${{\widetilde{H}} }$ is self-adjoint. The second assertion follows from (3.44) and the definition of ${\mathfrak N}_\lambda ({\mathcal {T}})$; cf. (3.14). Recall that ${\mathfrak N}_\lambda ({\mathcal {T}})$ is dense in ${\mathfrak N}_\lambda (S^*)$ and hence one concludes from (3.40) that for all $\lambda \in {{\mathbb C}\setminus {\mathbb R}}$,

$$\begin{aligned} \overline{\textrm{span}} \, \bigl \{P_{\mathfrak H}({{\widetilde{H}} }-\lambda )^{-1}{\mathcal {G}}:\,\lambda \in {{\mathbb C}\setminus {\mathbb R}}\bigr \}&=\overline{\textrm{span}} \, \bigl \{{\mathfrak N}_\lambda ({\mathcal {T}}):\,\lambda \in {{\mathbb C}\setminus {\mathbb R}}\bigr \} \\&=\overline{\textrm{span}} \, \bigl \{{\mathfrak N}_\lambda (S^*):\,\lambda \in {{\mathbb C}\setminus {\mathbb R}}\bigr \} \\&={\mathfrak H}(S_{\textrm{sim}}) \end{aligned}$$

and, since

$$ \overline{\textrm{span}} \, \bigl \{{\mathcal {G}},\, ({{\widetilde{H}} }-\lambda )^{-1}{\mathcal {G}}:\, \lambda \in {{\mathbb C}\setminus {\mathbb R}}\bigr \}={\mathcal {G}}\oplus \overline{\textrm{span}} \, \bigl \{P_{\mathfrak H}({{\widetilde{H}} }-\lambda )^{-1}{\mathcal {G}}:\,\lambda \in {{\mathbb C}\setminus {\mathbb R}}\bigr \}, $$

the decomposition in (3.41) follows now from (3.36). The statement in (3.42) is clear from the decomposition (3.41). □

#### Definition 3.9

The boundary relation Γ from ${\mathfrak H}^2$ to ${\mathcal {G}}^2$ in Definition 3.1 is said to be *minimal* if one of the following equivalent conditions is satisfied:

(i) ${\mathfrak H}\oplus {\mathcal {G}}=\overline{\textrm{span}} \, \bigl \{{\mathcal {G}},\, ({{\widetilde{H}} }-\lambda )^{-1}{\mathcal {G}}:\, \lambda \in {{\mathbb C}\setminus {\mathbb R}}\bigr \}$, that is, ${{\widetilde{H}} }$ is minimal with respect to ${\mathcal {G}}$;
(ii) $${\mathfrak H}=\overline{\textrm{span}} \, \bigl \{P_{\mathfrak H}({{\widetilde{H}} }-\lambda )^{-1}{\mathcal {G}}:\, \lambda \in {{\mathbb C}\setminus {\mathbb R}}\bigr \}= \overline{\textrm{span}} \,\big \{ {\mathfrak N}_\lambda ({\mathcal {T}}):\, \lambda \in {{\mathbb C}\setminus {\mathbb R}}\big \}$$;
(iii) *S* is a closed simple symmetric operator in H.

It follows from Theorem 3.8 and Definition 3.9 that minimal boundary relations for $S^*$ with the same Weyl function are unitarily equivalent; see [5, Theorem 4.2.3].

The following straightforward, but useful, observation is based on the inversion of the main transform.

#### Corollary 3.10

Let ${{\widetilde{H}} }$ be a self-adjoint relation in ${\mathfrak H}\oplus {\mathcal {G}}$ and let the closed symmetric relation *S* in H be given by

$$ S=\ker \Gamma =\widetilde{H} \cap {\mathfrak H}^2. $$

Then the linear relation Γ from ${\mathfrak H}^2$ to ${\mathcal {G}}^2$, defined by

$$ \Gamma = \left\{ \left\{ \begin{pmatrix}f \\ f^\prime \end{pmatrix}, \begin{pmatrix} \varphi \\ \varphi ^\prime \end{pmatrix} \right\} :\, \left\{ \begin{pmatrix} f \\ \varphi \end{pmatrix}, \begin{pmatrix} f ^\prime \\ varphi^\prime \end{pmatrix} \right\} \in {{\widetilde{H}} }\right\} , $$

is a boundary relation for $S^*$. In particular, Γ is minimal if and only if *S* is a simple operator in H.

As a consequence of this corollary it follows that for any closed symmetric relation *S* in H (whose defect numbers are not necessarily equal) there exists a boundary relation as in Definition 3.1. Just consider any self-adjoint exit space extension ${{\widetilde{H}} }$ of *S* and take the inverse main transform. For a particular construction of such a self-adjoint extension (allowing unequal defect numbers for *S*), see [5, Proposition 3.7].

The discussion about the orthogonal decompositions in (3.36) will now be continued. Let ${{\widetilde{H}} }$ be a self-adjoint relation in ${\mathfrak H}\oplus {\mathcal {G}}$ and assume that it is an extension of *S* in H. Then ${\mathfrak H}\oplus {\mathcal {G}}$ has the orthogonal decomposition

$$ {\mathfrak H}\oplus {\mathcal {G}}= ({\mathfrak H}(S_\textrm{sim}) \oplus {\mathcal {G}}) \oplus {\mathfrak H}(S_\textrm{sa}), $$

and ${{\widetilde{H}} }$ has the corresponding orthogonal decomposition

3.46 $$\begin{aligned} {{\widetilde{H}} }= {{\widetilde{H}} }_\textrm{sim} {{\,\mathrm{\, \widehat{\oplus }\,}\,}}S_\textrm{sa}. \end{aligned}$$

In this decomposition ${{\widetilde{H}} }_\textrm{sim}$ is a self-adjoint extension of $S_\textrm{sim}$ in ${\mathfrak H}(S_\textrm{sim}) \oplus {\mathcal {G}}$ and $S_\textrm{sa}$ is a self-adjoint relation in ${\mathfrak H}(S_\textrm{sa}) \subset {\mathfrak H}$. In the context of Theorem 3.8 it follows from (3.43) that for the boundary relation Γ one has

$$ {\mathcal {T}}={\mathcal {T}}_\textrm{sim} {{\,\mathrm{\, \widehat{\oplus }\,}\,}}S_\textrm{sa}, \quad {\mathfrak N}_\lambda (S_\textrm{sa})=\{0\}, \quad {\mathfrak N}_\lambda ({\mathcal {T}})={\mathfrak N}_\lambda ({\mathcal {T}}_\textrm{sim}), \quad \lambda \in {{\mathbb C}\setminus {\mathbb R}}. $$

where now ${\mathcal {T}}_\textrm{sim}$ is the smooth version of $(S_\textrm{sim})^*$ in the Hilbert space ${\mathfrak H}(S_\textrm{sim})$, in other words

3.47 $$\begin{aligned} {\mathcal {T}}_\textrm{sim} =\left\{ \{h,h'\} \in {\mathfrak H}(S_\textrm{sim}) :\, \left\{ \begin{pmatrix} h \\ \varphi \end{pmatrix}, \begin{pmatrix} h' \\ -\varphi ^\prime \end{pmatrix} \right\} \in {{\widetilde{H}} }_\textrm{sim} \right\} . \end{aligned}$$

It is clear that the inverse main transform of the self-adjoint relation ${{\widetilde{H}} }_\textrm{sim}$ in ${\mathfrak H}(S_\textrm{sim}) \oplus {\mathcal {G}}$ leads to a minimal boundary relation from ${\mathfrak H}(S_\textrm{sim})^2$ to ${\mathcal {G}}^2$ for $(S_\textrm{sim})^*$ with the same Weyl family as the one that belongs to the original boundary relation Γ from ${\mathfrak H}^2$ to ${\mathcal {G}}^2$ for $S^*$.

### Description of the γ-Field and its Adjoint

The γ-field $\gamma (\lambda )$, $\lambda \in {{\mathbb C}\setminus {\mathbb R}}$, of a boundary relation Γ from ${\mathfrak H}^2$ to ${\mathcal {G}}^2$ is a vector-valued function from ${\mathcal {G}}$ into H, which maps ${\text {dom }}\gamma (\lambda )={\text {dom }}Z(\lambda )$ onto ${\mathfrak N}_\lambda ({\mathcal {T}})$; see Definition 3.6. Recall that $\mathrm{mul\,}\gamma (\lambda )^*=\mathrm{mul\,}Z(\bar{\lambda })$, $\lambda \in {{\mathbb C}\setminus {\mathbb R}}$; cf. (3.29). The next lemma describes the γ-field and its adjoint $\gamma (\lambda )^*$ by means of the main transform ${{\widetilde{H}} }$ of Γ.

#### Lemma 3.11

Let Γ be a boundary relation from ${\mathfrak H}^2$ to ${\mathcal {G}}^2$ for $S^*$ with the Weyl family Z(λ) and the γ-field $\gamma (\lambda )$, $\lambda \in {{\mathbb C}\setminus {\mathbb R}}$, and let ${{\widetilde{H}} }$ be the main transform of Γ. Then the γ-field is an operator from ${\mathcal {G}}$ to H, given by

3.48 $$\begin{aligned} \gamma (\lambda )=-\big ( P_{\mathfrak H}(\widetilde{H} -\lambda )^{-1}\upharpoonright {\mathcal {G}}\big )\, (Z(\lambda ) +\lambda ),\quad \lambda \in {{\mathbb C}\setminus {\mathbb R}}. \end{aligned}$$

Moreover, the adjoint of the γ-field is a closed relation from H to ${\mathcal {G}}$, given by

3.49 $$\begin{aligned} \gamma (\lambda )^* = -(Z(\bar{\lambda })+ \bar{\lambda }) \,P_{\mathcal {G}}({{\widetilde{H}} } -\bar{\lambda })^{-1}\upharpoonright {\mathfrak H}, \quad \lambda \in {{\mathbb C}\setminus {\mathbb R}}. \end{aligned}$$

#### Proof

First note that the resolvent of the main transform ${{\widetilde{H}} }$ of Γ in (3.38) leads to the operator

3.50 $$\begin{aligned} P_{\mathfrak H}({{\widetilde{H}} }-\lambda )^{-1} {\upharpoonright \,}{\mathcal {G}}=\big \{ \{-\varphi '-\lambda \varphi , \gamma (\lambda ) \varphi \} :\, \{\varphi , \varphi '\} \in Z(\lambda ) \big \} \in {\textbf{B}}({\mathcal {G}}, {\mathfrak H}). \end{aligned}$$

Hence the domain of definition of the right-hand side of (3.48) is ${\text {dom }}Z(\lambda )$, which is equal to ${\text {dom }}\gamma (\lambda )$. To see the equality (3.48), let $\{\varphi , \eta \}$ be an element in the right-hand side of (3.48). Then there exists $\varphi ' \in {\mathcal {G}}$ such that $\{\varphi , \varphi '\} \in Z(\lambda )$ and

$$ \{\varphi , -\varphi ' -\lambda \varphi \} \in -(Z(\lambda )+\lambda ) \quad \text{ and } \quad \{-\varphi ' -\lambda \varphi , \eta \} \in P_{\mathfrak H}({{\widetilde{H}} }-\lambda )^{-1} {\upharpoonright \,}{\mathcal {G}}. $$

Hence, it follows from (3.50) that $\eta =\gamma (\lambda ) \varphi$. Thus, equality in (3.48) follows.

The compressed resolvent $P_{\mathfrak H}(\widetilde{H} -\lambda )^{-1}\upharpoonright {\mathcal {G}}$ in (3.48) belongs to $\textbf{B}({\mathcal {G}},{\mathfrak H})$ with adjoint $P_{\mathcal {G}}(\widetilde{H} -\bar{\lambda })^{-1}\upharpoonright {\mathfrak H}\in \textbf{B}({\mathfrak H},{\mathcal {G}})$. Thus (3.49) follows by taking adjoints in (3.48); see, e.g. [5, Proposition 1.3.9]. □

Lemma 3.11 gives rise to a useful connection between the adjoint of the γ-field γ and the symmetric extension $S_0$ of *S* in (3.20); cf. [5, Proposition 2.3.2].

#### Proposition 3.12

Let Γ be a boundary relation from ${\mathfrak H}^2$ to ${\mathcal {G}}^2$ for $S^*$ with γ-field $\gamma (\lambda )$, $\lambda \in {{\mathbb C}\setminus {\mathbb R}}$, and let $S_0 = \ker \Gamma _0$. Then for $\lambda \in {{\mathbb C}\setminus {\mathbb R}}$ and $h \in {\text {ran }}(S_0-\bar{\lambda })$ there exists $\eta \in {\mathcal {G}}$ such that

3.51 $$\begin{aligned} \big \{ \{(S_0-\bar{\lambda })^{-1} h, (I+\bar{\lambda }(S_0-\bar{\lambda })^{-1}) h \}, \{0,\eta \} \big \} \in \Gamma ; \end{aligned}$$

moreover, for any $\eta \in {\mathcal {G}}$ for which (3.51) holds one has $\{h, \eta \} \in \gamma (\lambda )^*$. Conversely, if $\{h, \eta \} \in \gamma (\lambda )^*$, then $h \in {\text {ran }}(S_0-\bar{\lambda })$ and (3.51) holds. Consequently,

3.52 $$\begin{aligned}  &   {\text {ran }}(S_0-\bar{\lambda })={\text {dom }}\gamma (\lambda )^*, \nonumber \\  &   {\text {ran }}(S-\bar{\lambda })=({\mathfrak N}_{\lambda }(S^*))^\perp =\ker \gamma (\lambda )^*, \quad \lambda \in {{\mathbb C}\setminus {\mathbb R}}. \end{aligned}$$

#### Proof

First observe that the resolvent of the main transform ${{\widetilde{H}} }$ of Γ in (3.38) leads to

3.53 $$\begin{aligned} P_{\mathcal {G}}({{\widetilde{H}} } -\bar{\lambda })^{-1}\upharpoonright {\mathfrak H}=\left\{ \left\{ f^\prime - \bar{\lambda }f, \varphi \right\} :\, \left\{ \begin{pmatrix}f \\ f^\prime \end{pmatrix}, \begin{pmatrix} \varphi \\ -\bar{\lambda } \varphi \end{pmatrix} \right\} \in \Gamma \right\} . \end{aligned}$$

Let $h \in {\text {ran }}(S_0-\bar{\lambda })$. Then there exists $\{f,f'\}\in S_0$ with $f=(S_0-\bar{\lambda })^{-1}h$ and $f'=(I+\bar{\lambda }(S_0-\bar{\lambda })^{-1}) h$, so that $h=f'-\bar{\lambda } f$. By assumption there exists an element $\eta \in {\mathcal {G}}$ for which

3.54 $$\begin{aligned} \big \{ \{f,f'\}, \{0, \eta \} \big \} \in \Gamma . \end{aligned}$$

Now let $\eta \in {\mathcal {G}}$ be any element for which (3.54) holds and choose $\{\varphi , \varphi '\} \in Z(\bar{\lambda })$ such that $\eta =-\varphi '-\bar{\lambda }\varphi$; cf. (F1). Moreover, by (3.17) there exists $\{g, \bar{\lambda } g\} \in {{\widehat{\mathfrak N}} }_{\bar{\lambda }}({\mathcal {T}})$ such that

3.55 $$\begin{aligned} \big \{ \{g, \bar{\lambda } g\}, \{\varphi , \varphi '\} \big \} \in \Gamma . \end{aligned}$$

Then it is clear that $\big \{ \{f+g, f'+\bar{\lambda } g\}, \{\varphi , \varphi '+\eta \} \big \} \in \Gamma$ or, using the main transform:

$$ \left\{ \left( {\begin{array}{c}f'-\bar{\lambda } f\\ 0\end{array}}\right) , \left( {\begin{array}{c}f+g\\ \varphi \end{array}}\right) \right\} \in ({{\widetilde{H}} } -\bar{\lambda })^{-1}. $$

Thus $\{f'-\bar{\lambda } f, \varphi \} \in P_{\mathcal {G}}({{\widetilde{H}} } -\bar{\lambda })^{-1}\upharpoonright {\mathfrak H}$ and since $\{\varphi , \varphi ' +\bar{\lambda } \varphi \} \in (Z(\bar{\lambda })+\bar{\lambda })$, this shows $\{h, \eta \} \in \gamma (\lambda )^*$ by (3.49). In particular, ${\text {ran }}(S_0-\bar{\lambda }) \subset {\text {dom }}\gamma (\lambda )^*$.

Conversely, let $\{h, \eta \} \in \gamma (\lambda )^*$. Then it follows from (3.49) and (3.53) that there exists $\varphi \in {\mathcal {G}}$ with

$$ \{h, \varphi \}= \{f'-\bar{\lambda } f, \varphi \} \in P_{\mathcal {G}}({{\widetilde{H}} } -\bar{\lambda })^{-1}\upharpoonright {\mathfrak H}, $$

and that there exists $\varphi ' \in {\mathcal {G}}$ with $\{\varphi , \varphi '\} \in Z(\bar{\lambda })$, such that

$$ \{\varphi ,\eta \}=\{\varphi , -\varphi '-\bar{\lambda } \varphi \}. $$

On the other hand, (3.53) shows that

3.56 $$\begin{aligned} \big \{ \{f,f'\}, \{\varphi , -\bar{\lambda } \varphi \} \big \} \in \Gamma . \end{aligned}$$

Now combining (3.56) and (3.55), where $\{g, \bar{\lambda } g\} \in {{\widehat{\mathfrak N}} }_{\bar{\lambda }}({\mathcal {T}})$, leads to

3.57 $$\begin{aligned} \big \{ \{f-g,f' -\bar{\lambda } g\}, \{0, -\varphi ' -\bar{\lambda } \varphi \} \big \} \in \Gamma . \end{aligned}$$

Hence $\left\{ f-g, f^\prime - \bar{\lambda }g \right\} \in S_0$, so that $f^\prime - \bar{\lambda }f=f^\prime - \bar{\lambda }g - \bar{\lambda }(f-g) \in {\text {ran }}(S_0-\bar{\lambda })$. Thus ${\text {dom }}\gamma (\lambda )^* \subset {\text {ran }}(S_0-\bar{\lambda })$. Moreover, it is clear that

$$ \left\{ f^\prime - \bar{\lambda }f, f-g \right\} \in (S_0-\bar{\lambda })^{-1} \,\, \text{ and } \,\, \left\{ f^\prime - \bar{\lambda }f, f^\prime - \bar{\lambda }g \right\} \in I+\bar{\lambda }(S_0-\bar{\lambda })^{-1}, $$

so that (3.57) can be written as (3.51).

It follows from the above statements that ${\text {ran }}(S_0 -\bar{\lambda })={\text {dom }}\gamma (\lambda )^*$. Hence the first identity (3.52) is clear. To prove the identities in the second part of (3.52), observe that

$$ ({\text {ran }}(S-\bar{\lambda }))^\perp =\ker (S^*-\lambda ), \quad \lambda \in {{\mathbb C}\setminus {\mathbb R}}. $$

Since ${\mathfrak N}_\lambda ({\mathcal {T}})={\text {ran }}\gamma (\lambda )$ is dense in ${\mathfrak N}_\lambda (S^*)$, cf. (3.23), this gives $({\text {ran }}\gamma (\lambda ))^\perp =\ker \gamma (\lambda )^*$. □

The results in Proposition 3.12 can be expressed in a different way. Introduce the linear mapping $S_0(\lambda )$, $\lambda \in {{\mathbb C}\setminus {\mathbb R}}$, associated with the relation $S_0$, by

3.58 $$\begin{aligned} S_0(\lambda ): h\rightarrow \begin{pmatrix}(S_0-\lambda )^{-1}h\\ h+\lambda (S_0-\lambda )^{-1}h\end{pmatrix}, \quad h\in {\text {ran }}(S_0-\lambda ), \end{aligned}$$

see [5, Proposition 2.3.2]. Then $S_0(\lambda )$ is a bounded operator from ${\text {ran }}(S_0-\lambda )$ onto $S_0$ with bounded inverse for every $\lambda \in {{\mathbb C}\setminus {\mathbb R}}$. As a consequence of Proposition 3.12 the function $S_0(\lambda )$ and the adjoint $\gamma (\lambda )^*$ are connected by

3.59 $$\begin{aligned} \Gamma _1 S_0(\bar{\lambda })=\gamma (\lambda )^*{{\,\mathrm{\, \widehat{+} \,}\,}}(\{0\}\times \mathrm{mul\,}\Gamma _1), \quad \quad \lambda \in {{\mathbb C}\setminus {\mathbb R}}, \end{aligned}$$

and by

3.60 $$\begin{aligned} \Gamma S_0(\bar{\lambda })=\left( {\begin{array}{c}0\\ \gamma (\lambda )^*\end{array}}\right) {{\,\mathrm{\, \widehat{+} \,}\,}}(\{0\}\times \mathrm{mul\,}\Gamma ), \quad \lambda \in {{\mathbb C}\setminus {\mathbb R}}. \end{aligned}$$

Note that the block relation in (3.60) is defined by

$$ \left( {\begin{array}{c}0\\ \gamma (\lambda )^*\end{array}}\right) = \left\{ \left\{ h, \left( {\begin{array}{c} 0 \\ k\end{array}}\right) \right\} :\, \{h, k\} \in \gamma (\lambda )^* \right\} ; $$

cf. [28]. The formulas (3.59) and (3.60) have been derived in [11, Theorem 5.5] by means of an associated operator colligation.

## Nevanlinna Families as Weyl Families

It is clear that the Weyl family in Definition 3.3 is a Nevanlinna family; see Theorem 3.8. Now it will be shown that for every Nevanlinna family *Z* there exists a closed symmetric relation *S* and a boundary relation for $S^*$ such that *Z* is its Weyl family. This assertion was the main result in [14, Theorem 3.9]. Here the argument is via a direct construction based on the reproducing kernel Hilbert space associated with the Nevanlinna family.

Let $\{{\mathcal {A}},{\mathcal {B}}\}$ be a Nevanlinna pair in a Hilbert space ${\mathcal {G}}$. The corresponding *Nevanlinna kernel* on $({{\mathbb C}\setminus {\mathbb R}}) \times ({{\mathbb C}\setminus {\mathbb R}})$ is defined by

4.1 $$\begin{aligned} \textsf{N}_{{\mathcal {A}},{\mathcal {B}}}(\lambda , \mu ) :=\frac{{\mathcal {B}}(\bar{\lambda })^* {\mathcal {A}}(\bar{\mu }) -{\mathcal {A}}(\bar{\lambda })^*{\mathcal {B}}(\bar{\mu })}{\lambda -\bar{\mu }}, \quad \lambda , \mu \in {{\mathbb C}\setminus {\mathbb R}},\quad \lambda \not =\bar{\mu }. \end{aligned}$$

The kernel $\textsf{N}_{{\mathcal {A}},{\mathcal {B}}}(\cdot ,\cdot )$ is nonnegative (see [5, Theorem 4.4.1]), symmetric, and holomorphic, and therefore it induces a *reproducing kernel Hilbert space*, denoted by ${\mathfrak H}(\textsf{N}_{{\mathcal {A}},{\mathcal {B}}})$. This Hilbert space consists of ${\mathcal {G}}$-valued functions which are holomorphic on ${{\mathbb C}\setminus {\mathbb R}}$, see, e.g. [5, Section 4.4]. The following general representation result can be found in [5, Theorem 4.4.2].

### Theorem 4.1

Let *Z* be a Nevanlinna family in ${\mathcal {G}}$ with corresponding Nevanlinna pair $Z=\{{\mathcal {A}},{\mathcal {B}}\}$ and let ${\mathfrak H}(\textsf{N}_{{\mathcal {A}},{\mathcal {B}}})$ be the associated reproducing kernel Hilbert space. Then

4.2 $$\begin{aligned} {{\widetilde{H}} }_{{\mathcal {A}},{\mathcal {B}}}= \left\{ \left\{ \begin{pmatrix} f \\ \varphi \end{pmatrix}, \begin{pmatrix} f' \\ -\varphi ' \end{pmatrix} \right\} :\, \begin{matrix} f,f' \in {\mathfrak H}(\textsf{N}_{{\mathcal {A}},{\mathcal {B}}}), \,\varphi , \varphi ' \in {\mathcal {G}}\\ f'(\xi )-\xi f(\xi ) ={\mathcal {B}}(\bar{\xi })^* \varphi -{\mathcal {A}}(\bar{\xi })^* \varphi ' \end{matrix} \right\} \end{aligned}$$

is a self-adjoint relation in the Hilbert space ${\mathfrak H}(\textsf{N}_{{\mathcal {A}},{\mathcal {B}}}) \oplus {\mathcal {G}}$ and the compression of the resolvent of ${{\widetilde{H}} }_{{\mathcal {A}},{\mathcal {B}}}$ to ${\mathcal {G}}$ satisfies

4.3 $$\begin{aligned} P_{{\mathcal {G}}} ({{\widetilde{H}} }_{{\mathcal {A}},{\mathcal {B}}} -\lambda )^{-1} \upharpoonright {{\mathcal {G}}} =-(Z(\lambda )+\lambda )^{-1}, \quad \lambda \in {{\mathbb C}\setminus {\mathbb R}}, \end{aligned}$$

where $P_{\mathcal {G}}$ is the orthogonal projection from ${\mathfrak H}(\textsf{N}_{{\mathcal {A}},{\mathcal {B}}}) \oplus {\mathcal {G}}$ onto ${\mathcal {G}}$. Furthermore, the self-adjoint relation ${{\widetilde{H}} }_{{\mathcal {A}},{\mathcal {B}}}$ is minimal with respect to ${\mathcal {G}}$, in the sense that

4.4 $$\begin{aligned} {\mathfrak H}(\textsf{N}_{{\mathcal {A}},{\mathcal {B}}}) \oplus {\mathcal {G}}=\overline{\textrm{span}} \, \bigl \{{\mathcal {G}}, ({{\widetilde{H}} }_{{\mathcal {A}},{\mathcal {B}}}-\lambda )^{-1} {\mathcal {G}}:\, \lambda \in {{\mathbb C}\setminus {\mathbb R}}\bigr \}. \end{aligned}$$

If *Z* is a Nevanlinna family with corresponding Nevanlinna pair $\{{\mathcal {A}},{\mathcal {B}}\}$, then the self-adjoint relation ${{\widetilde{H}} }_{{\mathcal {A}},{\mathcal {B}}}$ in Theorem 4.1, which is induced by *Z*, leads to a minimal boundary relation (see Corollary 3.10), whose Weyl family coincides with the given Nevanlinna family. It turns out that the simple symmetric operator $S_{{\mathcal {A}},{\mathcal {B}}}$ associated with the boundary relation is the operator of multiplication by the independent variable in the reproducing kernel Hilbert space ${\mathfrak H}(\textsf{N}_{{\mathcal {A}},{\mathcal {B}}})$; see also [3, 4, 10]. The following result can be seen as a continuation of Theorem 4.1.

### Theorem 4.2

Let *Z* be a Nevanlinna family in ${\mathcal {G}}$ with corresponding Nevanlinna pair $Z=\{{\mathcal {A}},{\mathcal {B}}\}$ and let ${\mathfrak H}(\textsf{N}_{{\mathcal {A}},{\mathcal {B}}})$ be the associated reproducing kernel Hilbert space as in Theorem 4.1. Let ${{\widetilde{H}} }_{{\mathcal {A}},{\mathcal {B}}}$ be the induced self-adjoint relation in ${\mathfrak H}(\textsf{N}_{{\mathcal {A}},{\mathcal {B}}})$ as in (4.2). Then:

- the linear relation
  4.5 $$\begin{aligned} \Gamma _{{\mathcal {A}},{\mathcal {B}}}=\left\{ \,\left\{ \begin{pmatrix} f \\ f' \end{pmatrix}, \begin{pmatrix} \varphi \\ \varphi '\end{pmatrix} \right\} :\,\left\{ \begin{pmatrix} f \\ \varphi \end{pmatrix} \begin{pmatrix}f ^\prime \\ varphi^\prime \end{pmatrix}\right\} \in {{\widetilde{H}} }_{{\mathcal {A}},{\mathcal {B}}} \,\right\} \end{aligned}$$
  is a minimal boundary relation for $(S_{{\mathcal {A}},{\mathcal {B}}})^*$ with $S_{{\mathcal {A}}, {\mathcal {B}}}=\ker \Gamma _{{\mathcal {A}},{\mathcal {B}}}$;
- the closed symmetric operator $S_{{\mathcal {A}},{\mathcal {B}}}$ in ${\mathfrak H}(\textsf{N}_{{\mathcal {A}},{\mathcal {B}}})$ is simple and it is given by
  4.6 $$\begin{aligned} S_{{\mathcal {A}},{\mathcal {B}}}=\bigl \{\,\{f,f'\}\in {\mathfrak H}(\textsf{N}_{{\mathcal {A}},{\mathcal {B}}})^2 :\, f'(\xi )=\xi f(\xi )\,\bigr \}; \end{aligned}$$
- the linear relation ${\mathcal {T}}_{{\mathcal {A}},{\mathcal {B}}}={\text {dom }}\Gamma _{{\mathcal {A}},{\mathcal {B}}}$ in ${\mathfrak H}(\textsf{N}_{{\mathcal {A}},{\mathcal {B}}})$ is dense in $(S_{{\mathcal {A}},{\mathcal {B}}})^*$ and it is given by
  4.7 $$\begin{aligned} {\mathcal {T}}_{{\mathcal {A}},{\mathcal {B}}}=\left\{ \, \{ f, f'\}\in {\mathfrak H}(\textsf{N}_{{\mathcal {A}},{\mathcal {B}}})^2 :\, \begin{matrix} f'(\xi )-\xi f(\xi )={\mathcal {B}}(\bar{\xi })^* \varphi -{\mathcal {A}}(\bar{\xi })^* \varphi ' \\ \varphi , \varphi ' \in {\mathcal {G}}\end{matrix} \,\right\} ; \end{aligned}$$
- the Weyl family corresponding to $\Gamma _{{\mathcal {A}},{\mathcal {B}}}$ is given by the Nevanlinna family *Z* and the γ-field corresponding to $\Gamma _{{\mathcal {A}},{\mathcal {B}}}$ is the family given by
  4.8 $$\begin{aligned} \gamma _{{\mathcal {A}},{\mathcal {B}}}(\lambda )=\big \{ \{{\mathcal {A}}(\lambda ) \eta , - \textsf{N}_{{\mathcal {A}},{\mathcal {B}}}(\cdot , \bar{\lambda }) \eta \}:\, \eta \in {\mathcal {G}}\big \} \subset {\mathcal {G}}\times {\mathfrak H}(\textsf{N}_{{\mathcal {A}},{\mathcal {B}}}). \end{aligned}$$

### Proof

Let $\{{\mathcal {A}},{\mathcal {B}}\}$ be a Nevanlinna pair for the Nevanlinna family *Z*. Let ${{\widetilde{H}} }_{{\mathcal {A}},{\mathcal {B}}}$ be the induced self-adjoint relation in ${\mathfrak H}(\textsf{N}_{{\mathcal {A}},{\mathcal {B}}})$ as in (4.2) in Theorem 4.1 so that ${{\widetilde{H}} }_{{\mathcal {A}},{\mathcal {B}}}$ is minimal with respect to ${\mathcal {G}}$

(a) and (b) By Corollary 3.10 one sees that $\Gamma _{{\mathcal {A}},{\mathcal {B}}}$ in (4.5) is a boundary relation for the closed symmetric relation $S_{{\mathcal {A}},{\mathcal {B}}}=\ker \Gamma _{{\mathcal {A}},{\mathcal {B}}}$. This boundary relation is minimal since ${{\widetilde{H}} }_{{\mathcal {A}},{\mathcal {B}}}$ is minimal with respect to ${\mathcal {G}}$, which implies that $S_{{\mathcal {A}},{\mathcal {B}}}$ is a simple symmetric operator; cf. Definition 3.9. By definition $S_{{\mathcal {A}},{\mathcal {B}}}=\ker \Gamma _{{\mathcal {A}},{\mathcal {B}}}$ satisfies

$$ S_{{\mathcal {A}},{\mathcal {B}}}={{\widetilde{H}} }_{{\mathcal {A}},{\mathcal {B}}} \cap \left( \begin{pmatrix} {\mathfrak H}(\textsf{N}_{{\mathcal {A}},{\mathcal {B}}})\\ \{0\} \end{pmatrix} \times \begin{pmatrix} {\mathfrak H}(\textsf{N}_{{\mathcal {A}},{\mathcal {B}}})\\ \{0\} \end{pmatrix} \right) , $$

when the space ${\mathfrak H}(\textsf{N}_{{\mathcal {A}},{\mathcal {B}}}) \times \{0\}$ is identified with ${\mathfrak H}(\textsf{N}_{{\mathcal {A}},{\mathcal {B}}})$. This, together with (4.2), shows that $S_{{\mathcal {A}},{\mathcal {B}}}$ coincides with the right-hand side of (4.6).

(c) Since $${\mathcal {T}}_{{\mathcal {A}},{\mathcal {B}}}={\text {dom }}\Gamma _{{\mathcal {A}},{\mathcal {B}}}$$ it follows as in (b) that (4.2) implies (4.7). Moreover, $$({\mathcal {T}}_{{\mathcal {A}},{\mathcal {B}}})^{**}=(S_{{\mathcal {A}},{\mathcal {B}}})^*$$ shows that $${\mathcal {T}}_{{\mathcal {A}},{\mathcal {B}}}$$ is dense in $$(S_{{\mathcal {A}},{\mathcal {B}}})^*$$.

(d) Let the Weyl family of $\Gamma _{{\mathcal {A}},{\mathcal {B}}}$ be denoted by W(λ), $\lambda \in {{\mathbb C}\setminus {\mathbb R}}$. Let $\{\varphi , \varphi '\} \in W(\lambda )$, then $\big \{ \{f_\lambda , \lambda f_\lambda \}, \{\varphi , \varphi '\} \big \} \in \Gamma _{{\mathcal {A}},{\mathcal {B}}}$ for some $f_\lambda \in {\mathfrak N}_\lambda ({\mathcal {T}}_{{\mathcal {A}},{\mathcal {B}}})$. Now by (4.2) one sees that

4.9 $$\begin{aligned} (\lambda -\xi )f_\lambda (\xi )= f_\lambda '(\xi )-\xi f_\lambda (\xi ) ={\mathcal {B}}(\bar{\xi })^* \varphi -{\mathcal {A}}(\bar{\xi })^*\varphi '. \end{aligned}$$

In particular, with $\xi =\lambda$ this gives ${\mathcal {B}}(\bar{\lambda })^* \varphi ={\mathcal {A}}(\bar{\lambda })^*\varphi '$, or $\{\varphi , \varphi '\} \in Z(\lambda )$ due to (2.3). Thus it follows that $W(\lambda ) \subset Z(\lambda )$. For the converse inclusion $Z(\lambda ) \subset W(\lambda )$, let $\{\varphi , \varphi '\} \in Z(\lambda )$ and define

4.10 $$\begin{aligned} f_\lambda (\xi )=\frac{{\mathcal {B}}(\bar{\xi })^* \varphi -{\mathcal {A}}(\bar{\xi })^*\varphi '}{\lambda -\xi }, \quad \xi \ne \lambda . \end{aligned}$$

Observe that with $\{\varphi , \varphi '\}=\{{\mathcal {A}}(\lambda ) \eta , {\mathcal {B}}(\lambda ) \eta \}$ for some $\eta \in {\mathcal {G}}$ one sees that

$$ f_\lambda (\xi ) =-\frac{{\mathcal {B}}(\bar{\xi })^* {\mathcal {A}}(\lambda )-{\mathcal {A}}(\bar{\xi })^* {\mathcal {B}}(\lambda )}{\xi -\lambda } \eta =- \textsf{N}_{{\mathcal {A}},{\mathcal {B}}}(\xi , \bar{\lambda }) \eta , \quad \xi \ne \lambda , $$

so that $f_\lambda \in {\mathfrak H}(\textsf{N}_{{\mathcal {A}},{\mathcal {B}}})$. Thus, (4.9) is satisfied, and $\big \{ \{f_\lambda , \lambda f_\lambda \}, \{\varphi , \varphi '\} \big \} \in \Gamma$, so that $Z(\lambda ) \subset W(\lambda )$. Likewise, the γ-field is given by (4.8). □

For the sake of completeness it will be made explicit how the results in Theorem 4.1 and Theorem 4.2 depend on the choice of Nevanlinna pair $\{{\mathcal {A}},{\mathcal {B}}\}$. Therefore, assume that also $\{\mathcal {C},{\mathcal {D}}\}$ is a Nevanlinna pair for *Z*. Then it follows from Proposition 2.3 that

$$ \mathcal {C}(\lambda )={\mathcal {A}}(\lambda ){\mathcal {X}}(\lambda ), \quad {\mathcal {D}}(\lambda )={\mathcal {B}}(\lambda ){\mathcal {X}}(\lambda ), $$

where ${\mathcal {X}}(\lambda )$, $\lambda \in {{\mathbb C}\setminus {\mathbb R}}$, is a bounded and boundedly invertible holomorphic operator function in ${\textbf{B}}({\mathcal {G}})$. Furthermore, the mapping *U* defined by

$$ U: \begin{pmatrix} {\mathfrak H}(\textsf{N}_{{\mathcal {A}},{\mathcal {B}}}) \\ {\mathcal {G}}\end{pmatrix} \rightarrow \begin{pmatrix} {\mathfrak H}(\textsf{N}_{\mathcal {C},{\mathcal {D}}}) \\ {\mathcal {G}}\end{pmatrix}, \quad \quad \begin{pmatrix} f(\xi ) \\ \varphi \end{pmatrix} \mapsto \begin{pmatrix} {\mathcal {X}}(\bar{\xi })^* f(\xi ) \\ \varphi \end{pmatrix}, $$

is unitary by [5, Proposition 4.1.9]. It is now a direct consequence of Theorem 4.1 that the self-adjoint relation ${{\widetilde{H}} }_{{\mathcal {A}},{\mathcal {B}}}$ in ${\mathfrak H}(\textsf{N}_{{\mathcal {A}},{\mathcal {B}}}) \oplus {\mathcal {G}}$ and the analogous self-adjoint relation ${{\widetilde{H}} }_{\mathcal {C},{\mathcal {D}}}$ in ${\mathfrak H}(\textsf{N}_{\mathcal {C},{\mathcal {D}}}) \oplus {\mathcal {G}}$ are unitarily equivalent under the mapping *U*, i.e.,

$$ {{\widetilde{H}} }_{\mathcal {C},{\mathcal {D}}}=\big \{ \{Uh, Uh'\}:\, \{h,h'\} \in {{\widetilde{H}} }_{{\mathcal {A}},{\mathcal {B}}} \big \}, $$

or, equivalently, ${{\widetilde{H}} }_{\mathcal {C},{\mathcal {D}}}=U {{\widetilde{H}} }_{{\mathcal {A}},{\mathcal {B}}}U^*$; cf. [5, Lemma 4.4.4] and [5, Lemma 4.2.3]. Note that as a consequence of Theorem 4.1 the family

$$ (Z(\lambda )+\lambda )^{-1} ={\mathcal {A}}(\lambda ) ({\mathcal {B}}(\lambda )+\lambda {\mathcal {A}}(\lambda ))^{-1} =\mathcal {C}(\lambda ) ({\mathcal {D}}(\lambda )+\lambda \mathcal {C}(\lambda ))^{-1}, \quad \lambda \in {{\mathbb C}\setminus {\mathbb R}}, $$

can be interpreted as the compression of the resolvent of the self-adjoint relation ${{\widetilde{H}} }_{{\mathcal {A}},{\mathcal {B}}}$ in ${\mathfrak H}(\textsf{N}_{{\mathcal {A}},{\mathcal {B}}}) \oplus {\mathcal {G}}$ and of the resolvent of the self-adjoint relation ${{\widetilde{H}} }_{\mathcal {C},{\mathcal {D}}}$ in ${\mathfrak H}(\textsf{N}_{\mathcal {C},{\mathcal {D}}}) \oplus {\mathcal {G}}$. Furthermore, it is a direct consequence of Theorem 4.2 that the self-adjoint relation $S_{{\mathcal {A}},{\mathcal {B}}}$ in ${\mathfrak H}(\textsf{N}_{{\mathcal {A}},{\mathcal {B}}}) \oplus {\mathcal {G}}$ and the analogous symmetric relation $S_{\mathcal {C},{\mathcal {D}}}$ in ${\mathfrak H}(\textsf{N}_{\mathcal {C},{\mathcal {D}}}) \oplus {\mathcal {G}}$ are unitarily equivalent under the mapping *U*, i.e.,

$$ S_{\mathcal {C},{\mathcal {D}}}=\big \{ \{Uh, Uh'\}:\, \{h,h'\} \in S_{{\mathcal {A}},{\mathcal {B}}} \big \}, $$

or, equivalently, $$S_{\mathcal {C},{\mathcal {D}}}=U S_{{\mathcal {A}},{\mathcal {B}}}U^*$$.

The results in Theorem 4.1 and Theorem 4.2 would also make sense if the kernel in (4.1) has κ negative squares (instead of being nonnegative), see [2]. In that case one has to deal with reproducing kernel Pontryagin spaces as in [1].

## Štraus Families in Terms of Weyl Families

The notion of a Štraus family in a Hilbert space H has been introduced to describe a self-adjoint extension in a Hilbert space ${{\widetilde{\mathfrak H}} }$ of a symmetric relation *S* in H, when the extension goes beyond the original space H, that is, when ${\mathfrak H}\subset {{\widetilde{\mathfrak H}} }$. Here Štraus families will be interpreted as Weyl families of an appropriately chosen symmetric relation; see [36–39] and [9, 20, 32].

Let ${{\widetilde{H}} }$ be a self-adjoint relation in a Hilbert space ${{\widetilde{\mathfrak H}} }$ and assume that ${{\widetilde{\mathfrak H}} }$ has the orthogonal decomposition

5.1 $$\begin{aligned} {{\widetilde{\mathfrak H}} }={\mathfrak H}_2 \oplus {\mathfrak H}, \end{aligned}$$

with the Hilbert spaces ${\mathfrak H}_2$ and H. Associated with the self-adjoint relation ${{\widetilde{H}} }$ in ${{\widetilde{\mathfrak H}} }$ is the closed symmetric relation $S_2$ in ${\mathfrak H}_2$, given by

5.2 $$\begin{aligned} S_2=\left\{ \{ f_2, f_2'\}:\, \left\{ \begin{pmatrix} f_2 \\ 0 \end{pmatrix}, \begin{pmatrix} f_2' \\ 0\end{pmatrix} \right\} \in {{\widetilde{H}} } \right\} , \end{aligned}$$

and, likewise, the linear relation ${\mathcal {T}}_2$ in ${\mathfrak H}_2$, given by

$$ {\mathcal {T}}_2=\left\{ \{ f_2, f_2'\}:\, \left\{ \begin{pmatrix} f_2 \\ f \end{pmatrix}, \begin{pmatrix} f_2' \\ f' \end{pmatrix} \right\} \in {{\widetilde{H}} } \right\} . $$

Note that one has $$({\mathcal {T}}_2)^{**}=S_2^*$$ or $$S_2=({\mathcal {T}}_2)^{*}$$. Furthermore, define the family of linear relations T(λ), $$\lambda \in {{\mathbb C}\setminus {\mathbb R}}$$, in H by

5.3 $$\begin{aligned} T(\lambda ) :=\left\{ \{f, f'\} :\, \left\{ \begin{pmatrix} f_2 \\ f \end{pmatrix}, \begin{pmatrix} \lambda f_2 \\ f' \end{pmatrix} \right\} \in {{\widetilde{H}} } \right\} , \quad \lambda \in {{\mathbb C}\setminus {\mathbb R}}. \end{aligned}$$

In the next lemma the Hilbert space H serves as the auxiliary space for the boundary relation for $S_2^*$ in ${\mathfrak H}_2$. The orthogonal projection from ${\mathfrak H}_2 \oplus {\mathfrak H}$ onto H is denoted by $P_{\mathfrak H}$. Following [14, 15], the boundary relation will be defined as the inverse main transform of ${{\widetilde{H}} }$; see also [15, Theorem 6.1] for a direct connection to boundary value problems.

### Lemma 5.1

Let ${{\widetilde{H}} }$ be a self-adjoint relation in a Hilbert space ${{\widetilde{\mathfrak H}} }={\mathfrak H}_2 \oplus {\mathfrak H}$. Then the linear relation

5.4 $$\begin{aligned} \Gamma = \left\{ \left\{ \begin{pmatrix} f_2 \\ f_2^\prime \end{pmatrix}, \begin{pmatrix}f \\ -f^\prime \end{pmatrix} \right\} :\, \left\{ \begin{pmatrix} f_2 \\ f \end{pmatrix}, \begin{pmatrix} f_2^\prime \\ f^\prime \end{pmatrix} \right\} \in {{\widetilde{H}} } \right\} \, \end{aligned}$$

is a boundary relation from ${\mathfrak H}_2^2$ to ${\mathfrak H}^2$ for $S_2^*$, where $S_2$ is given by (5.2). Its Weyl family is given by -T(λ), $\lambda \in {{\mathbb C}\setminus {\mathbb R}}$, and the compression of the resolvent $({{\widetilde{H}} } - \lambda )^{-1}$ to H satisfies

5.5 $$\begin{aligned} P_{\mathfrak H}\bigl (\widetilde{H}-\lambda \bigr )^{-1}\upharpoonright {\mathfrak H}=\bigl (T(\lambda )-\lambda \bigr )^{-1},\quad \lambda \in {\mathbb C}\setminus {\mathbb R}, \end{aligned}$$

Furthermore, there is the orthogonal decomposition

5.6 $$\begin{aligned} {\mathfrak H}_2 \oplus {\mathfrak H}=\overline{\textrm{span}} \, \bigl \{{\mathfrak H}, ({{\widetilde{H}} }-\lambda )^{-1} {\mathfrak H}:\, \lambda \in {{\mathbb C}\setminus {\mathbb R}}\bigr \} \oplus {\mathfrak H}((S_2)_{\textrm{sa}}). \end{aligned}$$

Consequently, the boundary relation in (5.4) is minimal if and only if $S_2$ is a simple symmetric operator in ${\mathfrak H}_2$.

### Proof

By Corollary 3.10 the relation in (5.4) is a boundary relation. It follows from (5.4) that it is a boundary relation for $S_2^*$, since its kernel is given by $S_2$, while its domain it given by ${\mathcal {T}}_2$, which is dense in $S_2^*$. It follows from (5.3) and (5.4) that the corresponding Weyl family is given by -T(λ), $\lambda \in {{\mathbb C}\setminus {\mathbb R}}$; cf. Definition 3.3. It is clear from (3.39) and (3.41) that (5.5) and (5.6) hold. □

Observe that the family of linear relations T(λ), $\lambda \in {{\mathbb C}\setminus {\mathbb R}}$, in H defined in (5.3) has the following properties:

1. T(λ) is maximal accumulative (maximal dissipative) for $\lambda \in {\mathbb C}^+$ ($\lambda \in {\mathbb C}^-$);
2. $$T(\lambda )^* =T(\bar{\lambda })$$, $$\lambda \in {{\mathbb C}\setminus {\mathbb R}}$$;
3. the mapping $\lambda \mapsto (T(\lambda )-\lambda )^{-1}$ is holomorphic on ${\mathbb C}\setminus {\mathbb R}$.

This is clear, since -T(λ), $\lambda \in {{\mathbb C}\setminus {\mathbb R}}$, is a Weyl family. Any family of relations T(λ), $\lambda \in {{\mathbb C}\setminus {\mathbb R}}$, in H satisfying (S1), (S2), and (S3) will be called a *Štraus family* in H.

Closely connected with the Štraus family above is the family of *generalized resolvents* consisting of all operators $R(\lambda ) \in {\textbf{B}}({\mathfrak H})$, $\lambda \in {{\mathbb C}\setminus {\mathbb R}}$, which satisfy

1. $$R(\lambda )^* =R(\bar{\lambda })$$, $$\lambda \in {\mathbb C}\setminus {\mathbb R}$$;
2. the kernel
  5.7 $$\begin{aligned} \textsf{R}_R(\lambda , \zeta ) :=\frac{R(\lambda )-R(\zeta )^*}{\lambda -\bar{\zeta }}-R(\lambda )R(\zeta )^*, \quad \lambda , \zeta \in {{\mathbb C}\setminus {\mathbb R}}, \end{aligned}$$
  is nonnegative;
3. the mapping $\lambda \mapsto R(\lambda )$ is holomorphic for $\lambda \in {\mathbb C}\setminus {\mathbb R}$.

Clearly, the compressed resolvent $R(\lambda ) =P_{\mathfrak H}({{\widetilde{H}} }-\lambda )^{-1} \upharpoonright {\mathfrak H}$ of a self-adjoint relation ${{\widetilde{H}} }$ in a Hilbert space ${{\widetilde{\mathfrak H}} }$ with the decomposition (5.1) is a generalized resolvent as follows from the resolvent identity for ${{\widetilde{H}} }$ and its compression to H:

$$\begin{aligned} \textsf{R}_R(\lambda , \zeta ) =P_{\mathfrak H}({{\widetilde{H}} }-\lambda )^{-1} (I-P) ({{\widetilde{H}} } -\zeta )^{-1} \upharpoonright {\mathfrak H}, \quad \lambda , \zeta \in {{\mathbb C}\setminus {\mathbb R}}. \end{aligned}$$

Furthermore, there is a one-to-one correspondence between the Štraus families T(λ), $\lambda \in {{\mathbb C}\setminus {\mathbb R}}$, and the families of generalized resolvents R(λ), $\lambda \in {{\mathbb C}\setminus {\mathbb R}}$, via the identity

5.8 $$\begin{aligned} R(\lambda )=(T(\lambda )-\lambda )^{-1}, \quad \lambda \in {{\mathbb C}\setminus {\mathbb R}}, \end{aligned}$$

or, equivalently,

5.9 $$\begin{aligned} T(\lambda )=\big \{ \{R(\lambda ) h, (I+\lambda R(\lambda ))h \} : h \in {\mathfrak H}\big \}. \end{aligned}$$

The following result is an immediate consequence of Theorem 4.1; cf. [5, Corollary 4.4.7]. The reproducing kernel Hilbert space ${\mathfrak H}(\textsf{R}_{R})$ in the theorem is associated with the generalized resolvent *R* via the reproducing kernel $\textsf{R}_R$ in (5.7). It may be considered as a converse to Lemma 5.1 stated in terms of generalized resolvents.

### Lemma 5.2

Let *R* be a family of generalized resolvents in H which satisfies (R1), (R2), and (R3), and let ${\mathfrak H}(\textsf{R}_{R})$ be the associated reproducing kernel Hilbert space. Then

5.10 $$\begin{aligned} {{\widetilde{H}} }_{R}= \left\{ \left\{ \begin{pmatrix} f \\ h \end{pmatrix}, \begin{pmatrix} f' \\ -h' \end{pmatrix} \right\} :\, \begin{matrix} f,f' \in {\mathfrak H}(\textsf{R}_{R}), \,h, h' \in {\mathfrak H}\\ f'(\xi )-\xi f(\xi ) =(I+\xi R(\xi ))h+R(\xi ) h' \end{matrix} \right\} \end{aligned}$$

is a self-adjoint relation in the Hilbert space ${\mathfrak H}(\textsf{R}_{R}) \oplus {\mathfrak H}$ and the compressed resolvent of ${{\widetilde{H}} }_R$ onto H is given by

$$ P_{\mathfrak H}({{\widetilde{H}} }_R-\lambda )^{-1} \upharpoonright {\mathfrak H}=R(\lambda ), \quad \lambda \in {{\mathbb C}\setminus {\mathbb R}}. $$

Furthermore, the self-adjoint relation ${{\widetilde{H}} }_R$ satisfies the following minimality condition

$$ {\mathfrak H}(\textsf{R}_{R}) \oplus {\mathfrak H}=\overline{\textrm{span}} \, \bigl \{{\mathfrak H}, ({{\widetilde{H}} }_{R}-\lambda )^{-1} {\mathfrak H}:\, \lambda \in {{\mathbb C}\setminus {\mathbb R}}\bigr \}. $$

### Proof

By means of the generalized resolvents R(λ) one introduces the pair $\{{\mathcal {U}}(\lambda ),{\mathcal {V}}(\lambda )\}$ by

$$ {\mathcal {U}}(\lambda )= -R(\lambda ), \,\, {\mathcal {V}}(\lambda )=I+\lambda R(\lambda ), $$

which means that it is a Nevanlinna pair for -T(λ). With this notation the kernel in (5.7) can be written as

5.11 $$\begin{aligned} \textsf{R}_R=\textsf{N}_{{\mathcal {U}},{\mathcal {V}}}, \quad \text{ so } \text{ that } \quad {\mathfrak H}(\textsf{R}_R)={\mathfrak H}( \textsf{N}_{{\mathcal {U}},{\mathcal {V}}}). \end{aligned}$$

Thus the result follows from Theorem 4.1. □

Observe that the corresponding Štraus family T(λ), $$\lambda \in {{\mathbb C}\setminus {\mathbb R}}$$, is visible in Lemma 5.2. Assume that one has $f^{\prime }=\lambda f$ in (5.10). Then it follows by choosing $$\xi =\lambda $$ in the identity $$\lambda f(\xi )-\xi f(\xi ) =(I+\xi R(\xi ))h+R(\xi ) h'$$ that

$$ (I+\lambda R(\lambda ))h+R(\lambda ) h'=0. $$

Hence, it follows from (2.3) that $\{h,h'\} \in T(\lambda )$.

Let *S* be a closed symmetric linear relation in a Hilbert space H. A linear relation *H* in H is said to be an *intermediate extension* of *S* if $S \subset H \subset S^*$, so that also $H^*$ is an intermediate extension of *S*. When considering self-adjoint extensions in ${\mathfrak H_2} \oplus {\mathfrak H}$ of a given symmetric relation *S* in H (identifying $f \in {\mathfrak H}$ with $$\left( {\begin{array}{c}0\\ f\end{array}}\right) $$ in ${\mathfrak H_2} \oplus {\mathfrak H}$), then one meets a Štraus family T(λ), $\lambda \in {{\mathbb C}\setminus {\mathbb R}}$, in H.

### Lemma 5.3

Let *S* be a closed symmetric relation in H and let ${{\widetilde{H}} }$ be a self-adjoint relation in $\widetilde{\mathfrak H}={\mathfrak H_2} \oplus {\mathfrak H}$. Let T(λ), $\lambda \in {{\mathbb C}\setminus {\mathbb R}}$, be the induced Štraus family and let R(λ), $\lambda \in {{\mathbb C}\setminus {\mathbb R}}$, be the induced family of generalized resolvents. Then the following statements are equivalent:

(i) $$S \subset {{\widetilde{H}} }$$;
(ii) $$S\subset T(\lambda ) \subset S^*$$, $$\lambda \in {\mathbb C}\setminus {\mathbb R}$$;
(iii) $$R(\lambda )(S-\lambda ) \subset I$$, $$\lambda \in {\mathbb C}\setminus {\mathbb R}$$.

### Proof

Observe that $T(\lambda )^*=T(\bar{\lambda })$, $\lambda \in {{\mathbb C}\setminus {\mathbb R}}$. Therefore it is clear that $S \subset T(\lambda )$ for all $\lambda \in {{\mathbb C}\setminus {\mathbb R}}$ is equivalent to $T(\lambda ) \subset S^*$ for all $\lambda \in {{\mathbb C}\setminus {\mathbb R}}$.

(i) ⇒ (ii) Assume that $S \subset {{\widetilde{H}} }$. Then it is clear from (5.3) that $S \subset T(\lambda )$, $\lambda \in {{\mathbb C}\setminus {\mathbb R}}$. Hence (ii) follows.

(ii) ⇒ (i) Assume that $S \subset T(\lambda )$, $\lambda \in {{\mathbb C}\setminus {\mathbb R}}$, and let $\{f,f'\} \in S$. Hence, there exists $f_2 \in {\mathfrak H}_2$ such that (5.3) holds for all $\lambda \in {{\mathbb C}\setminus {\mathbb R}}$. Since $\mathrm{Im\,}(f',f)_{\mathfrak H}=0$, it follows that $(\mathrm{Im\,}\lambda ) (f_2, f_2)_{{\mathfrak H}_2}=0$ or $f_2=0$. Thus $\{f,f'\} \in {{\widetilde{H}} }$ and $S \subset {{\widetilde{H}} }$. Hence (i) follows.

(ii) ⇒ (iii) Assume that $S \subset T(\lambda )$, $\lambda \in {{\mathbb C}\setminus {\mathbb R}}$. Since $\lambda \in {{\mathbb C}\setminus {\mathbb R}}$, one now sees that $R(\lambda )(S-\lambda ) \subset R(\lambda ) (T(\lambda )-\lambda ) \subset I$. Hence (iii) follows.

(iii) ⇒ (ii) Assume that $R(\lambda )(S-\lambda ) \subset I$. Let $\{f, f'\} \in S$, so that $\{f, f'-\lambda f \} \in S-\lambda$. This leads to $R(\lambda )(f'-\lambda f)=f$ or $\{f, f'-\lambda f \} \in R(\lambda )^{-1}$, which gives $\{f, f'\} \in R(\lambda )^{-1}+\lambda =T(\lambda )$ and $S \subset T(\lambda )$ follows. □

Self-adjoint extensions of a symmetric relation in a Hilbert space, relative to an exit space which is a Pontryagin space, were considered [21–23].

## Boundary Relations of Bounded Type

The class of boundary relations and corresponding Weyl families has several subclasses which can be characterized in terms of either geometric and functional analytic properties of the boundary relations or via the analytic properties of their Weyl families; see [14–16]. In this section some attention will be paid to the special class of boundary relations whose Weyl family is a bounded operator-valued Nevanlinna function. It will be needed in Sect. 7 and Sect. 8. Such boundary relations have their origin in the notion of generalized boundary triplets that were initially introduced in [18]. In this context one should also mention the constructions in [6–8]; see also [11, 12, 16].

Recall that the boundary relations Γ from ${\mathfrak H}^2$ to ${\mathcal {G}}^2$ are given in Definition 3.1 and their Weyl families in Definition 3.3. The associated relations $\Gamma _0$ and $\Gamma _1$ from ${\mathfrak H}^2$ to ${\mathcal {G}}$ are introduced in (3.18) and (3.19). The next preliminary result is a straightforward observation; cf. [14, Proposition 4.7].

### Lemma 6.1

Let Γ be a boundary relation from ${\mathfrak H}^2$ to ${\mathcal {G}}^2$ with Weyl family Z(λ), $\lambda \in {{\mathbb C}\setminus {\mathbb R}}$. Then the following statements are equivalent:

(i) $$Z(\lambda ) \in {\textbf{B}}({\mathcal {G}})$$, $$\lambda \in {{\mathbb C}\setminus {\mathbb R}}$$;
(ii) $$\Gamma _0( {{\widehat{\mathfrak N}} }_\lambda ({\mathcal {T}}))={\mathcal {G}}$$, $$\lambda \in {{\mathbb C}\setminus {\mathbb R}}$$.

### Proof

(i) ⇒ (ii) Assume that $Z(\lambda ) \in {\textbf{B}}({\mathcal {G}})$, which implies that ${\text {dom }}Z(\lambda )={\mathcal {G}}$. Then (ii) follows from (3.27).

(ii) ⇒ (i) Assume $\Gamma _0( {{\widehat{\mathfrak N}} }_\lambda ({\mathcal {T}}))={\mathcal {G}}$. Now (3.27) implies that ${\text {dom }}Z(\lambda )={\mathcal {G}}$. Then $\mathrm{mul\,}Z(\bar{\lambda })=({\text {dom }}Z(\lambda ))^\perp$, $\lambda \in {{\mathbb C}\setminus {\mathbb R}}$, shows that Z(λ) is an operator (which is closed and everywhere defined). Thus (i) follows by the closed graph theorem. □

Closely related to Lemma 6.1 is the following subclass of boundary relations that will play an important role in the development of the Kreĭn formula in Sect. 7; cf. [14, Section 5.3].

### Definition 6.2

Let H and ${\mathcal {G}}$ be Hilbert spaces and let Γ be a linear relation from ${\mathfrak H}^2$ to ${\mathcal {G}}^2$. Then Γ is said to be of *bounded type* if the following conditions are satisfied:

1. the abstract Green’s identity
  6.1 $$\begin{aligned} (f^\prime ,g)_{\mathfrak H}-(f,g^\prime )_{\mathfrak H}=(\varphi ^\prime , \psi )_{\mathcal {G}}-(\varphi , \psi ^\prime )_{\mathcal {G}}\end{aligned}$$
  holds for all $\{{{\widehat{f}} },{{\widehat{\varphi }} }\}$, $\{{{\widehat{g}} },{{\widehat{\psi }} }\}\in \Gamma$;
2. $${\text {ran }}\Gamma _0={\mathcal {G}}$$;
3. $S_0:=\ker \Gamma _0$ is a self-adjoint relation in H.

The following result and its corollary identify the linear relations of bounded type as a special class of boundary relations; see [14, Proposition 5.9], [15, Theorem 3.16], and [16, Sections 7.4 & 7.5]. For the convenience of the reader a proof is included.

### Proposition 6.3

Let H and ${\mathcal {G}}$ be Hilbert spaces and let Γ be a linear relation from ${\mathfrak H}^2$ to ${\mathcal {G}}^2$ which satisfies $\mathrm {(B1)}$, $\mathrm {(B2)}$, and $\mathrm {(B3)}$. Then Γ is a boundary relation for $S^*=(\ker \Gamma )^*$, such that

6.2 $$\begin{aligned} \Gamma _0({{\widehat{\mathfrak N}} }_\lambda ({\mathcal {T}}))={\mathcal {G}}, \quad \lambda \in {{\mathbb C}\setminus {\mathbb R}}. \end{aligned}$$

Conversely, every boundary relation Γ from ${\mathfrak H}^2$ to ${\mathcal {G}}^2$ which satisfies (6.2) also satisfies $\mathrm {(B1)}$, $\mathrm {(B2)}$, and $\mathrm {(B3)}$. In each of these cases the Weyl family belongs to ${\textbf{B}}({\mathcal {G}})$ and $S_0$ is a smooth extension, i.e., $S_0 \subset {\mathcal {T}}$.

### Proof

First assume that Γ is a linear relation from ${\mathfrak H}^2$ to ${\mathcal {G}}^2$ which satisfies $\mathrm {(B1)}$, $\mathrm {(B2)}$, and $\mathrm {(B3)}$. Then the Green’s identity in $\mathrm {(B1)}$ means that Γ satisfies (D2). Next define the subspace ${\mathfrak G}(\Gamma ) \subset {\mathfrak H}^2 \times {\mathcal {G}}^2$ by

6.3 $$\begin{aligned} {\mathfrak G}(\Gamma )=\big \{ \{{{\widehat{\ell }} }, {{\widehat{\eta }} }\} :\, (f^\prime ,\ell )_{\mathfrak H}-(f,\ell ^\prime )_{\mathfrak H}=(\varphi ^\prime , \eta )_{\mathcal {G}}-(\varphi , \eta ^\prime )_{\mathcal {G}}, \,\, \{{{\widehat{f}} }, {{\widehat{\varphi }} }\} \in \Gamma \big \}, \end{aligned}$$

and observe that (B1) shows that $\Gamma \subset {\mathfrak G}(\Gamma )$. Verifying (D3) is equivalent to verifying ${\mathfrak G}(\Gamma ) \subset \Gamma$. For this purpose let $\{{{\widehat{g}} }, {{\widehat{\psi }} }\} \in {\mathfrak G}(\Gamma )$. Since ${{\widehat{\psi }} }=\{\psi , \psi '\}$ one sees from $\mathrm {(B2)}$ that there exists an element of the form

6.4 $$\begin{aligned} \big \{{{\widehat{h}} }, \{\psi , \tau \}\big \} \in \Gamma \subset {\mathfrak G}(\Gamma ), \end{aligned}$$

where ${{\widehat{h}} }=\{h,h'\}\in {\mathfrak H}^2$ and $\tau \in {\mathcal {G}}$. It follows from $\{{{\widehat{g}} }, {{\widehat{\psi }} }\} \in {\mathfrak G}(\Gamma )$ and (6.4) that

6.5 $$\begin{aligned} \big \{ {{\widehat{g}} } -{{\widehat{h}} }, \{0, \psi '-\tau \} \big \}=\{ {{\widehat{g}} }, {{\widehat{\psi }} }\} -\big \{ {{\widehat{h}} }, \{\psi ,\tau \}\big \} \in {\mathfrak G}(\Gamma ). \end{aligned}$$

Hence, by (6.5) and (6.3), applied with ${{\widehat{f}} } \in S_0$, so that $\big \{{{\widehat{f}} }, \{0,\omega \}\big \} \in \Gamma$ for some $\omega \in {\mathcal {G}}$, one obtains

$$ (f', g-h)_{\mathfrak H}-(f, g'-h')_{\mathfrak H}= (\omega ,0)_{\mathcal {G}}-(0, \psi '-\tau )_{\mathcal {G}}=0, $$

or ${{\widehat{g}} } -{{\widehat{h}} } \in S_0^*=S_0$ by $\mathrm {(B3)}$. Therefore there exists some $\rho \in {\mathcal {G}}$ such that

6.6 $$\begin{aligned} \big \{{{\widehat{g}} } -{{\widehat{h}} }, \{0,\rho \}\big \} \in \Gamma \subset {\mathfrak G}(\Gamma ), \end{aligned}$$

which implies together with (6.5) that

6.7 $$\begin{aligned} \{\{0,0\}, \{0, \psi '-\tau -\rho \} \} \in {\mathfrak G}(\Gamma ). \end{aligned}$$

Thus one sees from (6.7) and (6.3), applied with $\{{{\widehat{f}} }, {{\widehat{\varphi }} }\} \in \Gamma$, together with (B2) that for all $\varphi \in {\mathcal {G}}$

$$ (\varphi , \psi '-\tau -\rho )_{\mathcal {G}}=0, $$

which gives $\psi '-\tau -\rho =0$. Therefore it follows from (6.4) and (6.6) that

$$ \{{{\widehat{g}} }, {{\widehat{\psi }} }\}=\big \{{{\widehat{g}} } -{{\widehat{h}} }, \{0, \rho \}\big \} +\big \{{{\widehat{h}} }, \{\psi , \tau \}\big \} \in \Gamma . $$

Thus ${\mathfrak G}(\Gamma ) \subset \Gamma$ and (D3) holds. Since (D2) and (D3) imply (3.5) it follows that Γ is a boundary relation for $S^*=(\ker \Gamma )^*$. By $\mathrm {(B3)}$ the extension $S_0$ of *S* is self-adjoint, which implies that $S^*=S_0 {{\,\mathrm{\, \widehat{+} \,}\,}}{{\widehat{\mathfrak N}} }_\lambda (S^*)$, $\lambda \in {{\mathbb C}\setminus {\mathbb R}}$. Since $S_0 \subset {\mathcal {T}}$ it follows that

$$ {\mathcal {T}}=S_0 {{\,\mathrm{\, \widehat{+} \,}\,}}{{\widehat{\mathfrak N}} }_\lambda ({\mathcal {T}}), \quad \lambda \in {{\mathbb C}\setminus {\mathbb R}}. $$

Using $\mathrm {(B3)}$ one obtains $\Gamma _0({{\widehat{\mathfrak N}} }_\lambda ({\mathcal {T}}))=\Gamma _0({\mathcal {T}})={\mathcal {G}},$ which confirms (6.2).

For the converse statement, assume that Γ is a boundary relation and assume that (6.2) holds. It is clear that $\mathrm {(B1)}$ is satisfied. Moreover (6.2) implies that

$$ {\mathcal {G}}=\Gamma _0({{\widehat{\mathfrak N}} }_\lambda ({\mathcal {T}})) \subset {\text {ran }}\Gamma _0, \quad \lambda \in {{\mathbb C}\setminus {\mathbb R}}, $$

which implies that $\mathrm {(B2)}$ holds. Next it will be shown that (6.2) implies that

6.8 $$\begin{aligned} {\mathcal {T}}=S_0 {{\,\mathrm{\, \widehat{+} \,}\,}}{{\widehat{\mathfrak N}} }_\lambda ({\mathcal {T}}), \quad \lambda \in {{\mathbb C}\setminus {\mathbb R}}. \end{aligned}$$

It suffices to show that the left-hand side is included in the right-hand side. Let ${{\widehat{f}} } \in {\mathcal {T}}$, so that there exists $\{\varphi , \varphi '\} \in {\mathcal {G}}^2$ such that

$$ \big \{{{\widehat{f}} }, \{\varphi , \varphi '\} \big \} \in \Gamma . $$

By (6.2) there exists $f_\lambda \in {{\widehat{\mathfrak N}} }_\lambda ({\mathcal {T}})$ such that $\{{{\widehat{f}} }_\lambda , \varphi \} \in \Gamma _0$, i.e., $\big \{{{\widehat{f}} }_\lambda , \{\varphi , \zeta \} \big \} \in \Gamma$ for some $\zeta \in {\mathcal {G}}$. This implies that

$$ \big \{{{\widehat{f}} }-{{\widehat{f}} }_\lambda , \{0, \varphi '-\zeta \} \big \} \in \Gamma , $$

so that ${{\widehat{g}} }={{\widehat{f}} } -{{\widehat{f}} }_\lambda \in S_0$. This shows ${{\widehat{f}} }={{\widehat{g}} }+{{\widehat{f}} }_\lambda \in S_0 {{\,\mathrm{\, \widehat{+} \,}\,}}{{\widehat{\mathfrak N}} }_\lambda ({\mathcal {T}})$. Consequently, (6.8) holds.

Observe that (6.8) leads to ${\text {ran }}({\mathcal {T}}-\lambda )={\text {ran }}(S_0-\lambda )$, $\lambda \in {{\mathbb C}\setminus {\mathbb R}}$. Now recall from (3.40) that ${\text {ran }}({\mathcal {T}}-\lambda )={\mathfrak H}$, $\lambda \in {{\mathbb C}\setminus {\mathbb R}}$, and $\mathrm {(B3)}$ follows. □

### Corollary 6.4

Every Nevanlinna function belonging to ${\textbf{B}}({\mathcal {G}})$ is the Weyl family of a boundary relation Γ from ${\mathfrak H}^2$ to ${\mathcal {G}}^2$ with the properties $\mathrm {(B1)}$, $\mathrm {(B2)}$, and $\mathrm {(B3)}$.

### Proof

Let Z(λ), $\lambda \in {{\mathbb C}\setminus {\mathbb R}}$, be a Nevanlinna family for which $Z(\lambda ) \in {\textbf{B}}({\mathcal {G}})$. Then by Theorem 4.2 there exists a minimal boundary relation Γ from ${\mathfrak H}^2$ to ${\mathcal {G}}^2$ such that *Z* is its Weyl family. Due to the condition $Z(\lambda ) \in {\textbf{B}}({\mathcal {G}})$ it now follows from Lemma 6.1 and Proposition 6.3 that $\mathrm {(B1)}$, $\mathrm {(B2)}$, and $\mathrm {(B3)}$ are satisfied. □

Based on Corollary 6.4 the class of boundary relations is now further restricted to the class of ordinary boundary triplets; see Remark 3.7. Their Weyl functions belong to ${\textbf{B}}({\mathcal {G}})$ and are uniformly strict; see Definition 2.4.

### Definition 6.5

Let *S* be a closed symmetric relation in a Hilbert space H with equal defect numbers. Then the triplet $\{{\mathcal {G}}, \Gamma _0, \Gamma _1\}$, where ${\mathcal {G}}$ is a Hilbert space and $\Gamma =\{\Gamma _0, \Gamma _1\}$ is a single-valued linear mapping from $S^*$ to ${\mathcal {G}}^2$, is said to be an *ordinary boundary triplet* for $S^*$ if:

1. $${\text {dom }}\Gamma =S^*$$;
2. the abstract Green’s identity
  $$ (f^\prime ,g)_{\mathfrak H}-(f,g^\prime )_{\mathfrak H}=(\varphi ^\prime , \psi )_{\mathcal {G}}-(\varphi , \psi ^\prime )_{\mathcal {G}}$$
  holds for all $\{{{\widehat{f}} },{{\widehat{\varphi }} }\}$, $\{{{\widehat{g}} },{{\widehat{\psi }} }\}\in \Gamma$;
3. the mapping $\Gamma :\, S^* \rightarrow {\mathcal {G}}^2$ is surjective (and continuous).

In the case of an ordinary boundary triplet $\{{\mathcal {G}}, \Gamma _0, \Gamma _1\}$ there is a bijective correspondence between the set of intermediate extensions $S_\Theta \subset S^*$ of *S* and the set of relations Θ in ${\mathcal {G}}$ via

$$ S_\Theta =\{ {{\widehat{f}} } \in S^*:\, \Gamma {{\widehat{f}} } \in \Theta \}, $$

such that $(S_\Theta )^*=S_{\Theta ^*}$; hence the usual notions like (maximal) symmetry, (maximal) dissipativity, (maximal) accumulativity, and self-adjointness are preserved. In particular, $S_0=\ker \Gamma _0$ and $S_1=\ker \Gamma _1$ are self-adjoint. Thus an ordinary boundary triplet satisfies $\mathrm {(B1)}$, $\mathrm {(B2)}$, and $\mathrm {(B3)}$. Hence, by Proposition 6.3 the Weyl family *Z* has the property that $Z(\lambda ) \in {\textbf{B}}({\mathcal {G}})$, $\lambda \in {{\mathbb C}\setminus {\mathbb R}}$. Furthermore, $\gamma (\lambda ) \in {\textbf{B}}({\mathcal {G}}, {\mathfrak H})$ maps ${\mathcal {G}}$ isomorphically onto ${\mathfrak N}_\lambda (S^*)$. Thus, in fact, *Z* is uniformly strict, due to

6.9 $$\begin{aligned} \frac{Z(\lambda )-Z(\mu )^*}{\lambda -\bar{\mu }}= \gamma (\mu )^*\gamma (\lambda ), \quad \lambda , \mu \in {{\mathbb C}\setminus {\mathbb R}}. \end{aligned}$$

Therefore an ordinary boundary triplet in Definition 6.5 is a boundary relation and its Weyl family is uniformly strict. For this, and the converse statement, see [14]. Furthermore, the self-adjoint extension $S_0$ of *S* satisfies

6.10 $$\begin{aligned} (S_0-\lambda )^{-1} \gamma (\bar{\mu }) =\frac{\gamma (\lambda )-\gamma (\bar{\mu })}{\lambda -\bar{\mu }}, \quad \lambda , \mu \in {{\mathbb C}\setminus {\mathbb R}}. \end{aligned}$$

For more information see, for instance, [5, Proposition 2.3.6].

## Krein’s Formula for Boundary Relations

The present resolvent formula of Kreĭn parametrizes extensions of a closed symmetric relation *S* by means of a boundary relation Γ for $S^*$ and a corresponding symmetric extension $S_0$ of *S*, given by $S_0=\ker \Gamma _0$. Observe that the identity (7.1) in the theorem below is meant in the sense of relations. In the special case of boundary triplets an analogous result was established in [11, Theorem 5.8] (where the terminology of unitary boundary triplets was used).

Recall from Sect. 3 that there is a one-to-one correspondence between the linear subspaces *H* in ${\mathfrak H}^2$ with $S=\ker \Gamma \subset H \subset {\mathcal {T}}={\text {dom }}\Gamma$ and the linear subspaces Θ in ${\mathcal {G}}^2$ with $\mathrm{mul\,}\Gamma \subset \Theta \subset {\text {ran }}\Gamma$ via $H=\Gamma ^{-1}(\Theta )$. This correspondence will be denoted by $H=S_\Theta$. Observe that the extension $S_\Theta$ described in the following theorem is automatically smooth. Moreover, recall that $\mathrm{mul\,}Z(\lambda )$ does not depend on $\lambda \in {{\mathbb C}\setminus {\mathbb R}}$, (see item (c) in Lemma 3.4) so that $P_\textrm{op}$ does not depend on $\lambda \in {{\mathbb C}\setminus {\mathbb R}}$, as $(\mathrm{mul\,}\gamma (\bar{\lambda })^*)^\perp =(\mathrm{mul\,}Z(\lambda ))^\perp$; see (3.29).

### Theorem 7.1

Let *S* be a closed symmetric linear relation in H and let Γ from ${\mathfrak H}^2$ to ${\mathcal {G}}^2$ be a boundary relation for $S^*$. Let Z(λ) and $\gamma (\lambda )$, $\lambda \in {{\mathbb C}\setminus {\mathbb R}}$, be the corresponding Weyl family and γ-field, respectively. Then for any linear relation Θ with $\mathrm{mul\,}\Gamma \subset \Theta \subset {\text {ran }}\Gamma$ in ${\mathcal {G}}$ and the corresponding extension $S_\Theta =\Gamma ^{-1}(\Theta )$ of *S* the following equality holds

7.1 $$\begin{aligned} (S_\Theta -\lambda )^{-1}-(S_0-\lambda )^{-1} = \gamma (\lambda )\bigl (\Theta -Z(\lambda )\bigr )^{-1}\gamma (\bar{\lambda })^*, \quad \lambda \in {{\mathbb C}\setminus {\mathbb R}}, \end{aligned}$$

or, equivalently,

7.2 $$\begin{aligned} (S_\Theta -\lambda )^{-1}-(S_0-\lambda )^{-1} = \gamma (\lambda )\bigl (\Theta -Z(\lambda )\bigr )^{-1}P_\textrm{op}\gamma (\bar{\lambda })^*, \quad \lambda \in {{\mathbb C}\setminus {\mathbb R}}, \end{aligned}$$

where $P_\textrm{op}\gamma (\bar{\lambda })^*$ stands for the operator part of $\gamma (\bar{\lambda })^*$, determined by the orthogonal projection $P_\textrm{op}$ onto $(\mathrm{mul\,}\gamma (\bar{\lambda })^*)^\perp$.

### Proof

First the inclusion “⊂” in (7.1) will be proved. Since the extension $S_0$ is symmetric, the inverse relation $(S_0-\lambda )^{-1}$ is a bounded, in general, nondensely defined operator for every fixed $\lambda \in {{\mathbb C}\setminus {\mathbb R}}$.

Let $\{h,h''\}\in (S_\Theta -\lambda )^{-1}-(S_0-\lambda )^{-1}$. Then by definition of the sum it follows that $h\in {\text {dom }}(S_\Theta -\lambda )^{-1} \cap {\text {dom }}(S_0-\lambda )^{-1}$ and there exists some $h' \in {\mathfrak H}$ such that $\{h,h'\} \in (S_\Theta -\lambda )^{-1}$ and one has $h''=h'-(S_0-\lambda )^{-1}h$. Next define the elements

7.3 $$\begin{aligned} {{\widehat{h}} }_\Theta :=\{h',h+\lambda h'\}\in S_\Theta \subset {\text {dom }}\Gamma , \end{aligned}$$

and, since $$h \in {\text {ran }}(S_0-\lambda )$$,

7.4 $$\begin{aligned} {{\widehat{h}} }_0:= \{(S_0-\lambda )^{-1}h,(I+\lambda (S_0-\lambda )^{-1})h\} \in S_{0} \subset {\text {dom }}\Gamma . \end{aligned}$$

Therefore, one sees from (7.3) and (7.4) that

7.5 $$\begin{aligned} {{\widehat{h}} }_\Theta -{{\widehat{h}} }_0 =\{h'-(S_0-\lambda )^{-1}h,\lambda (h'-(S_0-\lambda )^{-1}h)\}=\{h'',\lambda h''\}, \end{aligned}$$

so that ${{\widehat{h}} }_\Theta -{{\widehat{h}} }_0\in {{\widehat{\mathfrak N}} }_{\lambda }({\mathcal {T}})$. Recall that ${{\widehat{\gamma }} }(\lambda )$ maps ${\text {dom }}{{\widehat{\gamma }} }(\lambda )$ onto ${{\widehat{\mathfrak N}} }_{\lambda }({\mathcal {T}})\subset {\text {dom }}\Gamma$ and hence there exists

$$\varphi \in {\text {dom }}{{\widehat{\gamma }} }(\lambda )={\text {dom }}Z(\lambda )$$

such that

7.6 $$\begin{aligned} {{\widehat{h}} }_\Theta - {{\widehat{h}} }_0 = {{\widehat{\gamma }} }(\lambda ) \varphi . \end{aligned}$$

Thus (7.3) and (7.6) lead to

7.7 $$\begin{aligned} {{\widehat{h}} }_0 + {{\widehat{\gamma }} }(\lambda ) \varphi = {{\widehat{h}} }_\Theta \in S_\Theta =\Gamma ^{-1}(\Theta ), \end{aligned}$$

so that by definition there exists ${{\widehat{\omega }} }\in \Theta$ such that

7.8 $$\begin{aligned} \big \{ {{\widehat{h}} }_0+{{\widehat{\gamma }} }(\lambda ) \varphi , {{\widehat{\omega }} }\big \} \in \Gamma . \end{aligned}$$

Due to (7.4) with $h \in {\text {ran }}(S_0-\lambda )$, by Proposition 3.12 there exists $\eta \in {\mathcal {G}}$ with $\{h, \eta \} \in \gamma (\bar{\lambda })^*$, such that

$$ \big \{ {{\widehat{h}} }_0, \{0, \eta \} \big \} \in \Gamma , $$

while by (3.26) one has that

$$ \big \{ {{\widehat{\gamma }} }(\lambda ) \varphi , {{\widehat{\varphi }} }\big \} \in \Gamma , \quad \text{ where } \quad {{\widehat{\varphi }} }=\{\varphi , \varphi '\} \in Z(\lambda ), $$

which leads to

7.9 $$\begin{aligned} \big \{ {{\widehat{h}} }_0+{{\widehat{\gamma }} }(\lambda ) \varphi , \{0, \eta \} + {{\widehat{\varphi }} }\big \} \in \Gamma . \end{aligned}$$

By assumption $\mathrm{mul\,}\Gamma \subset \Theta$, so that it follows from (7.8) and (7.9) that

$$ \{0, \eta \}+\{\varphi , \varphi '\} \in \Theta , $$

and, since $\{\varphi , \varphi '\} \in Z(\lambda )$, this gives

$$ \{\varphi , \eta \}= \{\varphi , \eta + \varphi ' -\varphi '\} \in \Theta -Z(\lambda ). $$

Thus, $\{\eta , \varphi \}\in (\Theta - Z(\lambda ))^{-1}$ and, combined with $\{h, \eta \} \in \gamma (\bar{\lambda })^*$, it follows that $\{h,\varphi \}\in (\Theta - Z(\lambda ))^{-1}\gamma (\bar{\lambda })^*$. Finally, from (7.5) and (7.6) one sees that $h''=\gamma (\lambda ) \varphi$. Consequently, it follows that

$$ \{h,h''\} \in \gamma (\lambda )(\Theta -Z(\lambda ))^{-1}\gamma (\bar{\lambda })^*, $$

which proves the inclusion “⊂” in (7.1).

Next the reverse inclusion “⊃” in (7.1) will be shown. For this purpose let $\{h,h''\}\in \gamma (\lambda )(\Theta -Z(\lambda ))^{-1}\gamma (\bar{\lambda })^*$ with $\lambda \in {{\mathbb C}\setminus {\mathbb R}}$. By definition this implies that

$$ \{h, \eta \} \in \gamma (\bar{\lambda })^*, \quad \{\eta , \varphi \} \in (\Theta -Z(\lambda ))^{-1}, \quad \text{ and } \quad h''=\gamma (\lambda ) \varphi , $$

for elements $\eta \in {\mathcal {G}}$ and $\varphi \in {\mathcal {G}}$. As $\{\varphi ,\eta \} \in \Theta -Z(\lambda )$, one sees that $\{\varphi , \varphi ^\prime \} \in Z(\lambda )$ for some $\varphi ^\prime \in {\mathcal {G}}$ and then $\{\varphi ,\eta +\varphi '\}\in \Theta$. By assumption $\Theta \subset {\text {ran }}\Gamma$ and $S_\Theta =\Gamma ^{-1}(\Theta )$, and hence there exists ${{\widehat{h}} }_{\Theta }\in S_\Theta$ such that

$$\big \{ {{\widehat{h}} }_\Theta , \{\varphi ,\eta +\varphi '\} \big \}\in \Gamma .$$

Now recall that

$$ \bigl \{ \{\widehat{\gamma }(\lambda )\varphi ,\{\varphi , \varphi '\}\}: \{\varphi ,\varphi '\}\in Z(\lambda ) \bigr \} \in \Gamma , $$

and thus in the present situation it follows that

$$ \big \{ {{\widehat{h}} }_\Theta -{{\widehat{\gamma }} }(\lambda ) \varphi , \{0,\eta \} \big \} \in \Gamma . $$

On the other hand, $\{h, \eta \} \in \gamma (\bar{\lambda })^*$ and Proposition 3.12 shows that $h\in {\text {ran }}(S_0-\lambda )$ and

$$ \big \{ \{(S_0-\lambda )^{-1}h,(I+\lambda (S_0-\lambda )^{-1})h\}, \{0,\eta \} \big \} \in \Gamma . $$

Now using $S=\ker \Gamma$, one obtains

$$ {{\widehat{h}} }_\Theta -{{\widehat{\gamma }} }(\lambda ) \varphi -\{(S_0-\lambda )^{-1}h,(I+\lambda (S_0-\lambda )^{-1})h\} \in S \subset S_\Theta , $$

and, since ${{\widehat{h}} }_\Theta \in S_\Theta$, one concludes that

$$ {{\widehat{\gamma }} }(\lambda ) \varphi +\{(S_0-\lambda )^{-1}h,(I+\lambda (S_0-\lambda )^{-1})h\} \in S_\Theta . $$

Writing this inclusion in an alternative way, with $\lambda \in {{\mathbb C}\setminus {\mathbb R}}$, one sees that

$$ \{h, (S_0-\lambda )^{-1}h + \gamma (\lambda ) \varphi \} \in (S_\Theta -\lambda )^{-1}. $$

Therefore, one concludes that $h \in {\text {dom }}(S_\Theta -\lambda )^{-1} \cap {\text {dom }}(S_0-\lambda )^{-1}$ and

$$ \{h,h''\}= \{h,\gamma (\lambda )\varphi \} \in (S_\Theta -\lambda )^{-1}-(S_0-\lambda )^{-1}, $$

which proves the inclusion “⊃” in (7.1). This completes the proof of the formula  (7.1).

Finally, to prove (7.2) observe that

$$ \mathrm{mul\,}Z(\lambda )\subset \mathrm{mul\,}(\Theta -Z(\lambda ))=\ker (\Theta -Z(\lambda ))^{-1}, \quad \lambda \in {{\mathbb C}\setminus {\mathbb R}}. $$

Therefore, with the orthogonal projection $P_\textrm{op}$ onto the subspace

$$\begin{aligned} (\mathrm{mul\,}\gamma (\bar{\lambda })^*)^\perp =(\mathrm{mul\,}Z(\lambda ))^\perp , \end{aligned}$$

one can rewrite the formula (7.1) in the form (7.2). □

It is emphasized that under the present circumstances the relation $S_0$ may even coincide with *S*; see, e.g. [14, Example 6.6]. There are a couple of useful consequences of Theorem 7.1. Recall first from Proposition 3.12, cf. (3.52), that the following equalities hold for all $\lambda \in {{\mathbb C}\setminus {\mathbb R}}$:

7.10 $$\begin{aligned} {\text {dom }}\gamma (\bar{\lambda })^*={\text {dom }}(S_0-\lambda )^{-1} \; \text{ and } \; \ker \gamma (\bar{\lambda })^*={\text {dom }}(S-\lambda )^{-1}. \end{aligned}$$

Furthermore, notice that

7.11 $$\begin{aligned} {\text {ran }}(\Theta -Z(\lambda ))^{-1}\subset {\text {dom }}Z(\lambda )={\text {dom }}\gamma (\lambda ), \end{aligned}$$

so that it follows from the formula (7.1) that for all $\lambda \in {{\mathbb C}\setminus {\mathbb R}}$,

$$ {\text {dom }}(S_\Theta -\lambda )^{-1}\cap {\text {dom }}(S_0-\lambda )^{-1} ={\text {dom }}(\Theta -Z(\lambda ))^{-1}\gamma (\bar{\lambda })^*. $$

The following lemma will be helpful to specialize the formulas (7.1), (7.2) in Theorem 7.1 in the particular case where $S_0=\ker \Gamma _0$ is self-adjoint.

### Lemma 7.2

Let the assumptions be as in Theorem 7.1. Then:

- $S_0$ is self-adjoint if and only if for some $\lambda \in {\mathbb C}^+$ and some $\lambda \in {\mathbb C}^-$ the operator $\gamma (\lambda )$ is bounded (not necessarily densely defined).
- if (a) holds, then $\gamma (\lambda )$ is a bounded, not necessarily densely defined, operator and the operator part $P_\textrm{op}\gamma (\bar{\lambda })^*$ of $\gamma (\bar{\lambda })^*$ belongs to ${\textbf{B}}({\mathfrak H},{\mathcal {G}})$ for all $\lambda \in {{\mathbb C}\setminus {\mathbb R}}$.

### Proof

Assume that $S_0$ is self-adjoint. Then ${\text {dom }}(S_0-\lambda )^{-1}={\mathfrak H}$ for all $\lambda \in {{\mathbb C}\setminus {\mathbb R}}$. By the first identity in (7.10) one has ${\text {dom }}\gamma (\bar{\lambda })^*={\mathfrak H}$. Hence $\gamma (\bar{\lambda })$ is closable and its closure also has a closed domain; see, e.g. [5, Theorem 1.3.5]. By the closed graph theorem the closure of $\gamma (\bar{\lambda })$ is a bounded operator, but not necessarily densely defined, since the Weyl family Z(λ) can be multivalued; see [11, Theorem 5.17, eq. (5.31)]. In this case $\gamma (\bar{\lambda })^*$ is multivalued. In any case, the operator part $P_\textrm{op}\gamma (\bar{\lambda })^*$ of $\gamma (\bar{\lambda })^*$ is bounded and everywhere defined on H. This proves the implication “⇒” in (a) and the statement in (b).

To prove the implication “⇐” in statement (a), assume that $\gamma (\lambda )$ is a bounded, not necessarily densely defined, operator for some $\lambda \in {\mathbb C}^+$ and some $\lambda \in {\mathbb C}^-$. Then ${\text {dom }}\gamma (\lambda )^*={\mathfrak H}$ for some $\lambda \in {\mathbb C}^+$ and some $\lambda \in {\mathbb C}^-$. Now it follows from the first identity in (7.10) that $S_0$ is self-adjoint; see (3.52). This completes the proof. □

The next result shows that the general resolvent type formulas in Theorem 7.1 can be used to derive information from the spectral properties of the extensions $S_\Theta$ of *S*.

### Proposition 7.3

Let the assumptions be as in Theorem 7.1. Then the following statements hold for all $\lambda \in {{\mathbb C}\setminus {\mathbb R}}$:

- $\lambda \in \sigma _p(S_\Theta )$ if and only if
  $$ 0\in \sigma _p(\Theta -Z(\lambda )) \quad \text{ and } \quad \gamma (\lambda )\ker (\Theta -Z(\lambda ))\ne \{0\}, \quad \lambda \in {{\mathbb C}\setminus {\mathbb R}}, $$
  and in this case one has
  $$ \ker (S_\Theta -\lambda )=\gamma (\lambda )\ker (\Theta -Z(\lambda )), \quad \lambda \in {{\mathbb C}\setminus {\mathbb R}}. $$
- Assume that $S_0$ is self-adjoint. If $(\Theta -Z(\lambda ))^{-1}$ is a bounded, not necessarily densely defined operator, then $(S_\Theta -\lambda )^{-1}$ is also a bounded, not necessarily densely defined, operator. Moreover,
  $$ (\Theta -Z(\lambda ))^{-1}\in {\textbf{B}}({\mathcal {G}}) \quad \Rightarrow \quad \lambda \in \rho (S_\Theta ), \quad \lambda \in {{\mathbb C}\setminus {\mathbb R}}, $$
  and, in particular, the extension $S_\Theta$ of *S* is closed.

### Proof

(a) First observe that, since $(S_0-\lambda )^{-1}$ is a bounded operator and $\gamma (\lambda )$ is an operator, (7.2) shows that $(S_\Theta -\lambda )^{-1}$ is multivalued precisely when $\gamma (\lambda )(\Theta -Z(\lambda ))^{-1}$ is multivalued.

Now assume that $\lambda \in \sigma _p(S_\Theta )$. Then $\mathrm{mul\,}(S_\Theta -\lambda )^{-1}\ne \{0\}$ and thus also $\mathrm{mul\,}\gamma (\lambda )(\Theta -Z(\lambda ))^{-1}\ne \{0\}$ which implies that $\mathrm{mul\,}(\Theta -Z(\lambda ))^{-1}\ne \{0\}$, since $\gamma (\lambda )$ is an operator. Hence, $\ker (\Theta -Z(\lambda ))\ne \{0\}$ and $0\in \sigma _p(\Theta -Z(\lambda ))$. Moreover, it is clear from (7.2) that $\gamma (\lambda )$ maps $\mathrm{mul\,}(\Theta -Z(\lambda ))^{-1}$ onto $\mathrm{mul\,}(S_\Theta -\lambda )^{-1}$. This proves the second statement in (a).

Next assume that $0\in \sigma _p(\Theta -Z(\lambda ))$ and $\gamma (\lambda )\ker (\Theta -Z(\lambda ))\ne \{0\}$. Then $\mathrm{mul\,}(\Theta -Z(\lambda ))^{-1}\ne \{0\}$ and thus also $\mathrm{mul\,}(S_\Theta -\lambda )^{-1}\ne \{0\}$, i.e., $\lambda \in \sigma _p(S_\Theta )$.

(b) Assume that $S_0$ is self-adjoint. Then by Lemma 7.2 (b) $\gamma (\lambda )$ and $P_\textrm{op}\gamma (\bar{\lambda })^*$ are bounded operators for all $\lambda \in {{\mathbb C}\setminus {\mathbb R}}$. Hence, if $(\Theta -Z(\lambda ))^{-1}$ is a bounded, not necessarily densely defined, operator, then it is clear from (7.2) that also $(S_\Theta -\lambda )^{-1}$ is a bounded, not necessarily densely defined, operator for all $\lambda \in {{\mathbb C}\setminus {\mathbb R}}$. Similarly, if $(\Theta -Z(\lambda ))^{-1}\in {\textbf{B}}({\mathcal {G}})$ then Lemma 7.2 combined with the inclusion in (7.11) and the formula (7.2) shows that $(S_\Theta -\lambda )^{-1}\in {\textbf{B}}({\mathfrak H})$ for all $\lambda \in {{\mathbb C}\setminus {\mathbb R}}$. This completes the proof. □

As a further application of Theorem 7.1 Kreĭn’s resolvent formula for self-adjoint exit space extensions of a closed symmetric relation with equal defect numbers will be derived in Sect. 8. In that case $S_\Theta \,(={{\widetilde{H}} }={{\widehat{\Gamma }} }^{-1}({{\widetilde{\Theta }} }))$ will be self-adjoint, so that the first term corresponding to the one in (7.1) is a standard resolvent operator, and the term corresponding to $(\Theta -Z(\lambda )\bigr )^{-1}$ belongs to ${\textbf{B}}({\mathcal {G}}^2)$; for details, see the Kreĭn type formula in (8.14), which is used in the proof of Theorem 8.2.

## Coupling of Boundary Relations and Boundary Triplets

In this section a general version of the orthogonal coupling technique is presented which uses an orthogonal sum of an ordinary boundary triplet $\{{\mathcal {G}}, \Gamma _0^\circ , \Gamma _1^\circ \}$ with the Weyl function *M* and a general boundary relation Γ whose Weyl family is allowed to be an arbitrary Nevanlinna family *Z*.

### Orthogonal Coupling and Consequences

The situation is centered on a closed symmetric relation *A* in a Hilbert space H with equal defect numbers and a closed symmetric relation *S* in a Hilbert space ${\mathfrak H}'$. Let $\{{\mathcal {G}}, \Gamma _0^{\circ },\Gamma _1^{\circ }\}$ be an ordinary boundary triplet for $A^*$ whose γ-field is $\gamma ^{\circ }(\lambda )$ and whose Weyl function is M(λ), $\lambda \in {{\mathbb C}\setminus {\mathbb R}}$. Let Γ from $({\mathfrak H}')^2$ to ${\mathcal {G}}^2$ be a boundary relation for $S^*$ with ${\mathcal {T}}={\text {dom }}\Gamma$ whose γ-field is $\gamma (\lambda )$ and whose Weyl family is Z(λ), $\lambda \in {{\mathbb C}\setminus {\mathbb R}}$. Recall that the transposed boundary relation $\Gamma ^\top$, defined by the composition

$$ \Gamma ^\top = \begin{pmatrix} 0 &  I_{\mathcal {G}}\\ -I_{\mathcal {G}}&  0 \end{pmatrix} \circ \Gamma , $$

has the same domain ${\mathcal {T}}={\text {dom }}\Gamma ={\text {dom }}\Gamma ^\top$; the corresponding γ-field is denoted by $\gamma ^\top (\lambda )$ and the corresponding Weyl family is given by $Z^\top (\lambda )=-Z(\lambda )^{-1}$, $\lambda \in {{\mathbb C}\setminus {\mathbb R}}$; see (3.30), (3.31), and (3.32).

It is easy to see that the orthogonal sum ${{\widehat{S}} }:=A {{\,\mathrm{\, \widehat{\oplus }\,}\,}}S$ is a closed symmetric relation in ${\mathfrak H}\oplus {\mathfrak H}'$ and that $\Gamma ^{\circ }{{\,\mathrm{\, \widehat{\oplus }\,}\,}}\Gamma ^\top$ is a boundary relation for ${{\widehat{S}} }^*=A^*{{\,\mathrm{\, \widehat{\oplus }\,}\,}}S^*$ given by

8.1 $$\begin{aligned} {{\widehat{\Gamma }} }:=\Gamma ^{\circ }{{\,\mathrm{\, \widehat{\oplus }\,}\,}}\Gamma ^\top =\left\{ \left\{ \, {{\widehat{f}} } \oplus {{\widehat{g}} }, \left\{ \left( {\begin{array}{c}\Gamma _0^{\circ }{{\widehat{f}} }\;\\ \psi '\end{array}}\right) ,\left( {\begin{array}{c}\Gamma _1^{\circ }{{\widehat{f}} }\;\\ -\psi \end{array}}\right) \right\} \right\} :\, {{\widehat{f}} } \in A^*, \; \{{{\widehat{g}} },{{\widehat{\psi }} }\} \in \Gamma \right\} , \end{aligned}$$

with domain ${\text {dom }}{{\widehat{\Gamma }} }=A^* {{\,\mathrm{\, \widehat{\oplus }\,}\,}}{\mathcal {T}}$ and ${\text {ran }}{{\widehat{\Gamma }} }$ determined by

8.2 $$\begin{aligned} {\text {ran }}{{\widehat{\Gamma }} }= \left\{ \, \left\{ \left( {\begin{array}{c}\varphi \\ \psi '\end{array}}\right) , \left( {\begin{array}{c}\varphi '\\ -\psi \end{array}}\right) \right\} :\, \varphi , \varphi ' \in {\mathcal {G}}, \,\, {{\widehat{\psi }} }\in {\text {ran }}\Gamma \right\} , \end{aligned}$$

since $\{{\mathcal {G}}, \Gamma _0^\circ , \Gamma _1^\circ \}$ is an ordinary boundary triplet. Note, in particular, that the multivalued part $\mathrm{mul\,}{{\widehat{\Gamma }} }$ is determined by

8.3 $$\begin{aligned} \mathrm{mul\,}{{\widehat{\Gamma }} }= \left\{ \, \left\{ \left( {\begin{array}{c}0\\ \eta '\end{array}}\right) , \left( {\begin{array}{c}0\\ -\eta \end{array}}\right) \right\} :\, {{\widehat{\eta }} }\in \mathrm{mul\,}\Gamma \right\} . \end{aligned}$$

The corresponding γ-field and Weyl family are given by

8.4 $$\begin{aligned} {{\widehat{\gamma }} }(\lambda )= \begin{pmatrix} \gamma ^{\circ }(\lambda ) &  0 \\ 0 &  \gamma ^\top (\lambda ) \end{pmatrix} \quad \text {and}\quad {{\widehat{M}} }(\lambda )= \begin{pmatrix} M(\lambda ) &  0 \\ 0 &  -Z(\lambda )^{-1} \end{pmatrix}, \quad \lambda \in {{\mathbb C}\setminus {\mathbb R}}. \end{aligned}$$

Recall that $A_0=\ker \Gamma _0^{\circ }$ is a self-adjoint extension of *A* in H and $S_1=\ker \Gamma _1$ is a symmetric extension of *S* in ${\mathfrak H}'$, which is not necessarily self-adjoint; cf. (3.20) and (3.21). Moreover, it follows from (8.1) that

8.5 $$\begin{aligned} {{\widehat{S}} }_0:=\ker {{\widehat{\Gamma }} }_0=\ker \Gamma _0^{\circ } {{\,\mathrm{\, \widehat{\oplus }\,}\,}}\ker \Gamma _1=A_0 {{\,\mathrm{\, \widehat{\oplus }\,}\,}}S_1 \end{aligned}$$

is a symmetric extension of ${{\widehat{S}} }$.

Next the boundary relation ${{\widehat{\Gamma }} }=\Gamma ^{\circ }{{\,\mathrm{\, \widehat{\oplus }\,}\,}}\Gamma ^\top$ in (8.1) for ${{\widehat{S}} }^*$ will be transformed in a suitable way by the following operator $W\in {\textbf{B}}({\mathcal {G}}^2\oplus {\mathcal {G}}^2)$:

8.6 $$\begin{aligned} W=\begin{pmatrix} W_{00} &  -I_{{\mathcal {G}}^2} \\ I_{{\mathcal {G}}^2} &  0 \end{pmatrix}, \text { where} \quad W_{00}=\begin{pmatrix} 0 &  I_{\mathcal {G}}\\ I_{\mathcal {G}}&  0 \end{pmatrix}. \end{aligned}$$

In the terminology of Remark 3.7 the operator *W* in (8.6) is a standard unitary operator in the Kreĭn space $({\mathcal {G}}^2\oplus {\mathcal {G}}^2,J_{{\mathcal {G}}^2})$. Hence ${{\widetilde{\Gamma }} }=W \circ {{\widehat{\Gamma }} }$ is a new boundary relation for ${{\widehat{S}} }^*$ which has a remarkable property; see [13, Theorem 5.6] and [15, Theorem  5.12].

#### Lemma 8.1

Let $W\in {\textbf{B}}({\mathcal {G}}^2\oplus {\mathcal {G}}^2)$ be the operator in (8.6) and define ${{\widetilde{\Gamma }} }=W \circ {{\widehat{\Gamma }} }$. Then ${{\widetilde{\Gamma }} }$ is a boundary relation for ${{\widehat{S}} }^*=A^*{{\,\mathrm{\, \widehat{\oplus }\,}\,}}S^*$ of the form

8.7 $$\begin{aligned} {{\widetilde{\Gamma }} }=\left\{ \left\{ {{\widehat{f}} }\oplus {{\widehat{g}} }, \left\{ \left( {\begin{array}{c}\psi '-\Gamma _1^{\circ }{{\widehat{f}} }\;\\ \psi + \Gamma _0^{\circ }{{\widehat{f}} }\;\end{array}}\right) ,\left( {\begin{array}{c}\Gamma _0^{\circ }{{\widehat{f}} }\;\\ \psi '\end{array}}\right) \right\} \right\} :\, {{\widehat{f}} } \oplus {{\widehat{g}} } \in A^*{{\,\mathrm{\, \widehat{\oplus }\,}\,}}S^*, \,\, \{{{\widehat{g}} },{{\widehat{\psi }} }\} {\in } \Gamma \right\} .\nonumber \\ \end{aligned}$$

Moreover, the corresponding Weyl function ${{\widetilde{M}} }(\lambda )\in {\textbf{B}}({\mathcal {G}}^2)$ is given by

8.8 $$\begin{aligned} {{\widetilde{M}} }(\lambda )= \small { \begin{pmatrix} -(Z(\lambda )+M(\lambda ))^{-1} &  I-(Z(\lambda )+M(\lambda ))^{-1}M(\lambda ) \\ I- M(\lambda )(Z(\lambda )+M(\lambda ))^{-1} &  M(\lambda )- M(\lambda )(Z(\lambda )+M(\lambda ))^{-1}M(\lambda ) \end{pmatrix}. } \end{aligned}$$

Consequently, ${{\widetilde{H}} }:=\ker {{\widetilde{\Gamma }} }_0$ is a self-adjoint extension of $A {{\,\mathrm{\, \widehat{\oplus }\,}\,}}S$.

#### Proof

First of all it is easily verified that *W* in (8.6) is a standard unitary operator. The rest of the proof is based on the transformation properties mentioned in Remark 3.7. A straightforward calculation using the elements from the range of ${{\widehat{\Gamma }} }$ in (8.2) shows that

$$ W\left\{ \left( {\begin{array}{c}\Gamma _0^{\circ }{{\widehat{f}} }\;\\ \psi '\end{array}}\right) ,\left( {\begin{array}{c}\Gamma _1^{\circ }{{\widehat{f}} }\;\\ -\psi \end{array}}\right) \right\} =\left\{ \left( {\begin{array}{c}\psi '-\Gamma _1^{\circ }{{\widehat{f}} }\;\\ \Gamma _0^{\circ }{{\widehat{f}} }+\psi \end{array}}\right) ,\left( {\begin{array}{c}\Gamma _0^{\circ }{{\widehat{f}} }\;\\ \psi '\end{array}}\right) \right\} , $$

which confirms (8.7). The Weyl family in (8.4) is transformed by the corresponding formula $W[M(\lambda ){{\,\mathrm{\, \widehat{\oplus }\,}\,}}-Z(\lambda )^{-1}]$, see Remark 3.7:

8.9 $$\begin{aligned} W\left\{ \left( {\begin{array}{c}\varphi \\ \psi '\end{array}}\right) ,\left( {\begin{array}{c}M(\lambda )\varphi \\ -\psi \end{array}}\right) \right\} =\left\{ \left( {\begin{array}{c}\psi ' - M(\lambda )\varphi \\ \varphi +\psi \end{array}}\right) ,\left( {\begin{array}{c}\varphi \\ \psi '\end{array}}\right) \right\} , \end{aligned}$$

for all $\varphi \in {\mathcal {G}}$ and $\{\psi ,\psi '\}\in Z(\lambda )$. Comparing this with the formula in (2.9) one concludes from Lemma 2.5 that the Weyl function ${{\widetilde{M}} }(\lambda )$ of the transformed boundary relation ${{\widetilde{\Gamma }} }$ coincides with the operator-valued Nevanlinna function $-\Omega (\lambda )^{-1}$ in (2.8) which belongs to ${\textbf{B}}({\mathcal {G}}^2)$. According to Proposition 6.3 this means that ${{\widetilde{\Gamma }} }$ is a boundary relation which satisfies the properties (B1)–(B3). In particular, by the property (B3) the extension ${{\widetilde{H}} }=\ker {{\widetilde{\Gamma }} }_0$ of $A{{\,\mathrm{\, \widehat{\oplus }\,}\,}}S$ is self-adjoint. □

### The Kreĭn Formula

The self-adjoint relation ${{\widetilde{H}} }$ in Lemma 8.1 is a self-adjoint extension of $A {{\,\mathrm{\, \widehat{\oplus }\,}\,}}S$. It will be helpful to describe ${{\widetilde{H}} }$ in terms of the original boundary relation ${{\widehat{\Gamma }} }$ in (8.1).

#### Theorem 8.2

Let *A* be a closed symmetric relation in H with equal defect numbers, and let *S* be a closed symmetric relation in ${\mathfrak H}^\prime$, let $\{{\mathcal {G}}, \Gamma _0^{\circ },\Gamma _1^{\circ }\}$ be an ordinary boundary triplet for $A^*$ with the Weyl function *M* and the γ-field $\gamma ^\circ$, and let Γ from $({\mathfrak H}^\prime )^2$ to ${\mathcal {G}}^2$ be a boundary relation for $S^*$ with the Weyl family *Z* in ${\mathcal {G}}$. Let the boundary relation ${{\widehat{\Gamma }} }=\Gamma ^{\circ }{{\,\mathrm{\, \widehat{\oplus }\,}\,}}\Gamma ^\top$ be defined by (8.1). Then the linear relation

8.10 $$\begin{aligned} {{\widetilde{H}} }=\left\{ {{\widehat{f}} } \oplus {{\widehat{g}} } \in A^*{{\,\mathrm{\, \widehat{\oplus }\,}\,}}S^*:\, \psi '-\Gamma _1^{\circ }{{\widehat{f}} }=\psi +\Gamma _0^{\circ }{{\widehat{f}} }=0,\; \{{{\widehat{g}} },{{\widehat{\psi }} }\} \in \Gamma \right\} \end{aligned}$$

is a self-adjoint extension of $A{{\,\mathrm{\, \widehat{\oplus }\,}\,}}S$ in ${\mathfrak H}\oplus {\mathfrak H}^\prime$. Moreover, the compressed resolvent of ${{\widetilde{H}} }$ to H is given by

8.11 $$\begin{aligned} P_{\mathfrak H}({{\widetilde{H}} } {-}\lambda )^{-1}\upharpoonright {\mathfrak H}= (A_0 {-} \lambda )^{-1} {-} \gamma ^\circ (\lambda )(M(\lambda ){+}Z(\lambda ) )^{-1}\gamma ^\circ ( \bar{\lambda })^*, \quad \lambda \in {{\mathbb C}\setminus {\mathbb R}},\nonumber \\ \end{aligned}$$

where $A_0=\ker \Gamma _0^\circ$ is a self-adjoint extension of *A*.

#### Proof

First observe that ${{\widetilde{H}} }=\ker {{\widetilde{\Gamma }} }_0$ in Lemma 8.1 is determined by the conditions

8.12 $$\begin{aligned} \psi ' - \Gamma _1^{\circ }{{\widehat{f}} }=0 \quad \text{ and } \quad \psi +\Gamma _0^{\circ }{{\widehat{f}} }=0, \end{aligned}$$

where ${{\widehat{f}} } \oplus {{\widehat{g}} } \in A^*{{\,\mathrm{\, \widehat{\oplus }\,}\,}}S^*$, $\{{{\widehat{g}} },{{\widehat{\psi }} }\} \in \Gamma$. Therefore, ${{\widetilde{H}} }$ is as in (8.10) and it can be described with the original ${{\widehat{\Gamma }} }$ in (8.1). In fact, the boundary conditions in (8.12) can be rewritten as follows:

$$ \begin{pmatrix} \Gamma _1^{\circ }\widehat{f}\;\;\\ -\psi \end{pmatrix} =\begin{pmatrix} \psi ' \\ \Gamma _0^{\circ }\widehat{f}\; \end{pmatrix} = B \begin{pmatrix} \Gamma _0^{\circ }\widehat{f}\;\;\\ \psi ' \end{pmatrix}, \quad \text{ where } \quad B=\begin{pmatrix} 0 &  I_{\mathcal {G}}\\ I_{\mathcal {G}}&  0 \end{pmatrix}. $$

Using the boundary relation ${{\widehat{\Gamma }} }$ in (8.1) and identifying $B\in {\textbf{B}}({\mathcal {G}}^2)$ with its graph the previous equality can be expressed as follows:

8.13 $$\begin{aligned} \left\{ \begin{pmatrix} \Gamma _0^{\circ }\widehat{f}\;\;\\ \psi ' \end{pmatrix}, \begin{pmatrix} \Gamma _1^{\circ }\widehat{f}\;\;\\ -\psi \end{pmatrix} \right\} \in B. \end{aligned}$$

These are the conditions that determine the self-adjoint extension ${{\widetilde{H}} }$ of $A {{\,\mathrm{\, \widehat{\oplus }\,}\,}}S=\ker {{\widehat{\Gamma }} }$ and, in particular, ${{\widetilde{H}} }\subset {\text {dom }}{{\widehat{\Gamma }} }=A^* {{\,\mathrm{\, \widehat{\oplus }\,}\,}}{\mathcal {T}}$. Next define the relation ${{\widetilde{\Theta }} }$ by

$$ {{\widetilde{\Theta }} }=\left( {\text {ran }}{{\widehat{\Gamma }} }\cap B \right) {{\,\mathrm{\, \widehat{+} \,}\,}}\mathrm{mul\,}{{\widehat{\Gamma }} }. $$

It is clear that this is a direct sum as it follows from (8.3) that $\mathrm{mul\,}{{\widehat{\Gamma }} }\cap B=\{0\}$. Thus ${\text {ran }}{{\widehat{\Gamma }} }\cap B$ is the restriction of ${\text {ran }}{{\widehat{\Gamma }} }$ determined by the condition in (8.13), so that ${{\widetilde{H}} }={{\widehat{\Gamma }} }^{-1}({{\widetilde{\Theta }} })={{\widehat{\Gamma }} }^{-1}({\text {ran }}{{\widehat{\Gamma }} }\cap B)$. Now one can apply Theorem 7.1 to the self-adjoint extension ${{\widetilde{H}} }$ which shows that

8.14 $$\begin{aligned} ({{\widetilde{H}} } -\lambda )^{-1}-({{\widehat{S}} }_0-\lambda )^{-1} ={{\widehat{\gamma }} }(\lambda ) \bigl ({{\widetilde{\Theta }} }-{{\widehat{M}} }(\lambda )\bigr )^{-1} {{\widehat{\gamma }} }(\bar{\lambda })^*, \quad \lambda \in {{\mathbb C}\setminus {\mathbb R}}, \end{aligned}$$

where ${{\widehat{S}} }_0$ is given by (8.5), and ${{\widehat{\gamma }} }(\lambda )$, ${{\widehat{M}} }(\lambda )$ are given by (8.4).

In order to deal with the difference ${{\widehat{M}} }(\lambda )-{{\widetilde{\Theta }} }$ first observe that it follows from (8.2) and (8.3) that

$$ \begin{aligned} {{\widetilde{\Theta }} }&= \left( {\text {ran }}{{\widehat{\Gamma }} }\cap B \right) {{\,\mathrm{\, \widehat{+} \,}\,}}\mathrm{mul\,}{{\widehat{\Gamma }} }\\&= \left\{ \, \left\{ \left( {\begin{array}{c}\varphi \\ \psi '+\eta '\end{array}}\right) , \left( {\begin{array}{c}\psi '\\ \varphi - \eta \end{array}}\right) \right\} :\, \varphi \in {\mathcal {G}},\,\, {{\widehat{\psi }} }\in {\text {ran }}\Gamma , \,\, {{\widehat{\eta }} }\in \mathrm{mul\,}\Gamma \right\} . \end{aligned} $$

Moreover, (8.4) shows that $$\{\varphi ,\psi '\}\in {\text {dom }}{{\widehat{M}} }(\lambda )$$ also belongs to $${\text {dom }}{{\widetilde{\Theta }} }$$ (when taking $\eta^{\prime }=0$). Thus one can write $${{\widehat{M}} }(\lambda )-{{\widetilde{\Theta }} }$$ in the form

$$ {{\widehat{M}} }(\lambda )-{{\widetilde{\Theta }} }= \left\{ \left\{ \left( {\begin{array}{c}\varphi \\ \psi '\end{array}}\right) , \left( {\begin{array}{c}M(\lambda ) \varphi -\psi '\\ -\psi -\varphi +\eta \end{array}}\right) \right\} :\, \varphi \in {\mathcal {G}}, \, {{\widehat{\psi }} }\in Z(\lambda ), \, \{\eta ,0\} \in \mathrm{mul\,}\Gamma \right\} . $$

Here $\{\eta ,0\} \in \mathrm{mul\,}\Gamma \subset Z(\lambda )$ for all $\lambda \in {{\mathbb C}\setminus {\mathbb R}}$ by Lemma 3.4. Therefore, with $\{\psi ,\psi '\}$ also $\{{{\widetilde{\psi }} },\psi '\}$, with ${{\widetilde{\psi }} }=\psi -\eta$, span Z(λ). This means that

8.15 $$\begin{aligned} \begin{aligned}&({{\widetilde{\Theta }} }- {{\widehat{M}} }(\lambda ))^{-1} \\&\hspace{0.3cm}=\left\{ \, \left\{ \left( {\begin{array}{c}-M(\lambda ) \varphi + \psi '\\ \varphi +{{\widetilde{\psi }} }\end{array}}\right) , \left( {\begin{array}{c}\varphi \\ \psi '\end{array}}\right) \right\} :\, \varphi \in {\mathcal {G}}, \,\, \{{{\widetilde{\psi }} },\psi '\} \in Z(\lambda ) \right\} . \end{aligned} \end{aligned}$$

Hence, it follows from Lemma 2.5 and Lemma 8.1 that

8.16 $$\begin{aligned} ({{\widetilde{\Theta }} }- {{\widehat{M}} }(\lambda ))^{-1}= {{\widetilde{M}} }(\lambda ), \end{aligned}$$

where ${{\widetilde{M}} }(\lambda )\in {\textbf{B}}({\mathcal {G}}^2)$ is defined in (8.8).

Finally, substitution of (8.16) in the Kreĭn formula (8.14) gives

$$ ({{\widetilde{H}} } -\lambda )^{-1}-({{\widehat{S}} }_0-\lambda )^{-1} ={{\widehat{\gamma }} }(\lambda ){{\widetilde{M}} }(\lambda ){{\widehat{\gamma }} }(\bar{\lambda })^*, \quad \lambda \in {{\mathbb C}\setminus {\mathbb R}}. $$

It remains to compress this formula to the subspace H to get the stated resolvent formula (8.11). □

#### Remark 8.3

As a special case, consider boundary relations Γ from $({\mathfrak H}')^2$ to ${\mathcal {G}}^2$ that are purely multivalued, i.e., ${\text {ran }}\Gamma =\mathrm{mul\,}\Gamma$. In this case, the Weyl family satisfies $Z(\lambda )=\mathrm{mul\,}\Gamma$ and does not depend on $\lambda \in {{\mathbb C}\setminus {\mathbb R}}$ by Lemma 3.4. In particular, $Z(\lambda )=Z(\bar{\lambda })=Z(\lambda )^*$, i.e., Z(λ) is equal to a constant self-adjoint relation Θ in ${\mathcal {G}}$. Since ${\text {ran }}\Gamma$ is closed the same is true for its domain, so that ${\mathcal {T}}={\text {dom }}\Gamma =S^*$, where $S=\ker \Gamma$. Moreover, ${\mathcal {T}}=\Gamma ^{-1}({\text {ran }}\Gamma )=\Gamma ^{-1}(\mathrm{mul\,}\Gamma )=S$, so that $S=S^*$ is self-adjoint. Thus ${\mathfrak N}_\lambda (S^*)$ are trivial subspaces for all $\lambda \in {{\mathbb C}\setminus {\mathbb R}}$ and in Theorem 7.1 the condition $\mathrm{mul\,}\Gamma \subset \Theta \subset {\text {ran }}\Gamma$ means that there is only one extension $S_{\Theta }=S_0=S=S^*$. The Kreĭn type formulas in (7.1) and (7.2) reduce to a zero operator on ${\mathfrak H}'$; see Lemma 7.2. However, the coupling conditions in (8.10) that determine the self-adjoint relation ${{\widetilde{H}} }$ in Theorem 8.2 make sense, as they concern the values of Γ on the parameter space ${\mathcal {G}}$. Now in this special situation Z(λ) is a self-adjoint relation Θ in ${\mathcal {G}}$ and the compressed resolvent formula in (8.11) takes the form

$$ P_{\mathfrak H}({{\widetilde{H}} } -\lambda )^{-1}\upharpoonright {\mathfrak H}= (A_0 - \lambda )^{-1} - \gamma ^\circ (\lambda )(M(\lambda )+\Theta )^{-1}\gamma ^\circ ( \bar{\lambda })^*, \quad \lambda \in {{\mathbb C}\setminus {\mathbb R}}. $$

In fact, this is the standard Kreĭn’s formula which parametrizes all self-adjoint extensions of *A* in the original space H. Notice, that if the above purely multivalued boundary relation Γ for $S=S^*$ is minimal, then the exit space ${\mathfrak H}^\prime$ is trivial.

The Kreĭn formula in Theorem 8.2 has a long history. It was proved by M.G. Kreĭn [29] for densely defined symmetric operators with equal and finite defect numbers and by Saakyan [33] for equal infinite defect numbers. The proof of the Kreĭn formula for symmetric linear relations using the Cayley transform was given by Langer and Textorius [31] and Dijksma, Langer, and de Snoo in [24]. Another proof for symmetric linear relations with finite defect numbers based on the coupling method was presented in [13, Theorem 5.10], see also [19, Theorem 12.32] and [5, Notes on Section 2.7, Section 4.5, and Section 4.6] for more references.

### Exit Spaces and Generalized Resolvents

It is clear that the Kreĭn formula (8.11) provides a one-to-one correspondence between the Nevanlinna families *Z* and the exit space extensions of *A* via the coupling of boundary triplets and boundary relations. Another way to construct the corresponding exit space is provided in the following theorem in which a Nevanlinna family *Z* is interpreted as a Štraus family; cf. [5, Lemma 4.5.1 & Theorem 4.5.2]. The argument is based on the following simple lemma.

#### Lemma 8.4

Let *A* be a closed symmetric linear relation in a Hilbert space H and let $\{{\mathcal {G}}, \Gamma ^{\circ }_0, \Gamma ^{\circ }_1 \}$ be an ordinary boundary triplet for $A^*$ with Weyl function *M* and γ-field $\gamma ^\circ$. Let *Z* be a Nevanlinna family in ${\mathcal {G}}$ with corresponding Nevanlinna pair $Z=\{{\mathcal {A}},{\mathcal {B}}\}$ and define for each $\lambda \in {{\mathbb C}\setminus {\mathbb R}}$,

8.17 $$\begin{aligned} R(\lambda )= (A_0 - \lambda )^{-1} - \gamma ^{\circ }(\lambda )(M(\lambda )+Z(\lambda ) )^{-1}\gamma ^{\circ }( \bar{\lambda })^*, \quad \lambda \in {{\mathbb C}\setminus {\mathbb R}}. \end{aligned}$$

Then R(λ), $\lambda \in {{\mathbb C}\setminus {\mathbb R}}$, is a well defined operator in ${\textbf{B}}({\mathfrak H})$ and it is a generalized resolvent, satisfying $\mathrm {(R1)}$, $\mathrm {(R2)}$, and $\mathrm {(R3)}$. In fact,

8.18 $$\begin{aligned} \textsf{R}_R(\lambda , \mu )=W(\lambda ) \textsf{N}_{{\mathcal {A}},{\mathcal {B}}}(\lambda , \mu )W(\mu )^*, \quad \lambda , \mu \in {{\mathbb C}\setminus {\mathbb R}}, \end{aligned}$$

where

8.19 $$\begin{aligned} W(\lambda )=\gamma ^{\circ }(\lambda ) (M(\bar{\lambda }){\mathcal {A}}(\bar{\lambda })+{\mathcal {B}}(\bar{\lambda }))^{-*}. \end{aligned}$$

#### Proof

Use the notation $R_0(\lambda )=(A_0-\lambda )^{-1}$ and write the right-hand side of (8.17) as

8.20 $$\begin{aligned} R(\lambda )=R_0(\lambda )- \gamma ^{\circ }(\lambda )Q(\lambda )\gamma ^{\circ }(\bar{\lambda })^* \quad \text{ with } \quad Q(\lambda )=(M(\lambda )+Z(\lambda ))^{-1}. \end{aligned}$$

Then it is clear that $\mathrm {(R1)}$ and $\mathrm {(R3)}$ are satisfied. To verify $\mathrm {(R2)}$ the kernel $\textsf{R}_R(\lambda , \mu )$ will be rewritten. For this observe that (6.10) and (6.9) with $\lambda , \mu \in {{\mathbb C}\setminus {\mathbb R}}$ give

8.21 $$\begin{aligned} R_0(\lambda ) \gamma (\bar{\mu }) =\frac{\gamma ^\circ (\lambda )-\gamma ^\circ (\bar{\mu })}{\lambda -\bar{\mu }}, \quad \gamma (\bar{\lambda })^* R_0(\mu )^* =\frac{\gamma ^\circ (\bar{\lambda })^*-\gamma ^\circ (\mu )^*}{\lambda -\bar{\mu }}, \end{aligned}$$

and

8.22 $$\begin{aligned} \frac{M(\lambda )-M(\mu )^*}{\lambda -\bar{\mu }}= \gamma ^\circ (\bar{\lambda })^*\gamma ^\circ (\bar{\mu }), \quad \lambda , \mu \in {{\mathbb C}\setminus {\mathbb R}}. \end{aligned}$$

Straightforward calculation then gives

8.23 $$\begin{aligned} \textsf{R}_R(\lambda , \mu )=\frac{1}{\lambda -\bar{\mu }}\gamma ^{\circ }(\lambda ) \big [Q(\mu )^*-Q(\lambda ) -Q(\lambda )(M(\lambda )-M(\mu )^*)Q(\mu )^*\big ]\gamma ^{\circ }(\mu )^*. \end{aligned}$$

Furthermore, by using the representation $Z=\{{\mathcal {A}},{\mathcal {B}}\}$, it follows from (2.6) that

$$ Q(\lambda )={\mathcal {A}}(\lambda )(M(\lambda ){\mathcal {A}}(\lambda )+{\mathcal {B}}(\lambda ))^{-1}. $$

Hence using the property $Q(\lambda )=Q(\bar{\lambda })^*$ in the left-hand side of the following identity one sees directly that

$$\begin{aligned} Q(\mu )^*-Q(\lambda )&=Q(\bar{\mu })- Q(\bar{\lambda })^*\\&=Q(\lambda )(M(\lambda )-M(\mu )^*)Q(\mu )^* \\&\quad +(M(\bar{\lambda }){\mathcal {A}}(\bar{\lambda })+{\mathcal {B}}(\bar{\lambda }))^{-*} [{\mathcal {B}}(\bar{\lambda })^* {\mathcal {A}}(\bar{\mu })-{\mathcal {A}}(\bar{\lambda })^*{\mathcal {B}}(\bar{\mu })]\\&\qquad \times (M(\bar{\mu }){\mathcal {A}}(\bar{\mu })+{\mathcal {B}}(\bar{\mu }))^{-1}. \end{aligned}$$

Substitution of this identity into (8.23) leads to (8.18) and (8.19). This confirms $\mathrm {(R2)}$. □

#### Theorem 8.5

Let *A* be a closed symmetric linear relation in a Hilbert space H and let $\{{\mathcal {G}}, \Gamma ^{\circ }_0, \Gamma ^{\circ }_1 \}$ be an ordinary boundary triplet for $A^*$. Let *Z* be a Nevanlinna family in ${\mathcal {G}}$ and let *R* be the family of generalized resolvents as in (8.17). Let ${\mathfrak H}(\textsf{R}_{R})$ be the associated reproducing kernel Hilbert space. Then ${{\widetilde{H}} }_{R}$ in (5.10) is a self-adjoint extension of *A* in the Hilbert space ${\mathfrak H}(\textsf{R}_R) \oplus {\mathfrak H}$. Moreover, the compressed resolvent of ${{\widetilde{H}} }_R$ onto H satisfies

$$ P_{\mathfrak H}({{\widetilde{H}} }_R-\lambda )^{-1} \upharpoonright {\mathfrak H}=R(\lambda ), \quad \lambda \in {{\mathbb C}\setminus {\mathbb R}}. $$

Furthermore, the self-adjoint relation ${{\widetilde{H}} }_R$ satisfies the following minimality condition

$$ {\mathfrak H}(\textsf{R}_{R}) \oplus {\mathfrak H}=\overline{\textrm{span}} \, \bigl \{{\mathfrak H}, ({{\widetilde{H}} }_{R}-\lambda )^{-1} {\mathfrak H}:\, \lambda \in {{\mathbb C}\setminus {\mathbb R}}\bigr \}. $$

#### Proof

All but one of the assertions follow from Lemma 5.2. The only assertion that needs explanation is that ${{\widetilde{H}} }_R$ extends *A*. For this observe that (8.17) shows

$$ \begin{aligned} \big \{ \{R(\lambda )h, (I+\lambda R(\lambda ))h\} :\, h \in {\mathfrak H}\big \}&\subset \big \{ \{R_0(\lambda )h, (I+\lambda R_0(\lambda ))h\}:\, h \in {\mathfrak H}\big \} \\&+\big \{ \{ \gamma ^\circ (\lambda )\varphi , \lambda \gamma ^\circ (\lambda )\varphi \} :\, \varphi \in {\mathcal {G}}\big \}. \end{aligned} $$

Since each of the relations in the right-hand side belongs to $A^*$, it follows that $T(\lambda ) \subset A^*$. By Lemma 5.3 this implies that $A \subset {{\widetilde{H}} }_R$. □

Based on (8.18) in Lemma 8.4 multiplication by the operator-valued function *W* in (8.19) sets up a unitary equivalence between the corresponding reproducing kernel Hilbert spaces. This gives a connection between the model associated with Theorem 8.5 and Lemma 5.2 via

$$ U: \begin{pmatrix} {\mathfrak H}(\textsf{N}_{{\mathcal {A}},{\mathcal {B}}}) \\ {\mathfrak H}\end{pmatrix} \rightarrow \begin{pmatrix} {\mathfrak H}(\textsf{R}_{R}) \\ {\mathfrak H}\end{pmatrix}, \quad \quad \begin{pmatrix} f(\xi ) \\ h \end{pmatrix} \mapsto \begin{pmatrix} W(\xi ) f(\xi ) \\ h \end{pmatrix}, \quad $$

where $$f \in {\mathfrak H}(\textsf{N}_{{\mathcal {A}},{\mathcal {B}}})$$ and $$h \in {\mathfrak H}$$.

## Data Availability

No data were collected, generated or consulted in connection with this research.

## Appendix A. Coupling Method in the Framework of *Q*-Functions

The notion of the *Q*-function was the key element in the theory of self-adjoint extensions in the works of Kreĭn and Langer. For densely defined symmetric operators with equal defect numbers, the *Q*-function was first introduced by M.G. Kreĭn [29] when the defect numbers are finite and by Saakyan [33] when the defect numbers are infinite. In [31] Langer and Textorius have considered *Q*-functions in the context of symmetric relations. The *Q*-function plays a central role in describing the resolvents of self-adjoint extensions of symmetric operators via the Kreĭn’s resolvent formula (cf. Theorem 8.2), where originally a *Q*-function of the self-adjoint extension $A_0$ of *A* was used instead of the Weyl function *M* of an associated boundary triplet. The Nevanlinna family *Z* in (8.11) was used to generate the exit space ${\mathfrak H}^\prime$ via the so-called ϵ-method as well as to describe the self-adjoint extension ${{\widetilde{H}} }$ in ${\mathfrak H}\oplus {\mathfrak H}^\prime$; see e.g. [31]; cf. Theorem 8.5.

Initially the coupling method was considered in the framework of *Q*-functions for symmetric operators with finite defect numbers; see [25–27]. This approach is now briefly recalled. The key objects in this method are as follows. Let *A* be a densely defined closed symmetric operator in H with equal defect numbers. Let $A_0$ be a self-adjoint extension of *A* in H. Fix a point $\mu \in {{\mathbb C}\setminus {\mathbb R}}$ and a bijective mapping $\gamma ^\circ (\mu )$ from a Hilbert space ${\mathcal {G}}$ onto the eigenspace ${\mathfrak N}_\mu (A^*)$ of $A^*$. Then define the γ-field by the formula

A.1 $$\begin{aligned} \gamma ^\circ (\lambda )=(I+(\lambda -\mu )(A_0-\lambda )^{-1})\gamma ^\circ (\mu ), \quad \lambda \in {{\mathbb C}\setminus {\mathbb R}}, \end{aligned}$$

see also (6.10). It is easy to check that $\gamma ^\circ (\lambda )$ maps ${\mathcal {G}}$ onto the eigenspace ${\mathfrak N}_\lambda (A^*)$ for each $\lambda \in {{\mathbb C}\setminus {\mathbb R}}$ by means of the formula

A.2 $$\begin{aligned} \bigl ( (A-\lambda )f,\gamma ^\circ (\bar{\lambda })h\big )_{\mathfrak H}=0 \; \text { for all } f\in {\text {dom }}A,\; h\in {\mathcal {G}}. \end{aligned}$$

The corresponding *Q*-function is then defined as a ${\textbf{B}}({\mathcal {G}})$-valued function via the formula

A.3 $$\begin{aligned} \gamma ^\circ (\mu )^*\gamma ^\circ (\lambda )=\frac{Q(\lambda )-Q(\mu )^*}{\lambda -\bar{\mu }},\quad \lambda ,\mu \in {{\mathbb C}\setminus {\mathbb R}}, \end{aligned}$$

cf. (6.9). The formula (A.3) determines the *Q*-function uniquely up to a self-adjoint operator in ${\textbf{B}}({\mathcal {G}})$. The *Q*-function is a uniformly strict operator-valued Nevanlinna function, since $\gamma ^\circ (\lambda )$ in (A.3) is a bounded mapping with bounded inverse for all $\lambda \in {{\mathbb C}\setminus {\mathbb R}}$. The resolvents of all self-adjoint extensions of *A* in the initial Hilbert space H are parametrized by means of the self-adjoint relations τ in ${\mathcal {G}}$ via Kreĭn’s formula

A.4 $$\begin{aligned} (A_\tau - \lambda )^{-1} = (A_0 - \lambda )^{-1} -\gamma ^\circ (\lambda )(Q(\lambda )+\tau )^{-1}\gamma ^\circ (\bar{\lambda })^*,\quad \lambda \in {{\mathbb C}\setminus {\mathbb R}}. \end{aligned}$$

Similarly, let *S* be a densely defined symmetric operator in another Hilbert space ${\mathfrak H}'$ whose defect numbers coincide with those of *A*. Again, fix a self-adjoint extension $S_0$ of *S* in ${\mathfrak H}'$ and define the corresponding γ-field $\gamma '(\lambda )$ as a mapping from ${\mathcal {G}}$ onto ${\mathfrak N}_\lambda (S^*)$ as in (A.1). Let Z(λ) be an associated *Q*-function as in (A.3). Then the resolvents of the self-adjoint extensions $S_\eta$ of *S* in ${\mathfrak H}'$ are parametrized via Kreĭn’s formula by means of the self-adjoint relations η in ${\mathcal {G}}$

A.5 $$\begin{aligned} (S_\eta - \lambda )^{-1}=(S_0 - \lambda )^{-1} - \gamma '(\lambda )(Z(\lambda )+\eta )^{-1}\gamma '(\bar{\lambda })^*, \quad \lambda \in {{\mathbb C}\setminus {\mathbb R}}. \end{aligned}$$

Now introduce in the Hilbert space ${\mathfrak H}\oplus {\mathfrak H}'$ the orthogonal sum ${{\widehat{A}} }=A{{\,\mathrm{\, \widehat{\oplus }\,}\,}}S$, which is a densely defined closed symmetric operator with equal (but twice as large) defect numbers. Then ${{\widehat{A}} }_0=A_0 {{\,\mathrm{\, \widehat{\oplus }\,}\,}}S_0$ is a self-adjoint extension of ${{\widehat{A}} }$ with γ-field ${{\widehat{\gamma }} }(\lambda )=\gamma (\lambda ){{\,\mathrm{\, \widehat{\oplus }\,}\,}}\gamma '(\lambda )$ and *Q*-function ${{\widehat{Q}} }(\lambda )=Q(\lambda ){{\,\mathrm{\, \widehat{\oplus }\,}\,}}Z(\lambda )$. Combining also the resolvent formulas (A.4) and (A.5) orthogonally, one obtains

A.6 $$\begin{aligned} \begin{aligned}&((A_\tau {{\,\mathrm{\, \widehat{\oplus }\,}\,}}S_\eta ) - \lambda )^{-1}=({{\widehat{A}} }_0 - \lambda )^{-1} \\&\quad - \begin{pmatrix} \gamma ^\circ (\lambda ) &  0 \\ 0 &  \gamma '(\lambda ) \end{pmatrix} \begin{pmatrix} Q(\lambda ) + \tau &  0 \\ 0 &  Z(\lambda )+\eta \end{pmatrix}^{-1} \begin{pmatrix} \gamma ^\circ (\bar{\lambda })^* &  0 \\ 0 &  \gamma '(\bar{\lambda })^* \end{pmatrix}. \end{aligned} \end{aligned}$$

The next crucial step is the coupling of the associated γ-fields which act in orthogonal Hilbert spaces in (A.6). For instance, let *C* and *D* be bounded and boundedly invertible operators in ${\mathcal {G}}$ and define the function $\gamma _U(\lambda )$ as follows:

A.7 $$\begin{aligned} \gamma _U(\lambda )= \begin{pmatrix} \gamma ^\circ (\lambda ) &  0 \\ 0 &  \gamma '(\lambda ) \end{pmatrix}\left( {\begin{array}{c}C\\ D\end{array}}\right) =\begin{pmatrix} \gamma ^\circ (\lambda )C \\ \gamma '(\lambda )D \end{pmatrix}, \quad \lambda \in {{\mathbb C}\setminus {\mathbb R}}. \end{aligned}$$

It is clear that $\gamma _U(\lambda )$ satisfies the analog of the identity (A.1) with the self-adjoint extension ${{\widehat{A}} }_0$ and that

A.8 $$\begin{aligned} Q_U(\lambda )=C^*Q(\lambda )C+D^*Z(\lambda )D, \quad \lambda \in {{\mathbb C}\setminus {\mathbb R}}, \end{aligned}$$

is a uniformly strict Nevanlinna function acting on ${\mathcal {G}}$, which satisfies the analog of (A.3). Introduce the restriction $S_U$ of ${{\widehat{A}} }_0=A_0 {{\,\mathrm{\, \widehat{\oplus }\,}\,}}S_0$ by the analog of the condition in (A.2):

$$ \biggl (\left( {\begin{array}{c}(A-\lambda )f\\ (S-\lambda )g\end{array}}\right) , \begin{pmatrix} \gamma ^\circ (\bar{\lambda })C \\ \gamma '(\bar{\lambda })D \end{pmatrix}h \biggr )_{{\mathfrak H}\times {\mathfrak H}'}=0, \quad f\in {\text {dom }}A,\; g\in {\text {dom }}S, \; h\in {\mathcal {G}}. $$

By construction, it is clear that the symmetric operator $S_U$ does not depend on $\lambda \in {{\mathbb C}\setminus {\mathbb R}}$, due to the analog of the formula (A.2). Now observe that $\gamma _U$ in (A.7) is the γ-field for the symmetric operator $S_U$ with the *Q*-function $Q_U$ in (A.8) and that $S_U$ is a proper extension of the orthogonal sum of the symmetric operator ${{\widehat{A}} }=A{{\,\mathrm{\, \widehat{\oplus }\,}\,}}S$. Therefore, one has Kreĭn’s formula in ${{\widetilde{\mathfrak H}} }={\mathfrak H}\oplus {\mathfrak H}'$ for the self-adjoint extensions of $S_U$ in ${\mathfrak H}\oplus {\mathfrak H}'$ via the self-adjoint relations ${{\widetilde{\tau }} }$ in ${\mathcal {G}}$

$$ ({{\widetilde{A}} }_{{{\widetilde{\tau }} }} - \lambda )^{-1}=({{\widehat{A}} }_0 - \lambda )^{-1} - \begin{pmatrix} \gamma ^\circ (\lambda )C \\ \gamma '(\lambda )D \end{pmatrix}(Q_U(\lambda )+{{\widetilde{\tau }} })^{-1} \begin{pmatrix} \gamma ^\circ (\bar{\lambda })C \\ \gamma '(\bar{\lambda })D \end{pmatrix}^*, $$

cf. [5, Proposition 4.6.1], whose compression to H gives

$$ \begin{aligned} P_{\mathfrak H}({{\widetilde{A}} }_{{{\widetilde{\tau }} }} - \lambda )^{-1}\upharpoonright {\mathfrak H}&=(A_0 - \lambda )^{-1} \\&\quad -\gamma ^\circ (\lambda )C \bigl (C^*Q(\lambda )C+D^*Z(\lambda )D +{{\widetilde{\tau }} }\bigr )^{-1}C^*\gamma ^\circ (\bar{\lambda })^*. \end{aligned} $$

In particular, with the choice C=D=I one gets the formula

$$ P_{\mathfrak H}({{\widetilde{A}} }_{{{\widetilde{\tau }} }} - \lambda )^{-1}\upharpoonright {\mathfrak H}=(A_0 - \lambda )^{-1} - \gamma ^\circ (\lambda )\bigl (Q(\lambda )+Z(\lambda ) +{{\widetilde{\tau }} }\bigr )^{-1}\gamma ^\circ (\bar{\lambda })^*. $$

Observe, from this last resolvent formula that for all self-adjoint relations ${{\widetilde{\tau }} }$ in ${\mathcal {G}}$ the corresponding Nevanlinna functions $Z(\lambda )+\eta$ in (A.6) (take τ=0) (or the corresponding Štraus families in Sect. 5) originate from the same exit space ${\mathfrak H}'$ using the present coupling technique via γ-fields. This reflects one of the main differences between the coupling technique for *Q*-functions and the Weyl functions of boundary triplets, since the description of the coupling conditions for the associated boundary mappings depend on the choice of the self-adjoint parameters τ and η, while the *Q*-functions *Q* and *Z* allow a free choice for them as self-adjoint additive terms in (A.6). Of course, the particular choice $\tau =\eta =0$ gives Kreĭn’s formula as in Theorem 8.2 for exit space extensions $\widetilde{H}$ of *A* generated by Z(λ).
